# Indicators of "Healthy Aging" in older women (65-69 years of age). A data-mining approach based on prediction of long-term survival

**DOI:** 10.1186/1471-2318-10-55

**Published:** 2010-08-17

**Authors:** William R Swindell, Kristine E Ensrud, Peggy M Cawthon, Jane A Cauley, Steve R Cummings, Richard A Miller

**Affiliations:** 1 Department of Pathology, University of Michigan, School of Medicine, Ann Arbor, MI, 48109-2200, USA; 2 Geriatrics Center, University of Michigan, School of Medicine, Ann Arbor, MI, 48109-2200, USA; 3 University of Minnesota, Minneapolis, MN, USA; 4 California Pacific Medical Center Research Institute, San Francisco, CA, 94120-7999, USA; 5 Department of Epidemiology, University of Pittsburgh, Pittsburgh, PA, 15260, USA; 6 VA Medical Center, University of Michigan, School of Medicine, Ann Arbor, MI, 48109-2200, USA

## Abstract

**Background:**

Prediction of long-term survival in healthy adults requires recognition of features that serve as early indicators of successful aging. The aims of this study were to identify predictors of long-term survival in older women and to develop a multivariable model based upon longitudinal data from the Study of Osteoporotic Fractures (SOF).

**Methods:**

We considered only the youngest subjects (*n *= 4,097) enrolled in the SOF cohort (65 to 69 years of age) and excluded older SOF subjects more likely to exhibit a "frail" phenotype. A total of 377 phenotypic measures were screened to determine which were of most value for prediction of long-term (19-year) survival. Prognostic capacity of individual predictors, and combinations of predictors, was evaluated using a cross-validation criterion with prediction accuracy assessed according to time-specific AUC statistics.

**Results:**

Visual contrast sensitivity score was among the top 5 individual predictors relative to all 377 variables evaluated (mean AUC = 0.570). A 13-variable model with strong predictive performance was generated using a forward search strategy (mean AUC = 0.673). Variables within this model included a measure of physical function, smoking and diabetes status, self-reported health, contrast sensitivity, and functional status indices reflecting cumulative number of daily living impairments (HR ≥ 0.879 or RH ≤ 1.131; P < 0.001). We evaluated this model and show that it predicts long-term survival among subjects assigned differing causes of death (e.g., cancer, cardiovascular disease; P < 0.01). For an average follow-up time of 20 years, output from the model was associated with multiple outcomes among survivors, such as tests of cognitive function, geriatric depression, number of daily living impairments and grip strength (P < 0.03).

**Conclusions:**

The multivariate model we developed characterizes a "healthy aging" phenotype based upon an integration of measures that together reflect multiple dimensions of an aging adult (65-69 years of age). Age-sensitive components of this model may be of value as biomarkers in human studies that evaluate anti-aging interventions. Our methodology could be applied to data from other longitudinal cohorts to generalize these findings, identify additional predictors of long-term survival, and to further develop the "healthy aging" concept.

## Background

Prediction of long-term survivorship and health outcomes among older individuals depends upon the recognition of factors that contribute to healthy aging. Individuals that exhibit healthy aging patterns maintain high quality of life into late stages of the life span, with few daily living impairments and a near absence of age-related disease [1,2]. On the other hand, an unhealthy aging trajectory is associated with the onset of comorbid diseases, diminished quality of life, and increased mortality risk [1,2]. From the standpoint of geriatric care, the ability to predict future changes in health is of great importance, since this can improve the efficiency and quality of care by identifying at-risk individuals, helping to target preventative measures most effectively [3]. Development of prognostic models can also provide new tools for investigations into aging mechanisms. One view, for instance, suggests that variation in aging outcomes is attributable to different rates of aging, with increased healthspan in some individuals due to a reduced "biological" relative to chronological age [4-9]. If this notion is correct, quantitative characterization of the "biological age" construct should provide a powerful prognostic device, and statistical models optimized to predict long-term survival should, by direct design or not, be based upon measures that most closely track the progression of aging [10]. This reasoning suggests that construction of predictive models can point towards aging biomarkers, which are essential for investigating aging mechanisms in long-lived organisms, for which survival is not a practical endpoint for scientific study. In humans, progress along these lines would be of great value, since clinical trials have been initiated or completed for evaluation of several anti-aging interventions [11-13], but there is no consensus on which experimental measures are in fact most suitable for detection of decelerated aging.

Healthy aging should be recognizable based upon a well-chosen set of measurements from a broad range of domains, which collectively reflect the multifaceted effects of aging, and the interdependence of these effects among organ systems [4]. Currently, however, while multiple sets of measurements have been proposed as indices of "frailty" in the elderly [14-22], there is no analogous set of measures to define a healthy aging phenotype in middle-aged or otherwise healthy adults. The absence of an objective and well-validated summary measure of health status has placed limits on insights derived from well-designed studies. Caloric restriction, for example, may slow the rate of intrinsic aging in middle-aged adults, and clinical investigations have shown that this intervention induces favorable shifts of individual variables, including body mass index, lipid profiles, body fat, inflammatory markers and insulin sensitivity [23]. It is unclear, however, which of these measures are most robustly associated with long-term survival, which are linked to one or instead to multiple disease processes, which provide independent versus redundant information, and which might ultimately constitute a combination of variables that best characterizes the "healthy aging" concept. To some degree, measures that best characterize "healthy aging" are likely to overlap with those that characterize "frailty", such that indices that identify "frail" individuals may also serve to detect healthy aging as well [24]. At the same time, however, some pre-frail and apparently healthy adults may not yet exhibit age-related disease traits and the advanced deficit accumulation to which many frailty measures are particularly sensitive. Potentially, therefore, standard measures of frailty may not provide optimal tools for characterization of "healthy aging" in younger cohorts that exclude the oldest old. A well-known frailty index, for example, did not include a measure that directly relates to individual smoking or diabetes status [16], but these are certainly key factors underlying aging trajectory and long-term survivorship.

Measures that best characterize "frailty" have often been validated based on their ability to predict survival with respect to heterogeneous populations that include healthy younger subjects in combination with frailer older subjects [16,21,22]. In the study of Fried et al. [16], for example, a frailty index was validated based upon its ability to predict short-term (8-year) survival among subjects that varied between 65 and 101 years of age. For characterization of healthy aging, we suggest that greater emphasis be placed on prediction of more long-term survival outcomes with respect to reasonably healthy cohorts in which few subjects suffer from advanced age-related disease [4,25]. Given this criteria, there are numerous variables that, based upon prior epidemiological studies, might conceivably be included within a "healthy aging" index. Simple body composition measures, such as body mass index and waist circumference, are easily obtained during a clinical interview, and are widely used as informal indicators of mortality risk from cardiovascular disease and cancer [26-32]. The most useful variables, however, may in principle not be directly linked to any single disease process, but might instead reflect a subject's overall health and fitness [33-35]. These measures, which have generally not been evaluated in human studies of anti-aging interventions, include tests of mental function [36], hand-grip strength [37], visual acuity [38], walking ability [39], or indices based upon total accumulation of "deficits" or health disorders [19,34,35,40-43]. A wide range of measures at cellular and physiological levels, such as blood glucose [44], telomere length [45], inflammation markers [46], and bone turnover rates [47], could potentially provide further information regarding future health status of subjects. Moreover, in humans, feedback generated from standardized questionnaires can provide inexpensive, easily obtained, and potentially valuable prognostic data for prediction of both lifespan and healthspan [48,49]. Given all the variables previously shown to predict survival in certain cohorts, a key challenge is to establish the *relative *value of individual variables, and to isolate those that can be used to form a parsimonious predictive model. For this purpose, data mining methods applied to large datasets are especially useful [10], and such approaches can be used to derive evidence-based indices that will ultimately bolster efficiency in both clinical and research settings [50-54].

The goal of this study was to identify variables useful for prediction of long-term survival within a cohort of women between the ages of 65 and 69, and to develop a multivariate index of healthy aging that can potentially provide a clinical and research tool. We analyze data from 4,097 women (65 to 69 years of age) enrolled between 1986 and 1988 in the Study of Osteoporotic Fractures (SOF) and followed prospectively over an average of 16.1 years for mortality. This cohort was well-suited for our investigation, since most subjects were healthy at baseline examinations, with low incidence of advanced age-related disease. Additionally, all subjects in the SOF study had been evaluated with respect to hundreds of variables relating to body composition, visual acuity, physical performance and function, demographic measures, mental health, lifestyle practices, current and previous medication use, and family history. The data we consider thus represent a valuable resource for establishing the relative prognostic value of numerous phenotypic traits, and for identifying indices of variables that best predict long-term survival, which may lead to standardized criteria to characterize the phenotype that precedes successful aging.

## Methods

### Study Population

The Study of Osteoporotic Fractures (SOF) is a prospective cohort study of community-dwelling Caucasian women over the age of 65. Participants were recruited between September 1986 and October 1988 based upon community-based listings (e.g., health plan membership lists, voter registration lists, and Department of Motor Vehicle tapes) from four separate regions in the United States (The Kaiser Foundation Research Institute and Center for Health Research, Portland, Oregon; The Epidemiology Clinical Research Center at the University of Minnesota, Minneapolis, Minnesota; Monongahela Valley Clinic of the University of Pittsburgh, Monessen, Pennsylvania; and The University of Maryland Osteoporosis Clinic, Baltimore, Maryland). The SOF was originally designed to identify risk factors for osteoporotic fractures, and therefore excluded women unable to walk without assistance and women with bilateral hip replacements. Black women were also excluded from the baseline exam due to their low incidence of hip fracture. All subjects provided written consent at entry into the study and at each clinical examination. The protocol and consent form were approved by the institutional review boards (IRB) at all participating institutions (i.e., Kaiser Permanente Northwest IRB; University of Pittsburgh IRB; University of Minnesota IRB; California Pacific Medical Center IRB; University of Maryland, School of Medicine Human Research Protections Office; University of California, San Francisco Committee on Human Research).

The complete SOF cohort included 9704 women between the ages of 65 and 89, with a median age of 70 years (Additional File 1). Since the main purpose of our analysis was to identify factors associated with healthy aging and long-term survivorship, it was desirable to exclude older subjects, since this group was much more likely to be short-lived and more likely to exhibit symptoms associated with frailty [16]. Our analysis therefore focuses on the 4097 SOF participants less than 70 years of age at the baseline examination (Additional File 1). Mortality assessments were completed in this cohort at 4-month intervals by mail or phone contact of participants (or family member proxies). For each death, an official death certificate was obtained and, when possible, a hospital discharge summary as well. This information was used by one or more epidemiologists to assign cause of death, in accordance with *International Classification of Disease, Ninth Revision, Clinical Modification *(ICD9) codes. It should be noted that, in general, some level of classification error is likely in the assignment of ICD9 codes, particularly among older individuals in whom comorbidities are frequent. Nevertheless, atherosclerosis was the most frequently assigned cause of death, and was assigned in 467 of 1523 (30.7%) documented cases (ICD9 = 425; ICD9 = 429.2; 440 ≤ ICD9 < 445; ICD9 = 428; 401 ≤ ICD9 < 405; 410 ≤ ICD9 < 415; ICD9 = 798; 430 ≤ ICD9 < 439. Cancer deaths were also common, and cancer was assigned as the cause in 426 of 1523 (27.8%) of documented deaths (140 ≤ ICD9 < 240). More than one-third (38.5%) of all deaths, however, were attributable to neither to atherosclerosis nor cancer (586 of 1523 deaths). There were 25 deaths due to injury and poisoning (ICD9 > = 800) and an additional 19 deaths due to other external causes of injury (ICD9 E800-E990), and in these cases, the survival times of subjects were right-censored (Additional File 1).

### Predictor Variables and Data Pre-processing

After recruitment of the SOF cohort, study participants have periodically returned to clinical centers for as many as nine visits. In our analyses, however, we have utilized only baseline variable measurements recorded in the first visit of each SOF participant (occurring between September 1986 and October 1988). All variables included in our analysis, therefore, are obtainable during a single clinical examination and interview. These variables can roughly be categorized as anthropomorphic (e.g., height), demographic (e.g., age and race), blood pressure and pulse measures, cognitive function (i.e., short mini mental status exam), family history, female history (e.g., prior pregnancies, hysterectomy), physical function and performance (e.g., number of seconds to complete 5 stands), medical history, medication inventory, lifestyle survey (e.g., caffeine use) and evaluation of vision (e.g., contrast sensitivity scores). Altogether, the complete dataset included measurements of 465 variables on the 4097 subjects. We screened these variables, and excluded from further analysis those with more than 5% missing values. Following this pre-processing, there remained 300 variables (146 continuous, 28 ordinal and 126 categorical) upon which further analyses were based. We applied Grubb's test to each continuous variable to check for the presence of outliers [55], and log-transformed 77 continuous variables for which there was strong evidence indicating that at least one outlying observation was present (Grubb's test; P < 10^-3^). All categorical variables with *n *= 2 classes were coded as a single 0-1 indicator variable. For categorical variables with *n *> 2 classes, we recoded the variable as a set *n *of 0-1 indicator variables, with a single 0-1 indicator variable representing each of the *n *classes. We do not use *n *- 1 indicator variables in such cases, because prior to variable selection, it was uncertain which indicators would be selected in a final model, and it was expected that our variable selection strategy would ultimately identify an appropriate (and non-redundant) subset of the *n *indicators for any categorical variable with *n *> 2 classes. This recoding of categorical variables yielded a total of 203 categorical variables (each with 0-1 coding). The complete analysis, therefore, was based upon a total of 377 variables (146 continuous, 28 ordinal and 203 categorical). Further pre-processing of variables to eliminate those highly correlated with other variables was unnecessary at this stage, since this would be accomplished as part of model-building procedures. A complete list of the 377 variables, along with 88 excluded from the analysis, is provided as supplementary data (see Additional File 2).

All variables included in the analysis contained fewer than 5% missing data, and most variables included much less than 5% missing data. Among all variables we considered, missing values accounted for, on average, only 0.40% of data entries. For these missing data, it was not feasible to perform multiple imputations, given that our analyses were computationally expensive (see below). We therefore employed a *k*-nearest-neighbor imputation approach [56], in which missing values were replaced based upon the *k *= 20 nearest neighbors of a given subject for which data was missing. The nearest neighbors of each subject were identified based upon the Euclidean distance across all variables. These variables included both continuous and categorical variables, of which the Euclidean distance is meaningful only for the former. For the purpose of nearest-neighbor computations, therefore, categorical variables were expressed as 8 standard coordinates obtained by multiple correspondence analysis (with retention of 70% of the variance associated with the entire set of 126 categorical variables). Once nearest neighbors were identified for a subject with missing data, we imputed missing data using the average value among the *k *= 20 neighbors for continuous variables, the median value among the *k *= 20 neighbors for ordinal variables, and the modal value among the *k *= 20 neighbors for categorical variables.

### Statistical Assessment of Prognostic Value

One criterion for evaluating an index for healthy aging is that the index should discriminate among subjects based upon their observed survival times. For an index generated from a Cox regression model, this output corresponds to a "risk score" (i.e., linear predictor), which is proportional to the predicted mortality risk of a subject relative to all other subjects. This risk score is a useful diagnostic index if subjects assigned high scores tend to be short-lived, and if subjects assigned low scores are usually long-lived. Along these lines, we evaluated predictive capacity of models based upon a cross-validation criterion, in which a model is first constructed using one set of subjects (i.e., a training set), and then used to generate risk scores for a distinct set of subjects (i.e., a testing set). The predictive capacity of the model is measured according to how well risk scores discriminate among short and long-lived subjects within the testing set. In our analyses, training data consisted of randomly selected sets of 3687 subjects (90% of cohort), while testing data consisted of the remaining 410 subjects (10% of cohort). Prognostic capacity is measured according to a concordance index (based on time-specific area under the curve (AUC) statistics; see below), which measures the degree to which high risk scores are assigned to short-lived subjects, and the degree to which low scores are assigned to long-lived subjects. For a given model, this process is repeated 10,000 times, and the average concordance score among all simulation trials is computed. This cross-validation approach, involving random splitting of subjects into "training" and "testing" sets, provides a good indication of how well the model characterizes meaningful patterns in the data that are useful for prediction, rather than random noise [57].

Correspondence between model-generated risk scores and observed mortality patterns was evaluated based upon the time-specific receiver operating characteristic (ROC) curve approach and definitions of incident sensitivity and dynamic specificity proposed by Heagerty and Zheng [58]. This evaluation method permitted calculation of a global concordance index (*C*), measuring overall correspondence between risk scores and observed mortality patterns, as well as time-specific metrics interpreted as ROC curve statistics, which provided a useful indication of the short versus long-term prognostic ability of a given model. For any survival time *t*, the framework proposed by Heagerty and Zheng [58] provides a measure of how well a risk score distinguishes at-risk subjects that die at time *t *from at-risk subjects that survive beyond time *t*. This is done by constructing time-specific ROC curves based upon estimates of incident sensitivity and dynamic specificity. Such a curve is generated from a risk score marker (*M*), by choosing different *M *threshold values (*c*) for predicting death, leading to a series of sensitivity and specificity estimates with respect to a given survival time *t *[58].

(1) $$\text{Sensitivity}\left(c,t\right)=P\left(M_{i}>c|T_{i}=t\right)$$

(2) $$\text{Specificity}\left(c,t\right)=P\left(M_{i}\leq c|T_{i}>t\right)$$

The estimated (incident) sensitivity rate forms the vertical axis of the time-specific ROC curve and reflects the ability of risk scores to accurately identify subjects that die at time *t *(i.e., true-positive identification rate). The estimated (dynamic) specificity rate is used to calculate false-positive rates, which forms the horizontal axis of the time-specific ROC curve, reflecting the degree to which high risk scores are incorrectly assigned to subjects living beyond time *t *(i.e., 1 - Specificity).

Time-specific ROC curves were used to calculate area under the curve statistics (i.e., AUC(*t*)), which provided intuitive and useful summary measures of prognostic capacity, with respect to certain survival times. The interpretation of AUC values is consistent with those generated in other contexts, with possible values ranging between 0 and 1, and values greater than 0.50 indicative of model output that is more useful than randomly generated risk scores. The value of AUC(*t*) can also be interpreted in probabilistic terms. For two subjects with risk scores *M_j _*and *M_k_*, the value of AUC(*t*) represents the probability that *M_j _*is larger than *M_k_*, given that subject *j *has a survival time of *t *and subject *k *has a longer survival time [58].

(3) $$AUC\left(t\right)=P\left(M_{j}>M_{k}|T_{j}=t,T_{k}>t\right)$$

The value of AUC(*t*) provides an indication of model discrimination ability at a given survival time. A global concordance measure (*C*) is obtained by integrating AUC(*t*) values with respect to *t*, providing an "averaged" value of AUC(*t*) that reflects overall discrimination ability across all survival times [58]. This concordance index is interpreted as the probability that the model can, for any two subjects chosen at random, correctly identify which subject was the first to die [58].

(4) $$C=P\left(M_{j}>M_{k}|T_{j}<T_{k}\right)$$

Values of AUC(*t*) and *C *are used in our analyses to evaluate the ability of models, developed on a randomly selected training set, to discriminate among the survival outcomes of subjects within a testing data set. In our analyses, data are randomly split 10,000 times into training and testing sets, and values of AUC(*t*) and *C *are estimated with each iteration. Discrimination ability of models over all iterations is measured by averaging values of AUC(*t*) and *C *across all 10,000 simulations.

## Results

**Figure 1:**
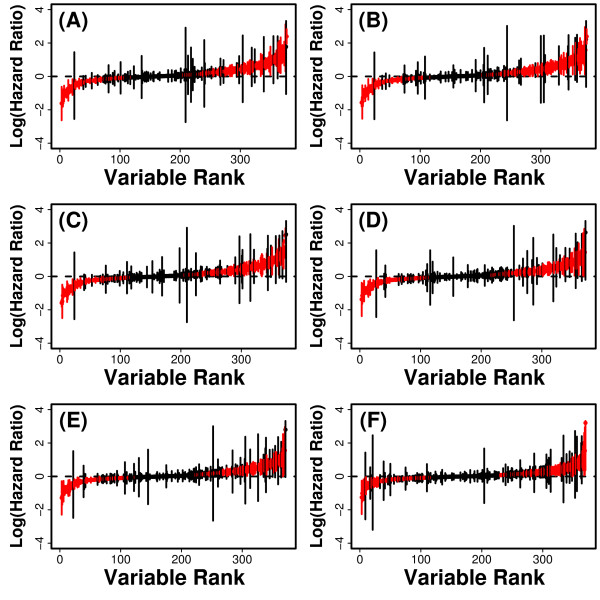
**Hazard ratios**. Cox PH models were used to calculate the hazard ratio associated with each of the 377 predictor variables. In each plot, vertical lines represent 95% confidence intervals associated with log-transformed hazard ratios, with red lines indicating statistical significance of estimated ratios (P < 0.05). Lines representing confidence intervals are ordered with respect to a variable ranking based upon the hazard ratio point estimate obtained for each variable. In part (A), hazard ratios for each predictor variable were estimated using a univariate model without other covariate terms. In parts (B) - (F), hazard ratios were adjusted for one or more covariates. These covariates include (B) baseline age, (C) age and smoking history, (D) age, smoking and diabetes history, (E) age, smoking history, diabetes history and hypertension, and (F) age, smoking history, diabetes history, hypertension and self-rated health.

Our analysis is based upon the youngest fraction of the SOF cohort, consisting of 4097 Caucasian women between 65 and 69 years of age (Additional File 1). Following baseline interviews and clinical examinations (September 1986 and October 1988), most women survived during as many as 20 years of follow-up, with 90% surviving at least 9.3 years or more, 80% surviving 13.7 years, 70% surviving 16.8 years, and 60% surviving 20.0 years (Kaplan-Meier estimates). At baseline, 3451 of the 4097 subjects (84.2%) indicated that, compared to others of the same age, their health was either "excellent" or "good", suggesting that most were in moderately good health at the beginning of the study. We evaluated 377 predictor variables generated from information collected at the baseline evaluation, and found that many of these variables were significantly associated with survival. We first analyzed each variable individually by fitting a univariate Cox PH model. Most variables (213/377) were significantly associated with survival (P < 0.05), and for 38 variables, p-values were not distinguishable from zero. We considered whether this large number of significant results was due to correlations between predictors and certain variables expected to have strong associations with survival, such as baseline age, smoking history (i.e., whether a subject had smoked at least 100 cigarettes in her lifetime), or whether a subject had ever been told by a doctor that she was diabetic (Figure 1). This was not the case, however, and we found that 214 variables were significant after adjusting for patient age, 212 variables were significant after adjusting for both age and smoking history, while 201 variables were significant after adjusting for age, smoking history and diabetes history (Figure 1A - 1D). We next evaluated the significance of variables after adjusting for more direct indicators of baseline health, such as presence of hypertension and a subject's self-rated health compared to others of the same age. After adjusting for age, smoking history, diabetes history and hypertension, 190 of the 377 variables were significant (Figure 1E). Likewise, a large number of variables (168/377) were significant following adjustment for age, smoking history, diabetes history, hypertension and self-rated health status (Figure 1F).

### Best Univariate Predictors of Long-term Survival

**Figure 2:**
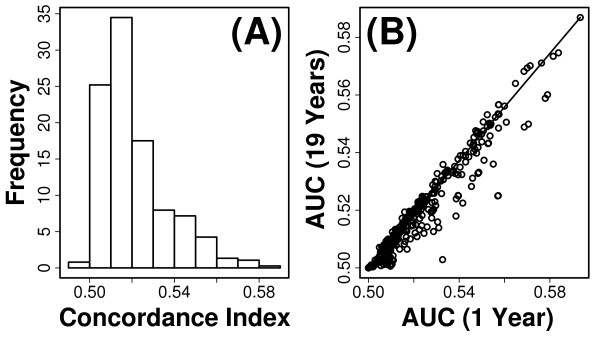
**Cross-validation performance of univariate models**. The predictive value of each variable was evaluated based upon 10,000 cross-validation trials. In each trial, a univariate Cox PH model was fit using a randomly chosen training dataset, and model performance was evaluated by applying the fitted model to subjects within a randomly chosen testing dataset. The predictive value of each variable was measured according to the average concordance index (*C*) among simulation trials (see Methods). The concordance index is an average of time-specific area under the curve statistics (i.e., AUC(*t*)), which were calculated for *t *= 1, ..., 19 years of follow-up time (see Methods). Part (A) shows the distribution of mean concordance estimates among all 377 predictor variables. In part (B), for each variable, the average value of AUC(19) among simulation trials is plotted against the average value of AUC(1).

The prognostic capacity of each predictor was evaluated using 10,000 cross-validation trials, with discrimination ability of each model measured by area under the curve (AUC) statistics generated from time-specific receiver operating characteristic (ROC) curves [58] (see Methods). The overall quality of a model, with respect to all survival times, was measured by a concordance index (*C*), which corresponds to an average of AUC(*t*) values across all survival times [58] (see Methods). Among the 377 variables, the mean value of *C *across simulations ranged from 0.500 to 0.589, and for most variables, the average value of *C *was less than 0.550 (Figure 2A). The prognostic capacity of variables generally declined over time, although in most cases this decline was slight (Figure 2B). For instance, there were 311 variables for which the mean value of AUC(1) (AUC for survival of one year) across simulations was larger than that of AUC(19) (corresponding to survival to 19 years), but the opposite was true for just 66 variables (Figure 2B).

**Figure 3:**
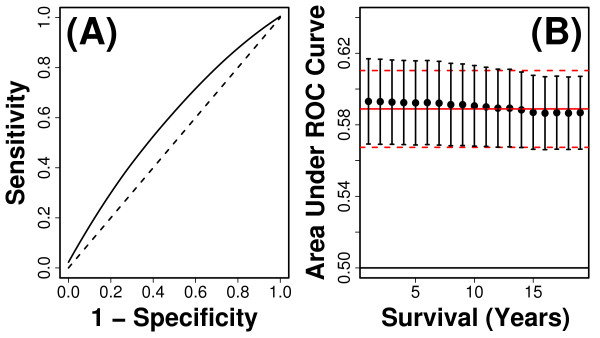
**Number of step-ups completed in 10 seconds**. The number of step-ups completed in 10 seconds was the best single-variable predictor of short and long-term survival (mean *C *= 0.589). Part (A) shows the time-specific ROC curve corresponding to 10-years of follow-up time. Estimates of sensitivity and specificity were calculated as described in Methods and averaged among simulation trials. The dashed line corresponds to the null expectation with an AUC value of 0.50. Part (B) plots estimated values of AUC(*t*) across *t *= 1,..., 19 years of follow-up time. Each point represents the average value of AUC(*t*) among 10,000 simulation trials, and error bars indicate the standard deviation of AUC(*t*) among simulations. The solid red line indicates the mean concordance index of 0.589 among simulations, and dashed red lines represent one standard deviation above and below the mean concordance estimate.

The strongest predictors appeared to fall into three general categories: (i) physical performance measures, (ii) contrast sensitivity scores from a visual exam, and (iii) self-rated health assessments based on a self-administered questionnaire (Table 1). Physical performance measures represented the most useful predictors of survival, and included the number of step-ups a subject was able to complete within 10 seconds (mean *C *= 0.589), average step length (mean *C *= 0.577) and walking speed (mean *C *= 0.573). The number of step-ups completed in 10 seconds was, among all variables, the strongest predictor of survival (Figure 3A). The predictive value of this variable did decline over time, but this decline was relatively small (Figure 3B), and this same variable also had the largest value of AUC(19) among all variables (AUC(19) = 0.587). Contrast sensitivity refers to the ability of a subject to detect differences between grey objects that vary in color between white and black (measured using Pelli-Robson letter charts [59]). We found that four related measures of contrast sensitivity emerged as particularly useful predictors of survival (0.564 ≤ average *C *≤ 0.570) (Table 1). These measures differ in terms of whether scores reflect both low and high spatial frequency versus low spatial frequency, or whether scores were normalized to the entire population of subjects, but generally the four scores were correlated with one another and associated with similar concordance values. Lastly, the best predictors of survival included variables derived from self-administered questionnaires completed by subjects (Table 1). These measures were a subject's rating of their own health compared to others (mean *C *= 0.579), along with composite functional status indices measuring how well subjects could perform daily living tasks, such as walking, climbing up stairs, climbing down stairs, preparing meals, doing housework, and shopping (mean *C *= 0.569 for both the 6 and 5-variable indices).

**Table 1:** Variables with greatest predictive value in univariate Cox PH models

Variable	Mean Concordance (*C*)
Number of step-ups completed in 10 seconds	0.589

Response to Question: How is your health compared to others your age? (categories: excellent, good, fair, poor, very poor)	0.579

Average step length, Usual Pace (m)	0.577

Walking speed, Usual Pace (m/s)	0.573

Contrast sensitivity score, average of high and low spatial frequencies	0.570

Contrast sensitivity score, average of low spatial frequencies	0.569

Impairments associated with daily living, index of 6 tasks	0.569

Contrast sensitivity score, average of low spatial frequencies, normalized	0.568

Impairments associated with daily living, index of 5 tasks (walking 2 or 3 blocks, climbing up 10 steps, climbing down 10 steps, preparing own means and doing housework).	0.568

Contrast sensitivity score, average of low and high spatial frequencies, normalized	0.564

The first column provides a brief description of each variable. Mean concordance refers to an average of concordance index (*C*) estimates calculated in each of 10,000 cross-validation simulation trials (see Methods). The 95% confidence interval associated with the mean concordance estimate for each variable is approximately ± 0.001.

**Figure 4:**
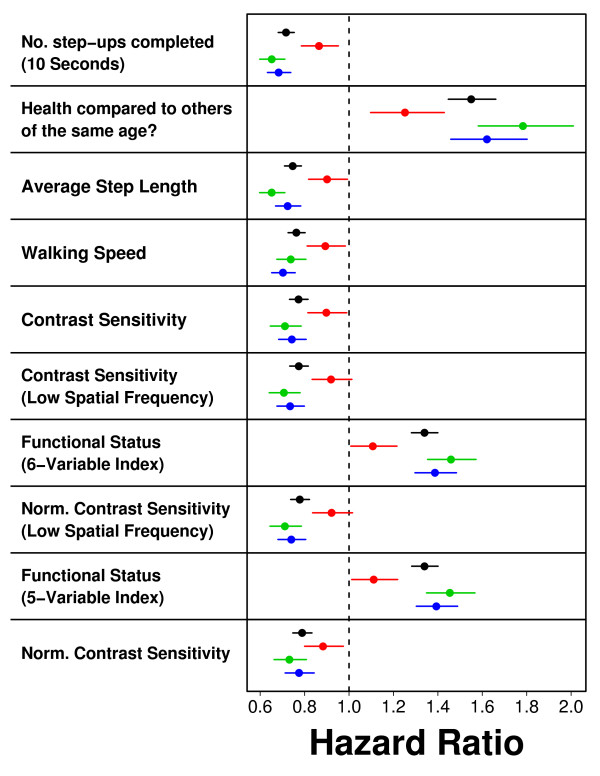
**Hazard ratio estimates associated with best predictor variables**. The hazard ratio was estimated using single-variable Cox PH models for each of the best predictor variables listed in Table 1. Each point represents a hazard ratio estimate, and horizontal lines correspond to 95% confidence intervals associated with this estimated value. Black points correspond to hazard ratios estimated using data from all *n *= 4097 subjects. Hazard ratios were separately estimated based upon the *n *= 467 cancer deaths (red points), the *n *= 426 cardiovascular deaths (green points), and the *n *= 586 non-accidental/non-cancer/non-cardiovascular deaths (blue points). Cause-specific ratios were calculated by treating the *n *deaths assigned to a specific cause as non-censored, with censoring applied to deaths assigned to any other cause.

The variables listed in Table 1 had a relationship to mortality that was roughly consistent among subjects assigned different causes of death. For each measure, we calculated hazard ratios with respect to the entire cohort (*n *= 4097), or in subsets of subjects that appeared to die of cancer (*n *= 426), of cardiovascular disease (*n *= 467), or of non-accidental/non-cancer/non-cardiovascular disease (*n *= 586). With respect to each subset, variables listed in Table 1 had a similar association with mortality, although the association was in each case weaker among subjects for which the assigned cause of death was cancer (Figure 4).

We considered whether the associations between Table 1 variables and survival might be indirect, and due primarily to correlations with, for example, smoking or diabetes history. We thus evaluated whether hazard ratio estimates of Table 1 variables remained significant in bivariate Cox PH models that included a second measure of potential importance. For each Table 1 variable, we estimated a hazard ratio in 376 separate bivariate models, representing all possible bivariate models that include the Table 1 variable, given the total number of variables included in the analysis (i.e., 377 variables). For each Table 1 variable, significance of the estimated hazard ratio was not altered within any bivariate Cox PH model, with only two exceptions. First, contrast sensitivity measures became non-significant (P > 0.05) if another contrast sensitivity measure was included in the same model, and second, the 5- and 6-variable functional status indices were non-significant if both were included within the same model. Otherwise, each variable shown in Table 1 remained significant after adjustment for any of the other variables included in the analysis, including smoking history, diabetes history and baseline age.

### Data mining strategy for building a predictive model

**Figure 5:**
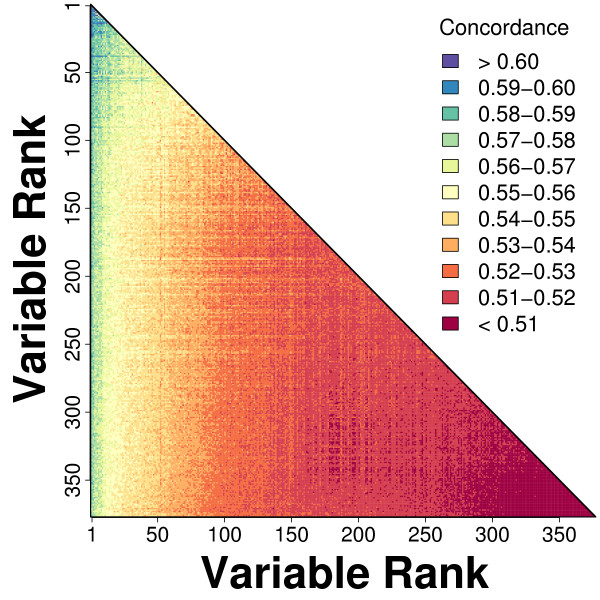
**Performance of bivariate models**. For each of _377_C_2 _= 70876 possible combinations of the 377 predictor variables, we generated bivariate Cox PH models and evaluated the average concordance index (*C*) of each model across 20 cross-validation simulations. Colors represent the average concordance of a given model, with respect to the ranking of the two variables contained in a model. The variable rankings are based upon concordance estimates generated for each variable in univariate Cox PH models (i.e., values from Figure 2A), with low ranks assigned to variables of greatest predictive value.

We next searched for favorable combinations of variables that best predicted long-term survival. Among the 377 variables, there were _377_C_2 _= 70876 possible pairwise combinations of variables. For each of the 70876 combinations, we constructed bivariate Cox PH models and evaluated their discrimination ability using the same cross-validation procedure described above, except that only 20 simulation trials were performed for each bivariate model. We calculated the average concordance among the 20 trials, and this revealed that the best models generally consisted of variables that, in univariate models, had been ranked among the top 50 strongest predictors (Figure 5). Using these results as a rough screen, we identified the best 200 bivariate models, and for these top 200 models, we performed 10,000 cross-validation simulations, and calculated the average value of *C *among the 10,000 trials. This indicated that the best 2-variable model was based upon the number of step-ups completed by a subject in 10 seconds, in combination with information related to smoking history. Specifically, the best model included the number of step-ups completed and an indicator variable with value of 1 if a subject had previously smoked (average *C *= 0.614). The next two top models were also based upon the number of step-ups completed, and differed only slightly, with the second variable related to smoking history in each case (average *C *= 0.613).

**Figure 6:**
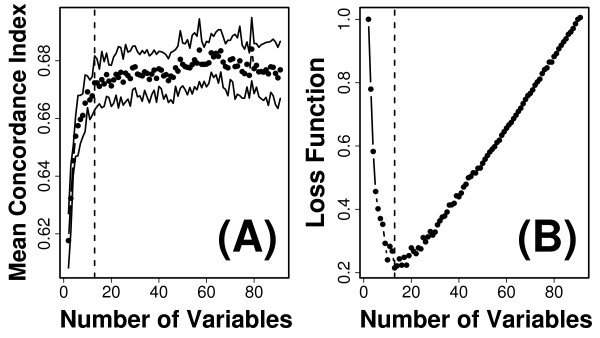
**Forward variable selection**. The best bivariate model was based upon the number of step-ups completed by a subject in 10 seconds and whether a subject previously smoked (average *C *= 0.614). Variables were iteratively added to this model to evaluate concordance values associated with larger models. At each iteration, given a baseline model with *p *variables, the concordance associated with all possible models containing *p *+ 1 variables was evaluated (based upon 20 cross-validation simulations). The best model containing *p *+ 1 variables was chosen as a new baseline model and the process repeated. Points in part (A) show the mean concordance index associated with each of the models created by this process, and upper and lower lines indicate 95% confidence limits. (B) The size of a tentative model was chosen based upon the value of *p *that minimized a loss function. The point of diminishing returns with increasing *p *corresponds to a "knee" or leveling off point of the curve shown in part (A). To quantitatively locate this point, the scales shown in part (A) were mapped to the interval [0,1], and a loss function was defined as the distance between the plotted curve and the extreme upper-left corner of the coordinate system. The value of *p *that minimized this loss function is denoted by the dashed vertical lines in parts (A) and (B) (i.e., *p *= 13 variables).

**Figure 7:**
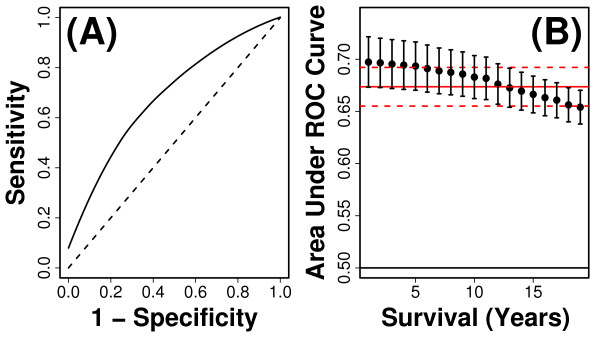
**Cross-validation performance of 13-variable index**. The multivariate index we developed is based upon a model that includes 13 variables (see Table 2). The discrimination ability of risk scores generated from this model was evaluated based upon cross-validation methods and time-specific ROC curve metrics (e.g., see Figure 3). In each of 10,000 simulations, risk scores were calculated as linear predictors of Cox regression models that included the 13 variables listed in Table 2, with coefficients estimated using 3687 subjects randomly assigned to the testing set in each simulation, and prediction accuracy evaluated based upon discrimination ability of risk scores with respect to 410 subjects randomly assigned to the testing set in each simulation (see Methods). In part (A), the estimated time-specific ROC curve corresponding to 10-years follow-up time is shown. The dashed line corresponds to the null expectation with an AUC value of 0.50. Part (B) plots estimated values of AUC(*t*) across *t *= 1,..., 19 years of follow-up time. Each point represents the average value of AUC(*t*) among 10,000 simulation trials, and error bars indicate the standard deviation of AUC(*t*) among simulations. The solid red line indicates the mean concordance index of 0.671 among simulations, and dashed red lines represent one standard deviation above and below the mean concordance estimate.

Forward variable selection was then used to identify a model that incorporated a larger number of predictor variables. In this approach, given a "best" model with *p *variables, we screened all possible models with *p *+ 1 variables that could be formed by adding a new variable to the best *p*-variable model. The process was initiated using the best bivariate model (identified above; average *C *= 0.614), and iteratively repeated until the performance of very large *p*-variable models had been evaluated (Figure 6). At each iteration, possible models were evaluated using 20 cross-validation simulations, and for each possible model, the average value of *C *was calculated and used to determine which model had the best performance. We found, as expected, that the generation of larger models through addition of new predictor variables quickly reached a point of diminishing returns (Figure 6A). The appropriate level of model complexity (i.e., number of variables) corresponds to the "bend" in the curve that is shown in Figure 6A. To identify this point quantitatively, we defined a loss function equal to the distance between the curve shown in Figure 6A and the upper-left hand corner of the plotting region. This criterion suggested that the forward selection strategy should be halted after a model with 13 variables had been generated (see Figure 6B). We thus repeated the forward selection process, and at each iteration, alternative models were evaluated using 500 cross-validation simulations, and the best ten models were then re-evaluated at each iteration using 20,000 cross-validation simulations. This yielded a 13-variable model with an average concordance value of 0.673 (Figure 7).

We searched for less complex models that might have performed equally well, by dropping each of the 13 terms one-at-a-time, and evaluating the mean concordance associated with each resulting 12-variable model. However, we were unable to identify a smaller model that yielded a mean concordance value equal to or greater than 0.673. Overall, based on the process described above, the 13-variable model we identified is likely to be a high-quality model, but our partly heuristic variable selection process does not exclude the possibility that another of 4.05 × 10^23 ^possible 13-variable models might exhibit better performance. Lastly, we note that AUC values associated with our model are small (Figure 7) relative to some that have been reported in the literature for prediction of survival [60]. In part, this may reflect characteristics of the cohort we evaluated, which did not include shorter-lived older subjects for which it is usually easier to generate accurate mortality forecasts (e.g., see Figure 2B). Additionally, AUC values reported in our study may differ from those of some previous investigations due to differences in overall cohort size, the set of predictor variables used to build prognostic models, the type of cross-validation procedure implemented (e.g., ten-fold, five-fold, leave-one-out, etc.), or the method used to calculate AUC values.

### A 13-variable Index that Predicts Long-Term Survival in Women 65-69 Years of Age

The composition of the 13-variable model is described in Table 2. Collectively, the variables relate to several dimensions of an aging adult, including a measure of physical performance (i.e., number of step-ups completed in 10 seconds), smoking and diabetes history, age at baseline, self-evaluation of health compared to others, a measure of visual performance (i.e., contrast sensitivity), indicators of cardiovascular health (i.e., pulse, hypertension/thiazide use), height change since age 25, and variables relating to environmental factors (i.e., the clinic attended throughout the study and marriage status) (Table 2). Some of the identified variables are probable health determinants (e.g., smoking), while others are more likely indicators of overall health and may not have a causal association with survival (e.g., number of step-ups completed in 10 seconds). The estimated hazard ratio for each of the 13 variables was statistically significant (P < 0.01), even when adjusted for all other variables included in the model (Table 2). Of the 13 variables, nine could be scored based upon a self-administered questionnaire, and only four variables require physical examination (number of step-ups completed in 10 seconds, contrast sensitivity, pulse, hypertension/thiazide use). For variables requiring a physical exam, we have listed alternative measures that could be easier to evaluate in clinical settings (Table 2). In most cases, the use of one substitute variable did not greatly diminish the average concordance index of the complete model (Table 2).

**Table 2:** Multivariate index for prediction of long-term survival

Variable	HR (P-Value)
Number of step-ups completed in 10 seconds^1^	0.832 (< 0.001)

Smoking: indicator with value 1 if subject is a current smoker	2.354 (< 0.001)

Diabetes: indicator with value 1 if a subject is not diabetic	0.443 (< 0.001)

Age at baseline examination (65 - 69 for all subjects)	1.146 (< 0.001)

Response to Question: How is your health compared to others your age? (categories: excellent, good, fair, poor, very poor)	1.205 (< 0.001)

Smoking: indicator with value 1 if subject is a past smoker	1.390 (< 0.001)

Contrast sensitivity score, average of high and low spatial frequencies^2^	0.879 (< 0.001)

Pulse Lying Down (beats/60 seconds)^3^	1.131 (< 0.001)

Hypertension: indicator with value 1 if systolic blood pressure exceeds 160, diastolic blood pressure exceeds 90, or if subject used thiazide^4^	1.324 (< 0.001)

Past thiazide use: indicator variable with value 1 if the subject has previously used thiazide	1.785 (< 0.001)

Height change since the age of 25 (self-reported at baseline exam)	1.137 (< 0.001)

Participant's clinic throughout the study: indicator with value 1 if subject has attended clinic B	1.357 (< 0.001)

Marriage: indicator with value 1 if subject was married at the time of the baseline examination	0.822 (< 0.001)


**Figure 8:**
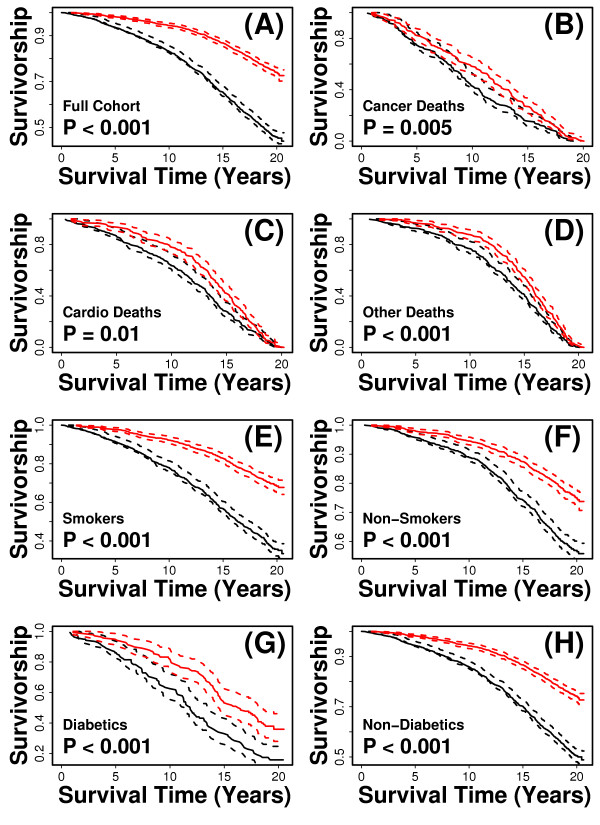
**The 13-variable index distinguishes between short and long-lived subjects with respect to multiple sub-cohorts**. The index was used to assign a risk score to each subject using leave-one-out cross validation. In this method, a score is assigned to each of the *n *subjects, based upon a model fit to data from the other *n *- 1 subjects included in the dataset. After a score had been assigned to all subjects, the median score among all subjects was calculated. Subjects were assigned to a "low-risk group" if their score was below the median value, and were assigned to a "high-risk group" if their score was above the median value. The solid red line corresponds to the estimated Kaplan-Meier survival curve for the low-risk group, and the solid black line corresponds to the estimated curve for the high-risk group. Dotted red and black lines represent 95% confidence limits. In part (A), survival curves were generated from all *n *= 4097 subjects. In parts (B) - (H), the analysis was performed with respect to certain sub-populations of subjects. These sub-populations include (B) the *n *= 426 subjects dying of cancer, (C) the *n *= 467 subjects with cardiovascular-related deaths, (D) the *n *= 586 subjects with non-accidental deaths unrelated to cancer or cardiovascular disease, (E) the *n *= 1776 past or present smokers, (F) the *n *= 2316 non-smokers, (G) the *n *= 274 diabetic subjects, and (H) the *n *= 3823 non-diabetic subjects. P-values were generated from a log-rank test of the null hypothesis that low and high-risk groups have identical Kaplan-Meier survival curves [116].

**Figure 9:**
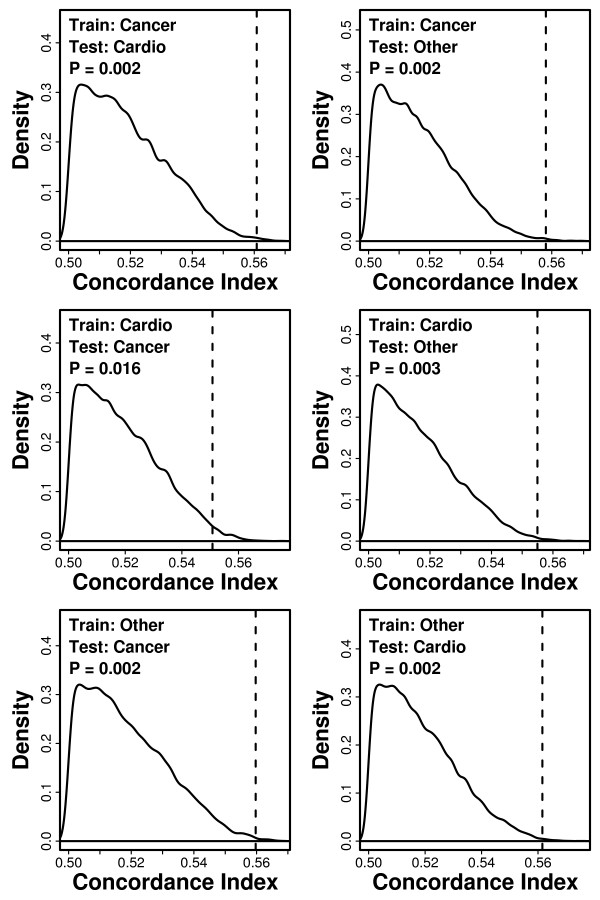
**Cross-validation by cause of death**. Index coefficients were estimated based upon a training set of subjects that had died from one specific cause (cancer, cardiovascular, or non-cancer/non-cardiovascular). This generated a fitted model that was applied to a test set of subjects that had died from another specific cause, which differed from that of subjects in the training set. The discrimination ability of the model with respect to the survival times of subjects belonging to the test set was measured by estimating the concordance index (*C*). Train and test sets were either the *n *= 426 subjects that died of cancer, the *n *= 467 with cardiovascular-related deaths, or the *n *= 586 subjects with non-accidental deaths unrelated to cancer or cardiovascular disease. The dotted vertical line represents the estimated concordance index obtained for each cross-validation scenario. The density shown corresponds to a null distribution generated by 10,000 simulation trials. In these simulations, survival times in the training set were randomly permuted among subjects prior to estimation of model coefficients. The null distribution thus provides an indication of concordance values likely to arise by chance.

**Figure 10:**
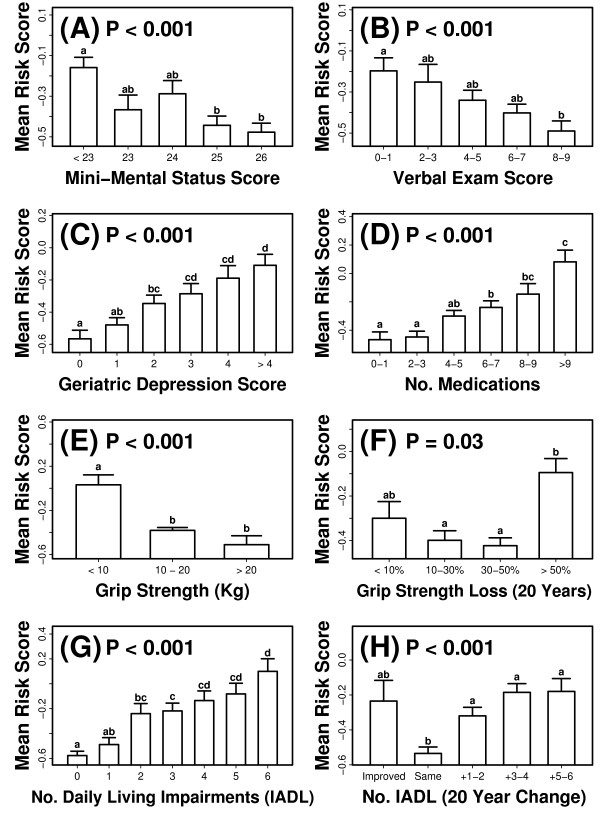
**Risk scores generated from the 13-variable index are associated with multiple outcomes among subjects surviving the follow-up period (mean follow-up time: 20 years)**. Risk scores were calculated from baseline measures for all 4097 subjects and standardized to have a mean of zero and standard deviation of one. Outcome measures were available for *n *= 923 to 1296 subjects, and included (A) Mini-mental status exam score (0 - 26 point scale; *n *= 98 - 257 per group), (B) California Verbal Learning Test (10 minute delay, free recall; 0 - 9 point scale; *n *= 91 - 275 per group), (C) Geriatric depression score (0 - 15 point scale; n = 79 - 225 per group), (D) total number of prescription medications listed by each subject (*n *= 111 - 336 per group), (E) average of right and left hand grip strength (kg) (*n *= 79 - 763 per group), (F) change in average grip strength between the first and ninth visits (*n *= 79 - 389 per group), (G) reported number of impairments associated with daily living (*n *= 84 - 359 per group) and (H) the difference in reported number of daily living impairments between the first and ninth visits (*n *= 41 - 357 per group). The p-value listed in each panel was generated from an F-test of between-group differences in mean risk score (i.e., one way analysis of variance). Lowercase letters above each bar correspond to results from *post hoc *Tukey-Kramer comparisons, with significant differences between groups that do not share the same letter (P < 0.05).

The table lists 13 variables that comprise a subset of variables that predict long-term survival as components of a Cox PH model (mean *C *= 0.673 ± 0.001). The 13 variables were identified through forward search (see Results and Figure 6), and are listed in the order they were introduced into the model during the forward selection procedure (i.e., from most to least important). The second column lists the estimated hazard ratio (HR) and p-value, based upon coefficients obtained from the 13-variable Cox PH model. For variables that require physical measurement in a clinical setting, footnotes list potential alternative variables, along with the average concordance index of an index that includes the alternate variable rather than the variable listed in Table 2. The listed variables were used to generate the risk scores evaluated in Figures 8, 9 and 10, where risk scores for test subjects represent the output of a 13-variable Cox model with coefficients corresponding to measures listed in the table below.

^1^Alternative variables: walking speed (m/sec) (mean *C *= 0.671); average step length at usual pace (m) (mean *C *= 0.671); average of right and left hand grip strength (kg) (mean *C *= 0.668); 12-variable model with variable omitted (mean *C *= 0.668).

^2^Alternative variables: indicator with value 1 if subject does not wear glasses (mean *C *= 0.671); Randot test of stereoacuity, 10-level test scored based upon the highest level correct twice (mean *C *= 0.671); 12-variable model with variable omitted (mean *C *= 0.671).

^3^Alternative variables: pulse while standing (beats/60 seconds) (mean *C *= 0.672); diastolic blood pressure while standing (mm hg) (mean *C *= 0.671); systolic blood pressure while standing (mean *C *= 0.671); 12-variable model with variable omitted (mean *C *= 0.671).

^4^Alternative variables: waist-to-hip ratio (mean *C *= 0.671); body mass index (mean *C *= 0.670); 12-variable model with variable omitted (mean *C *= 0.670).

The 13 variables did not include biochemical measures, but we evaluated whether risk scores generated from the model were associated with blood serum measures obtained from a small SOF cohort during the baseline visit (*n *≤ 400 for each of 37 measures considered). We found that model risk scores were positively associated with C-reactive protein (*r_s _*= 0.20; P = 0.013, *n *= 151), chorionic gonadotropin (*r_s _*= 0.18; P = 0.026; *n *= 150), and 1,25-hydroxyvitamin D (*r_s _*= 0.16; P = 0.048; *n *= 151), but negatively associated with 25-hydroxyvitamin D (*r_s _*= -0.21; P = 0.010; *n *= 152). In each case, the observed correlations, though significant, were modest in magnitude (|*r*| ≤ 0.21.

We evaluated whether variables included in our model were age-sensitive and whether the prognostic value of the model and component variables extended to older subjects in the SOF cohort (i.e., 70 - 89 years of age; Additional Files 3 and 4). First, to evaluate the age-sensitivity of variables within the model, we carried out a cross-sectional analysis based upon all 9704 subjects within the SOF cohort (i.e., ages 65 - 89; Additional File 3). This analysis demonstrated that certain continuous variables within the model exhibited monotonic or near-monotonic trends across age groups (e.g., contrast sensitivity scores, number of step-ups completed in 10 seconds, pulse lying down, self-reported height loss since age 25; see Additional File 3), although we note that inferences concerning age-associated trends based upon cross-sectional data should be treated with caution. We next used cross-validation methods to determine whether the model accurately forecasted survival patterns among the older SOF subjects excluded from the above analyses (i.e., subjects 70 - 89 years of age; Additional File 4). Among older SOF subjects, the model predicted survival patterns with greater accuracy than expected on the basis of chance alone, with a mean concordance estimates of 0.630 among those aged 70-74 (*n *= 3033), 0.616 among those aged 75-79 (*n *= 1538), 0.586 among those aged 80-84 (*n *= 765), and 0.584 among those aged 85-89 (*n *= 228) (see Additional File 4; each estimate based on 10,000 cross-validation simulations). The progressive decline of predictive performance among increasingly older SOF cohorts may be attributed to the fact that the index was developed for prediction of (long-term) survival with respect to a relatively young cohort (age 65-69), and thus includes variables for which prognostic value declines with age (i.e., smoking and diabetes status at baseline; see Figures C and D from Additional File 4).

Previous studies have shown that random choice of variables can often provide high-quality models that predict short or long-term survival, suggesting that prediction accuracy may in some cases not be sensitive to an exact specification of model variables [40,61]. To evaluate this possibility, we compared the performance of our 13-varible model to models formed by choosing variables randomly from the 377 variables evaluated on the cohort we studied (Additional File 5). This showed that random selection of variables can indeed lead to models that perform moderately well (0.583 ≤ mean *C *≤ 0.633, depending upon how the pool of eligible variables is filtered), but in each case performance was poorer and less consistent relative to that of the 13-variable model described in Table 2 (see Additional File 5). We note, however, that our model includes only 13 variables, and that exact variable specification is likely to be less important for larger models that include more variables [62]. Lastly, we compared performance of our 13-variable model to that of models in which an index quantifying accumulation of many deficits served as the predictor variable [22] (Additional File 6). This analysis showed that a deficit index generated survival forecasts more accurate than expected on the basis of chance alone (0.584 < mean *C *< 0.630) (Additional File 6, Figures C - F). Among the youngest SOF subjects (age 65-69), our 13-variable index outperformed the deficit index (mean *C *of 0.673 versus 0.617), but this advantage progressively declined as we examined increasingly older SOF cohorts (Additional File 6, Figures C - F).

### Evaluation of the Index as a Measure of "Healthy Aging"

An index that characterizes "healthy aging" should not primarily reflect a single major disease process, but should instead be sensitive to multiple forms of age-related disease, and should indeed predict multiple age-associated outcomes besides survival alone. The index developed above was thus further evaluated along these lines in order to judge its value as a comprehensive measure of healthy aging. We first evaluated whether the index was able to characterize multiple forms of age-related disease, which appeared plausible, since some variables included in the index (e.g., number of step-ups completed and contrast sensitivity) had a similar association with mortality regardless of the assigned cause of death (see Figure 4). We therefore hypothesized that model output would be informative with respect to sub-populations of subjects that appeared to develop different types of age-related pathology.

A risk score was assigned to each subject based upon the 13-variable index, such that for each subject, the assigned score was generated from an index in which coefficients were estimated using data from all other subjects (i.e., leave-one-out cross-validation). After scores had been assigned in this fashion, subjects were divided into two equally sized groups, based upon whether assigned scores were above or below the cohort median, and we evaluated the survivorship patterns of the two groups (Figure 8). Initially, we applied this analysis to the entire cohort, and as expected, found that subjects assigned low risk scores survived significantly longer than those assigned high risk scores (Figure 8A; P < 0.001; log-rank test). We then repeated the procedure, but limited the analysis to subjects for which the assigned cause of death was cancer (*n *= 426), cardiovascular disease (*n *= 467), or other non-accidental causes of death (*n *= 586). In each case, scores generated from the 13-variable index led to subject groupings with significantly different survival patterns (P < 0.021; log-rank test) (Figures 8B - D). We next considered whether the index primarily characterized deleterious health effects of smoking or diabetes, given that these factors are associated with multiple forms of age-related illness. This did not appear to be the case, however, because as above, we found that for self-identified past or current smokers (*n *= 1776), self-identified non-smokers (*n *= 2316), subjects reporting that they had been told by a doctor they were diabetic (*n *= 274) and apparent non-diabetics (*n *= 3823), index-generated risk scores led to subject groupings with significantly different survival patterns (P < 0.001; log-rank test) (Figures 8E - H). Taken together, these analyses confirmed that the index characterizes mortality among subjects with multiple assigned causes of death, and show that the index is not primarily a reflection of a subject's smoking or diabetes status.

The value of the index as an indicator of multiple forms of age-related illness was further evaluated using a second analytical strategy. In particular, we evaluated whether data patterns associated with subjects developing one type of pathology could be used to predict survivorship among subjects developing an entirely different type of pathology. To apply such a test, we focused on three groups of subjects, for which physician-adjudicated cause of death was cancer (*n *= 426), cardiovascular disease (*n *= 467), or other non-accidental/non-cancer/non-cardiovascular disease (*n *= 586), respectively. We estimated coefficients of the 13-variable model using data from one group of subjects dying of one specific cause (the training set), and then used these coefficients to generate risk scores for another group of subjects dying from a different cause (the test set) (Figure 9). Among the three groups of subjects, there were six possible training-test set pairings, and in each case, we evaluated whether risk scores generated in this fashion led to concordance estimates larger than expected by chance (Figure 9). In each of the six scenarios, we found that risk scores led to significant concordance estimates (P < 0.016). For example, when index coefficients were estimated using data from subjects apparently dying of cancer, this index was applied to generate risk scores for subjects that appeared to die of cardiovascular disease, and the concordance between risk scores and survival times in this latter group was 0.562, which is much larger than expected by chance (P = 0.002; see Figure 9). These results suggest that the model's prognostic value arises from characterization of processes more general than any one type of disease, and support the hypothesis that the model provides a characterization of healthy aging.

The index predicted mortality patterns, regardless of the assigned cause of death, but only a fraction of the 4097 women included in our analysis died during the period of follow-up (1523 of 4097; 37.2%). It was therefore of interest to determine whether risk scores generated from the index were related to long-term outcomes among subjects that survived the follow-up period. For a subset of the surviving subjects (923 ≤ *n *≤ 1315), several outcome measures had been evaluated at the ninth SOF visit (occurring between 12/2006 and 7/2008; mean follow-up time: 20 years), and we therefore could evaluate whether these outcomes were associated with index-generated risk scores. For each of the 4097 subjects, a risk score was generated based upon an index with coefficients estimated using data from all other subjects (i.e., leave-one-out cross-validation; see above), and these scores were then standardized with respect to the baseline cohort of 4097 women (i.e., mean = 0, std. dev. = 1). These standardized risk scores were, as expected, lower on average among subjects that survived the follow-up period (mean score: - 0.26 ± 0.016), relative to those that died (0.46 ± 0.028). With respect to survivors, however, risk scores were associated with cognitive outcomes and measures of overall health (Figure 10). Lower risk scores were, on average, characteristic of subjects with higher scores on tests of cognitive function (Short-mini-mental status exam and California Verbal learning test; Figures 10A and 10B), subjects with lower scores on measures of geriatric depression (Geriatric Depression Scale; Figure 10), and subjects that reported a smaller number of prescription medications (Figure 10D). Risk scores were also associated with physical performance measures at the ninth SOF visit, as well as the change in physical performance between the first and ninth visits. For example, at the ninth visit, risk scores were lower among subjects with higher grip strength (Figure 10E), and between visits one and nine, the decline in grip strength was larger on average among subjects assigned higher risk scores (Figure 10F). A similar trend was present with respect to the number of impairments associated with daily living (six-variable index). Risk scores were lower among subjects that reported fewer daily living impairments at the ninth visit (Figure 10G), and were also lower among subjects for which the reported number of daily living impairments remained constant between the first and ninth visit (Figure 10H). These results demonstrate that, apart from mortality patterns, the 13-variable index had a long-term association with measures of cognitive function, mental outlook, indicators of overall health status, and measures of physical performance.

## Discussion

The prospects of long-term survival and successful aging can be evaluated in healthy adults based upon characteristics that reflect an individual's aging trajectory. In clinical settings, these characteristics can identify those at greatest risk of developing age-related disease, at a time prior to disease onset, when preventative measures can still be implemented effectively [3]. Moreover, for research purposes, such characteristics provide useful standards for the evaluation of human anti-aging interventions [4]. This study has identified individual factors that are most strongly associated with long-term survival within a healthy cohort of older women between 65 and 69 years of age. Surprisingly, visual contrast sensitivity was among the top 5 strongest predictors of survival relative to all 377 phenotypic measures evaluated in our study (mean AUC = 0.570) (Table 1). This measure may warrant increased attention in clinical evaluation, since our findings indicate that its prognostic significance is comparable to that of smoking and diabetes status. Our study has also derived an evidence-based index that ties together multiple dimensions of an aging adult (mean AUC = 0.673). This index is based upon the number of step-ups completed in 10 seconds, contrast sensitivity, blood pressure, pulse and several pieces of information easily obtained from a questionnaire or brief interview (Table 2). The prognostic capacity of this index did not appear to depend upon characterization of any one disease process, and among surviving subjects, scores generated from the index were associated with multiple long-term outcomes (e.g., mini-mental status exam score). These properties require further validation in independent cohorts, but suggest that the index could provide a marker of healthy aging patterns in older women (65-69 years of age), and that age-sensitive components of this index should be considered for possible use as endpoints for research centered on anti-aging interventions in humans.

The analytic approach used in our study differs in two main ways from previous investigations of data generated from the study of osteoporotic fractures (SOF). First, we have focused on the youngest subjects that enrolled in the study (i.e., ages 65 - 69 at baseline), while prior investigations have based their analyses on the complete SOF cohort (ages 65 - 89 at baseline). We have chosen to consider only younger subjects, since in these individuals, the burden of age-related disease was reduced at the time of baseline examination. This was desirable, since we expected that those already affected by age-related disease would exhibit a distinct signature set of phenotypic characteristics (i.e., a "frail" phenoytpe), and that this signal in the data would interfere with our ability to identify patterns associated with long-term survival, which we expected to be more informative in terms of aging mechanisms [25]. Secondly, prior investigations have aimed to identify single factors associated with survival, and to determine which factors were independent predictors of survival after adjusting for other influential variables. Along these lines, previous analyses have identified many variables associated with all-cause mortality in the SOF cohort, including fracture incidence and rate of bone loss [63-68], markers of cardiovascular health and function [69,70], biochemical measures [71-74], body composition traits [30,75], physical activity [76], sleeping habits [77], depressive symptoms [48], marital status and social connectedness [49], as well as visual acuity and contrast sensitivity [38]. In our investigation, we also identified statistically significant predictors of survival, which remained significant after adjustment for other variables. However, our approach was more stringent in some respects, since we aimed to identify variables that were both significantly related to survival and also the strongest predictors *relative *to other baseline variables evaluated in the SOF cohort. These variables were isolated by adopting a global data mining strategy that involved competition among a wide range of variables, and variable combinations, allowing the most useful variables to emerge in a data-driven fashion.

Visual contrast sensitivity was a strong independent predictor of survival as well as an influential component of the multivariate index we derived. This result is consistent with conclusions from a previous study of SOF data, which found that among all SOF participants (65 to 89 years of age), contrast sensitivity (and visual acuity) were significantly associated with mortality [38]. Previous analyses have also identified poor contrast sensitivity as a risk-factor for deleterious aging outcomes, such Alzheimer's disease [78] and hip fracture [79]. It is unlikely that the prognostic value of contrast sensitivity in our analysis is due to an association between contrast sensitivity and accidental death (e.g., car accidents), since in our analysis, cases of accidental death were treated as right-censored data. Our findings therefore complement those that have accumulated from studies of other populations, which have documented associations between all-cause mortality and indicators of visual status, such as lens changes [80], poor visual acuity [81], self-reported visual impairment [82], cataract or prior cataract removal [83], age-related macular degeneration [84], retinopathy [85], nuclear sclerotic cataract severity [86] and high intraocular pressure [87]. Many of these associations have been present in both diabetic and non-diabetic populations [85], and it has been speculated that measures of visual function could serve as indicators of biological aging [81,82,84,88]. Contrast sensitivity has proven to be an especially sensitive measure of visual function that correlates with real-world performance on vision-oriented tasks (e.g., driving, reading speed, face recognition) [89-91]. It is known that contrast sensitivity declines with age, although the functional basis of this decline is unclear and may involve multiple factors [92]. To some degree, age-related contrast sensitivity decline could reflect choroidal neovascularization that can accompany development of age-related macular degeneration (AMD) in some older individuals, given that contrast sensitivity scores are lower in AMD patients [93], and that therapies inhibiting choroidal neovascularization in AMD patients improves contrast sensitivity [94]. On the other hand, contrast sensitivity is also impaired by several other ocular diseases (e.g. cataract, glaucoma, diabetic retinopathy) [95-97], suggesting that multiple mechanisms contribute to diminished contrast sensitivity with age. It is interesting to note that contrast sensitivity may represent a general indicator of a subject's sensory perception, and that useful prognostic data might have been obtained from other sensory systems not evaluated during baseline exams (e.g., hearing test). For instance, among men and white women, one study found that associations between vision and hearing impairment with survival were additive, with concurrent vision and hearing loss more strongly associated with mortality than impairment of either sensory system individually [82].

Our results provide a validated index of traits, which predicts survival regardless of the assigned cause of death, and is significantly associated with several other long-term outcomes in addition to survival *per se*. The principle that underlies our index is that favorable combinations of measurements can be identified using a data mining approach, which aims to identify a set of non-redundant variables that predict long-term survival within a cohort for which age-related disease burden is low. The model we have generated by this approach includes many variables known to predict survival and several might have been surmised in advance (e.g., smoking and diabetes status). However, the exact combination of 13-variables (out of 4.05 × 10^23 ^possible 13 variable combinations) is less likely to have been surmised in advance of our study, and likewise, the ranking of variables according to importance as listed in Table 2 was not obvious at the outset. For the purpose of characterizing frailty, previous work has developed a 5-variable "frailty index" [16]. By analogy, our work suggests that a multi-variable index of "healthy aging" might be based upon variables drawn from at least six domains of the aging adult, including (i) a measure of physical function, (ii) smoking status/history, (iii) diabetes status/history, (iv) self-rated health, (v) visual performance and (vi) an indicator of cardiovascular health. The 13-variable index we identified includes specific measures that fall into each of these categories, but we note that substitute variables can be used in some cases with little overall effect on predictive performance. For example, as a measure of physical function, our index suggests that the number of step-ups completed by a subject in 10 seconds was the most informative variable, but concordance estimates of the index decreased only slightly when walking speed or grip strength was substituted for this variable (Table 2). The multivariate index we developed, therefore, should not be interpreted in overly rigid terms, but should be viewed as indicative of certain classes of measurements that are likely to be informative when used in combination, with the precise choice of measurements dictated primarily by practicality of clinical evaluation.

There are both advantages and disadvantages of the methodology we have used to derive an index that predicts long-term survival and which appears to characterize "healthy aging". The main advantage of our approach is that it is data-driven and variables were chosen in an objective fashion using a cross-validation criterion that ensures generalization ability (at least with respect to the SOF cohort we studied). The index we have developed is thus validated in terms of its ability to predict long-term survival patterns, and as we have shown, it also predicts outcomes among survivors that are unrelated to mortality (see Figure 10). A disadvantage of our approach is that, while an index built using data-mining methods may perform well in terms of predictive ability, this does not guarantee that the index will be easy to implement in practice, and it also does not guarantee that index components will fit into a conceptual scheme useful for understanding what "healthy aging" means. To develop a rule-based scheme for characterization of "frailty", for instance, Fried et al. [16] first developed a conceptual framework, devised a rule-based system within this framework, and then validated the predictive validity of the rule-based system using survival data from the Cardiovascular Health Study. The concept-driven approach followed by Fried et al. [16] thus ensures that the index generated can be connected to a broader framework and that the index is sensible from a medical standpoint. None of these assurances can be claimed of the data mining methodology we have implemented in this study. However, it can be argued that an index that fits an elegant theory but is sub-optimal in terms of predictive capacity is, with good reason, less likely to be assigned preference in practical contexts. Moreover, an appealing aspect of data-driven indices is that they provide a suitable and well-supported foundation for building new conceptual schemes. Indeed, the index developed by our analysis is sensible in many respects, given that physical performance measures are frequently advanced as cumulative indicators of general health [33,39,98-102], that smoking has been viewed as accelerating many features of aging [103-105], that diabetes is known to re-enforce age-related declines in telomere length and peripheral blood flow [106-108], that visual indices have been viewed as suitable for measurement of biological age [81,82,84,88], and that the chronological age of subjects was in fact selected as the fourth most-important component of our index (a reassuring "positive control" for our methodology).

We anticipate that, in several ways, further studies will improve and refine the index we have developed here. Our analysis evaluated a wide range of variables that relate to multiple aspects of an aging adult, including past medical history and measures that reflect health status at presentation. It is quite possible, however, that other variables, not represented in our analysis, might have been better predictors of survivorship than those available in the SOF dataset. For example, our investigation did not include biochemical measurements, such as indicators of systemic inflammation [73], or markers based upon gene expression in blood cells [109], or genotype information derived from single nucleotide polymorphisms [110]. It would be valuable to determine how such measures compete with those identified in our analysis, and whether any of these measures would contribute useful information to the index that we have developed. A second avenue for improvement is further validation of our index as a measure of healthy aging that reflects multiple forms of age-related illness. The index we developed was able to discriminate survival times among subsets of subjects for which the assigned cause of death was cancer, cardiovascular disease or non-cancer/non-cardiovascular disease. It is likely, however, that there exists some degree of overlap among these categories, which is not reflected in available SOF data, as well as some variance in the degree of certainty associated with the assigned cause of death. We therefore anticipate that analytical methods used in our analysis can be profitably applied in other contexts, possibly with narrowed and more fine-scaled cause of death categorizations, which would serve to further evaluate the index as a measure of an individual's aging trajectory that is sensitive to multiple disease processes. Lastly, the index we have generated has been validated primarily among community-dwelling Caucasian women between the ages of 65 and 69. We have evaluated the performance of this index with respect to older subjects from the SOF cohort (i.e., ages 70-89; see Additional File 4), and have found that its prognostic value declines with age, suggesting that certain indicators of long-term survival in younger populations (e.g., smoking and diabetes status) may not provide ideal tools for predicting comparatively short-term outcomes in older cohorts. We therefore expect that the index we developed will be most useful when applied to subjects that fall within a specific age bracket (i.e., 65-69 years of age, approximately). Moreover, with respect to subjects of this age, further validation of the index is necessary to determine whether findings from this study generalize to other independent cohorts, particularly cohorts that include subjects of both genders and a broad range of ethnic backgrounds and environmental settings. The most useful index to clinicians, as well as to research investigators, will most likely consist of variables that consistently emerge as the strongest predictors in multiple epidemiological datasets that include a comprehensive range of measures.

The translation of findings from basic aging research to practical anti-aging interventions would benefit greatly from the identification of variables that best predict future health outcomes in rodent models and people. Direct tests of putative anti-aging interventions for effects on survival in human populations would be extremely, perhaps prohibitively, expensive. In contrast, surrogate endpoints that are robustly associated with both long-term survival and exceptionally good health might be used in research trials to infer whether an intervention has favorably altered the odds of long-term survival. Some of the variables highlighted in our analysis exhibit increasing or decreasing trends across age groups, and likewise, risk scores generated from the index we developed change monotonically with age (see Additional File 2). We therefore propose that certain age-sensitive variables identified in our study be considered for inclusion within a panel of "validation measures", that once developed, could serve as a standard set of traits to be evaluated in human studies of anti-aging interventions. So far, clinical studies of anti-aging interventions, such as caloric restriction without malnutrition [23], have not evaluated measures of physical performance, contrast sensitivity, or most other variables included in the index we have developed. Ultimately, however, we believe that the determination of whether an intervention alters the rate of aging in humans (or other species) should not be based upon idiosyncratic sets of measures chosen by particular research groups, but rather, upon validated sets of measures that have emerged from independent global analyses of large datasets, which are in this fashion shown to be dominant predictors of long-term health and survivorship outcomes. An additional benefit from the identification of variables robustly associated with long-term human survival is the potential that, by "reverse translation", such variables will suggest new endpoints for evaluation in basic aging research using model organisms [111,112]. Indeed, there are already several examples in which analogues of human physical performance traits have been successfully modeled in mice, worms and flies [113-115]. We anticipate that further development and characterization of a robust healthy aging phenotype in humans, based upon global analyses of comprehensive datasets, will promote further work along these lines and enhance the synergy between basic and applied aging research.

## Conclusions

The recognition of healthy aging patterns and prediction of long-term survival is an important problem in clinical contexts with implications for the design of intervention studies that aim to evaluate anti-aging treatments. This investigation has focused on the challenging task of predicting long-term (20-year) survival within a healthy cohort of 4,097 women (ages 65 - 69) enrolled in the Study of Osteoporotic Fractures (SOF). Surprisingly, among 377 predictor variables evaluated, contrast sensitivity scores were among the strongest predictors of survival (ranked 5th of 377 variables, mean AUC = 0.570). We have implemented a data mining approach to develop a multivariate index that predicts mortality patterns among all 4,097 subjects (mean AUC = 0.673), as well as within sub-cohorts for which the assigned cause of death was cancer, cardiovascular disease, or non-cancer/non-cardiovascular disease. Among subjects that survived an average follow-up time of 20 years, this index was associated with multiple outcomes, including tests of cognitive function, geriatric depression, number of daily living impairments and grip strength. The index we present requires further validation with respect to other cohorts. However, our results suggest that components of our index characterize the clinical presentation of "healthy aging" as it frequently occurs in older women between 65 and 69 years of age. We suggest that our data-guided approach to index development can be profitably applied to other comprehensive datasets to further develop standardized criteria for recognizing healthy aging in older adults.

## Competing interests

The authors declare that they have no competing interests.

## Authors' contributions

WRS performed the statistical analyses and co-wrote the manuscript. RAM planned the study, co-wrote the manuscript and critically reviewed the manuscript. KEE, PMC, JAC and SRC critically reviewed the manuscript and assisted in interpretation of the findings. All authors read and approved the final manuscript.

## Pre-publication history

The pre-publication history for this paper can be accessed here:

http://www.biomedcentral.com/1471-2318/10/55/prepub

## Supplementary Material

Additional file 1 **Overview of SOF Cohort Evaluated in this Study**. This file provides a flow chart diagram describing the filtering of the 9704 subjects within the SOF database (ages 65 - 89), along with an overview of assigned causes of death among the 4097 subjects (ages 65 - 69) included in our analyses. Click here for file

Additional file 2 **List of 377 Variables Included in Analysis**. This file provides a list of the 377 variables evaluated in the analysis. For each variable, hazard ratio estimates are provided along with 95% confidence intervals and associated p-values. Hazard ratios are estimated within univariate Cox regression models and also within models that have been adjusted for subject age, smoking and diabetes status. A separate list of 88 variables excluded from the analysis due to > 5% missing values is also provided. Click here for file

Additional file 3 **Cross-Sectional Association of Index Components with Subject Age**. This file provides analyses of relationships between selected index components and subject age, as well as the relationship between index-generated risk scores and subject age. Click here for file

Additional file 4 **Evaluation of index performance with respect to older subjects from the SOF cohort (70 - 89 years of age)**. The 13-variable index we present (Table 2) was developed based upon survival patterns among the youngest subjects of the SOF cohort (i.e., ages 65 - 69). It was of interest, however, to evaluate the prognostic value of the index with respect to older SOF subjects (i.e., ages 70 - 89), since this would provide further validation of our index based upon an independent cohort. Additionally, this analysis was expected to provide some insight into the prognostic value of the index among older SOF subjects (ages 70-89) relative to the younger SOF subjects (ages 65-69) that were the main focus of our investigation. This file therefore provides a cross-validation evaluation of the 13-variable model with respect to SOF subjects aged 70-74 (*n *= 3033), 75-79 (*n *= 1538), 80-84 (*n *= 765) and 85-89 (*n *= 228). Performance with respect to each age group was evaluated using 10,000 cross-validation trials, with correspondence between observed and predicted survival patterns assessed using the concordance metric (*C*) described in the Methods section and applied elsewhere in this paper. Click here for file

Additional file 5 **Random Variable Selection as a Modeling Strategy**. Previous investigations have suggested that exact specification of variables is not always an important factor determining the performance of a prognostic model. This file therefore contains analyses that compare prognostic performance of the 13-variable model generated in our study (see Table 2) with the performance of 13-variable models that include variables chosen at random from a pool of variables (either with or without pre-filtering of the variable pool). We note that sensitivity of model performance to variable specification is expected to increase for models that are based upon a smaller number of predictor variables. Click here for file

Additional file 6 **Comparison of model performance to an index that quantifies the accumulation of deficits or health problems**. Previous studies have shown that high-quality predictive models can be generated based upon an index that appropriately reflects the accumulation of health problems or "deficits". Such deficits should exhibit greater frequency with age, be indicative of health status, but should not be universally present among older subjects. Examples of such deficits include the presence of depression, weight loss, difficulty with daily living tasks and a history of heart disease. This file generates an "SOF Deficit Index" and compares the performance of this index to that of the 13-variable healthy aging index presented in Table 2. The comparison is made with respect to the young SOF subjects that are the main focus of this paper and also with respect to older SOF cohorts aged 70-74 (*n *= 3033), 75-79 (*n *= 1538), 80-84 (*n *= 765) and 85-89 (*n *= 228). Click here for file
