# Supplementary material for: Indicators of "Healthy Aging" in older women (65-69 years of age). A data-mining approach based on prediction of long-term survival
Source: BMC Geriatr. 2010 Aug 17;10:55. doi: 10.1186/1471-2318-10-55 (PMC2936300; doi:10.1186/1471-2318-10-55)

Additional File 1

Indicators of "Healthy Aging" in Older Women (65-69 years of age). A Data-mining Approach based on Prediction of Long-term Survival.

William R. Swindell, Kristine E. Ensrud, Peggy M. Cawthon, Jane A. Cauley, Steve R. Cummings, Richard A. Miller

---

Overview of SOF Cohort Evaluated in this Study

This file provides a flow chart diagram describing the filtering of SOF subjects and characteristics of the SOF subjects included within the sub-cohort considered in our analyses. A total of n = 9704 subjects were filtered to exclude those above the age of 69, leaving a sub-cohort of n = 4097 subjects upon which further analyses were based. This filtering focused model building efforts on identifying long-term predictors of survival among healthier subjects, rather than short-term predictors of survival within a set of elderly subjects with greater prevalence of frailty. Of the 4097 subjects, n = 1523 mortalities occurred during follow-up. Of these, 467 were attributed to atherosclerosis, 426 were attributed to cancer, and 586 were attributed to neither atherosclerosis nor cancer. Among the 586 mortalities in this last category, 44 were attributable to accidental causes and treated as censored survival times in our analyses. We note that there is likely to exist some degree of overlap among apparently distinct causes of death, and that some degree of error is expected in the cause of death assigned to older individuals.

Contact: William R. Swindell, wswindel@umich.edu

Overview of SOF Cohort Evaluated in this Study

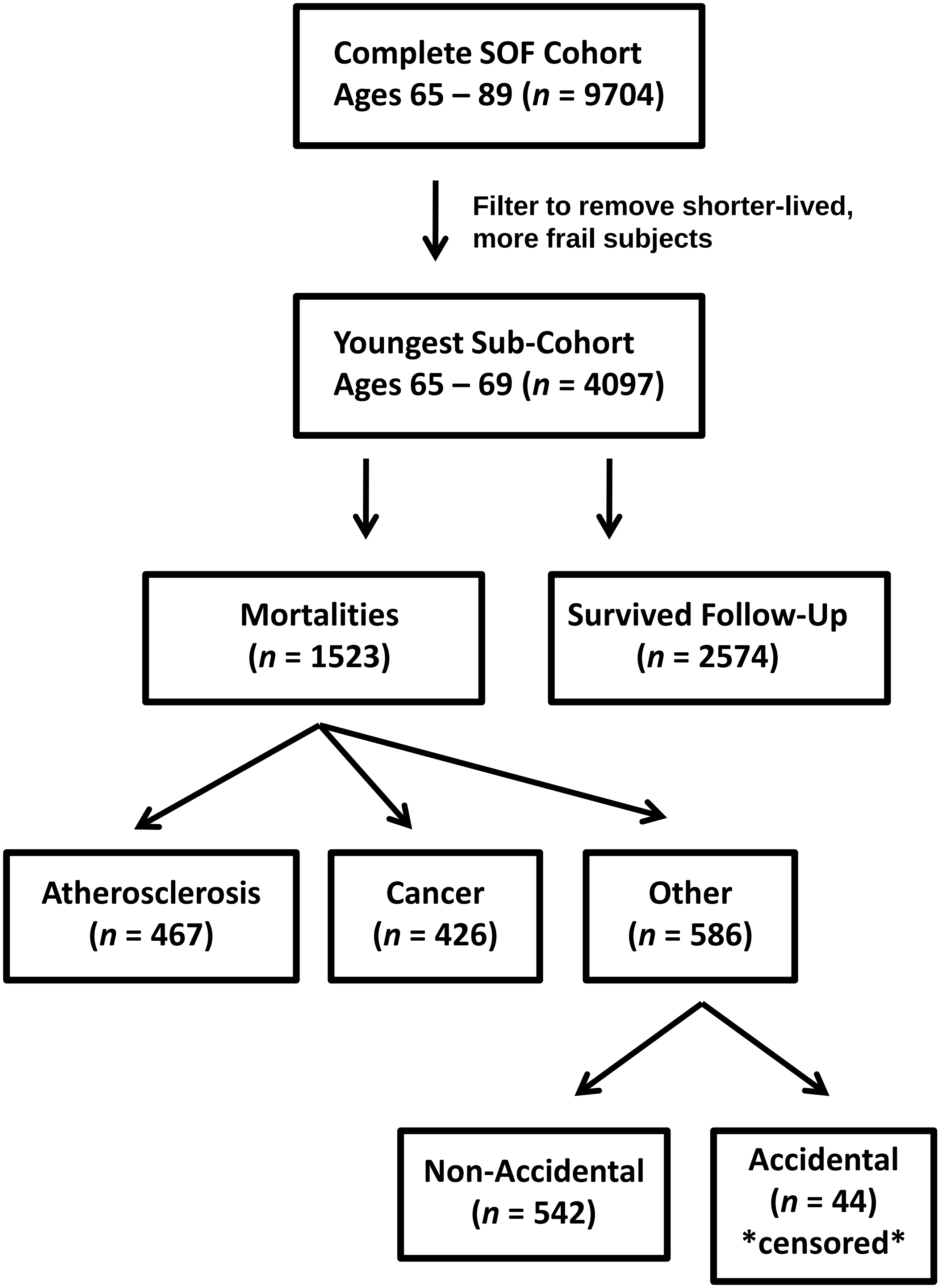

Supplement: Additional file 1 — Overview of SOF Cohort Evaluated in this Study. This file provides a flow chart diagram describing the filtering of the 9704 subjects within the SOF database (ages 65 - 89), along with an overview of assigned causes of death among the 4097 subjects (ages 65 - 69) included in our analyses. [file 1471-2318-10-55-S1.PDF]
