# Supplementary material for: Indicators of "Healthy Aging" in older women (65-69 years of age). A data-mining approach based on prediction of long-term survival
Source: BMC Geriatr. 2010 Aug 17;10:55. doi: 10.1186/1471-2318-10-55 (PMC2936300; doi:10.1186/1471-2318-10-55)
Supplement: Additional file 3 — Cross-Sectional Association of Index Components with Subject Age. This file provides analyses of relationships between selected index components and subject age, as well as the relationship between index-generated risk scores and subject age. [file 1471-2318-10-55-S3.PDF]

Additional File 3

Indicators of "Healthy Aging" in Older Women (65-69 years of age). A Data-mining Approach based on Prediction of Long-term Survival.

William R. Swindell, Kristine E. Ensrud, Peggy M. Cawthon, Jane A. Cauley, Steve R. Cummings, Richard A. Miller

---

Cross-Sectional Association of Index Components with Subject Age

This file provides analyses of relationships between selected index components and subject age (Figures A - D), as well as the relationship between index-generated risk scores and subject age (Figure E). The 13-variable index generated by our analysis included four continuous variables that were age-sensitive (i.e., number of step ups completed in 10 seconds, contrast sensitivity score, pulse lying down and self-reported height loss since the age of 25). For each of these variables, figures (A) - (D) show the association of these measures with the baseline age of subjects, based upon the complete SOF cohort (approximately 9600 subjects). In figures (A) - (D), each point represents the average value associated with each age group, and error bars correspond to ± one standard error of the mean. The dashed red line represents a linear model fit obtained by the MM-estimation technique described by Yohai 1987 (High breakdown-point and high efficiency estimates for regression, *The Annals of Statistics* 15, 642–65), which is a robust regression technique designed to attenuate the influence of outlying observations.

In figure (E), risk scores were calculated for 9333 subjects (those with measures available for each of the 13 variables included in our index), and these risk scores are plotted with respect to subject age. Black symbols correspond to individual subjects, while blue symbols represent the average risk score associated with each age group, with error bars indicating ± one standard error of the mean. Risk scores have been normalized such that the average risk score among all 9333 subjects is equal to zero, which is represented by the dashed black horizontal line in figure (E). The dashed red line corresponds to a linear model fit obtained by MM-estimation of model coefficients (see above). It should be noted that some type of association between risk scores and age is expected, because age is one of the 13 variables included in the Cox model used to generate risk scores (see Table 2). Figure (E) illustrates that risk scores have an association with age that is linear, and that average risk scores for each age group are strongly correlated with age. Among individuals, some old subjects are assigned low risk scores, while some young subjects are assigned high risk scores, due to variation among individuals with respect to the other 12 variables included in the index that we developed.

Contact: William R. Swindell, wswindel@umich.edu

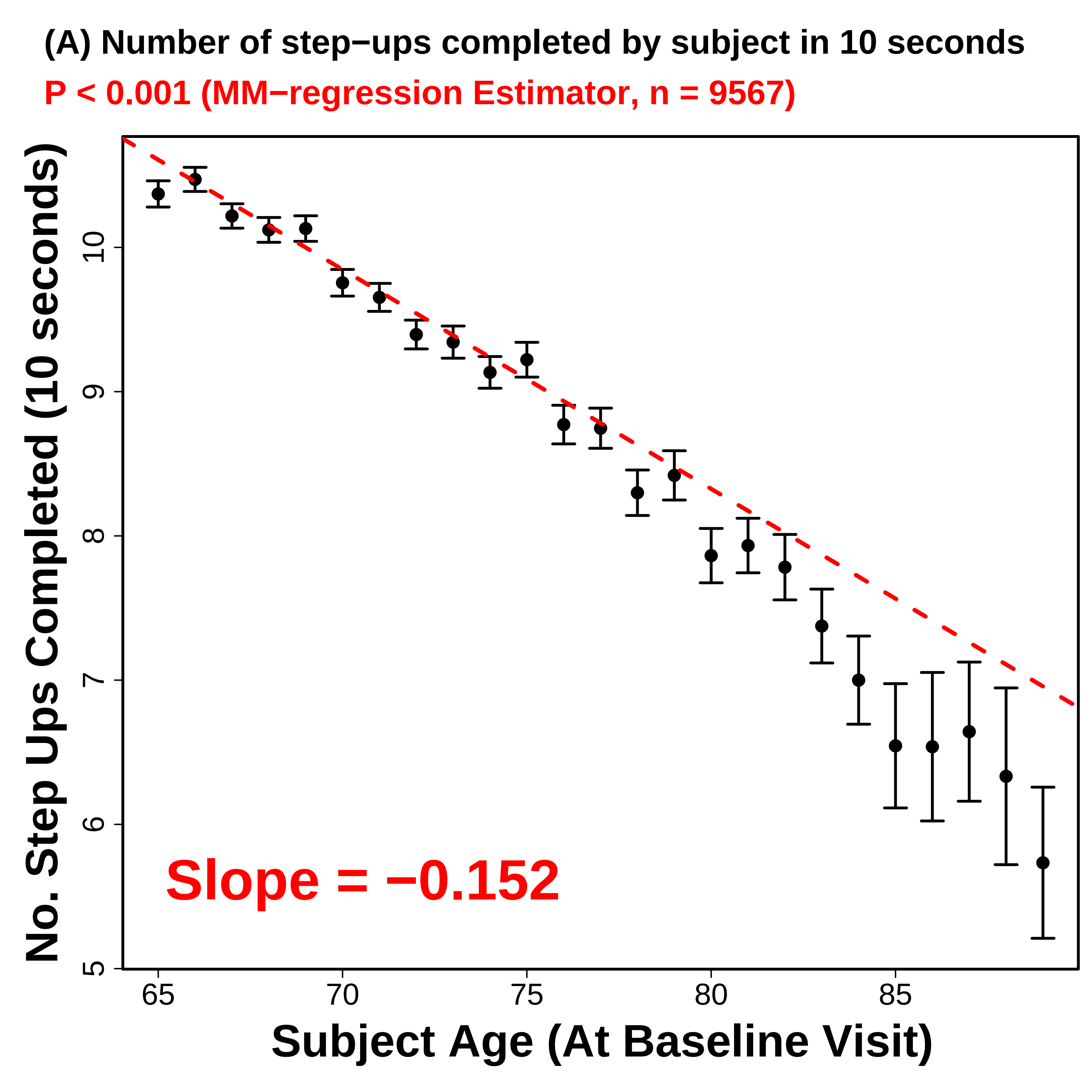
(A) Number of step−ups completed by subject in 10 seconds
P < 0.001 (MM−regression Estimator, n = 9567)
No. Step Ups Completed (10 seconds)
5
6
7
8
9
10
65
70
75
80
85
Subject Age (At Baseline Visit)
Slope = −0.152

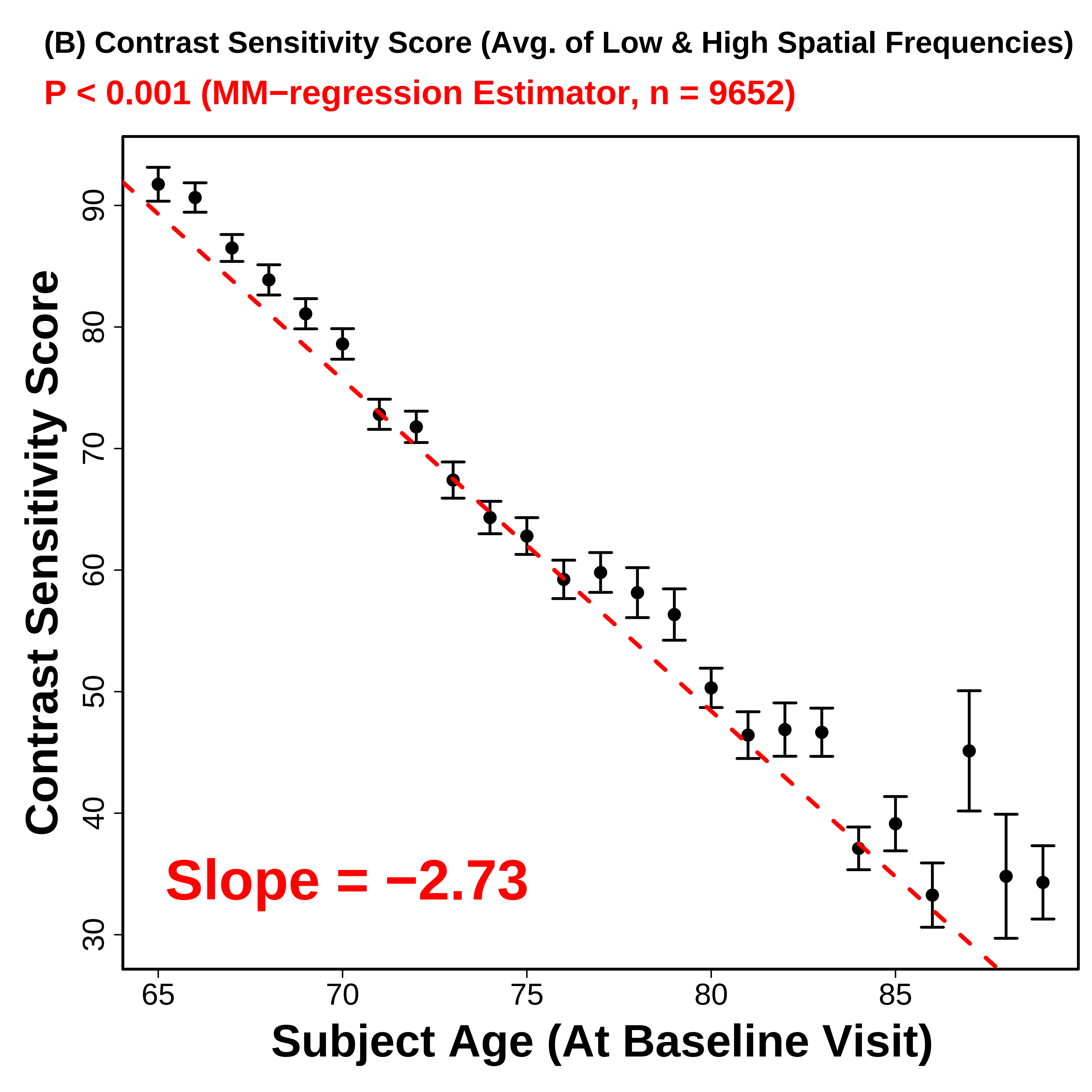
(B) Contrast Sensitivity Score (Avg. of Low & High Spatial Frequencies)
P < 0.001 (MM−regression Estimator, n = 9652)
Contrast Sensitivity Score
Subject Age (At Baseline Visit)
Slope = −2.73
30
40
50
60
70
80
90
65
70
75
80
85

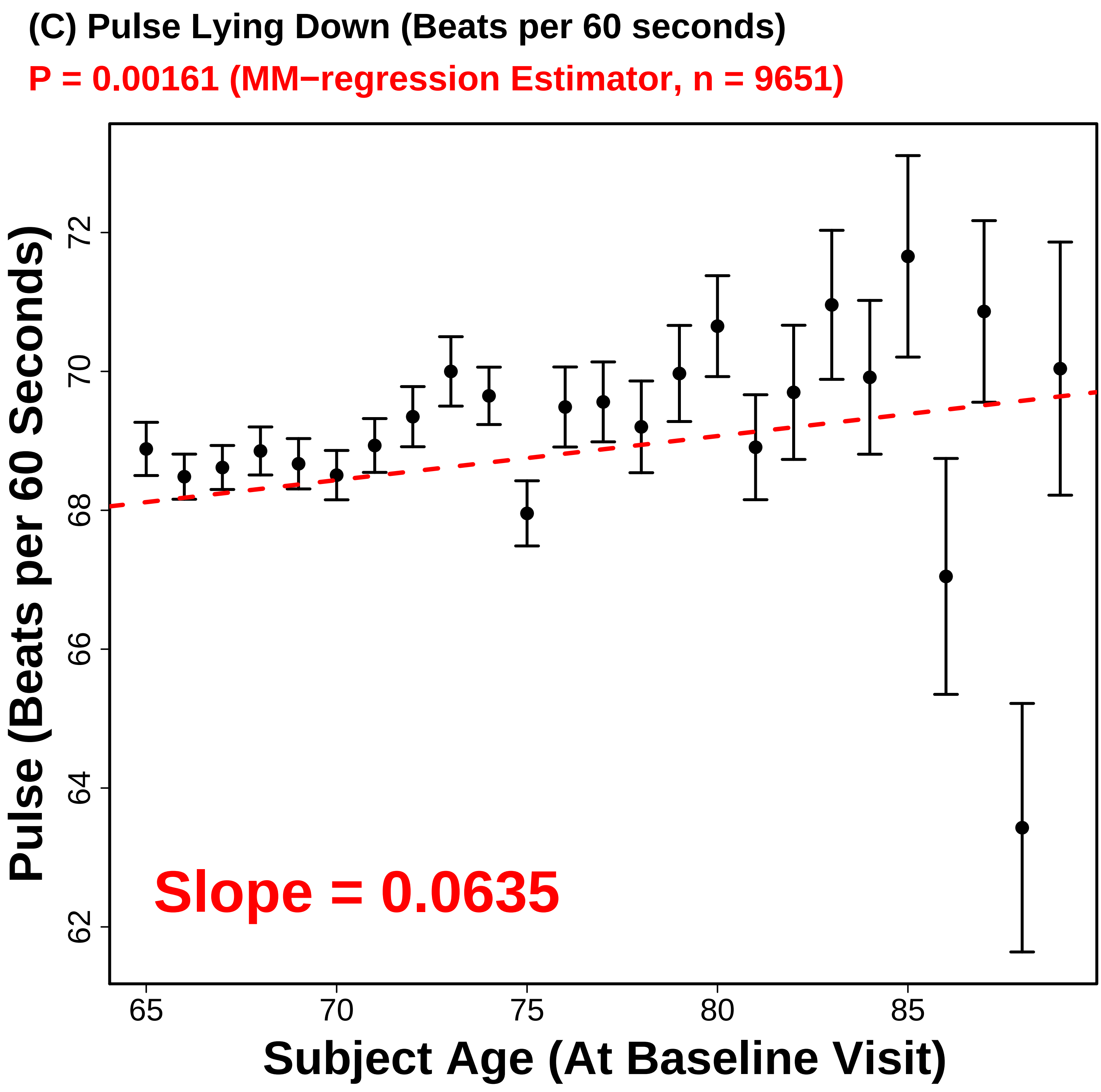
(C) Pulse Lying Down (Beats per 60 seconds)
P = 0.00161 (MM−regression Estimator, n = 9651)
Pulse (Beats per 60 Seconds)
62
64
66
68
70
72
Slope = 0.0635
65
70
75
80
85
Subject Age (At Baseline Visit)

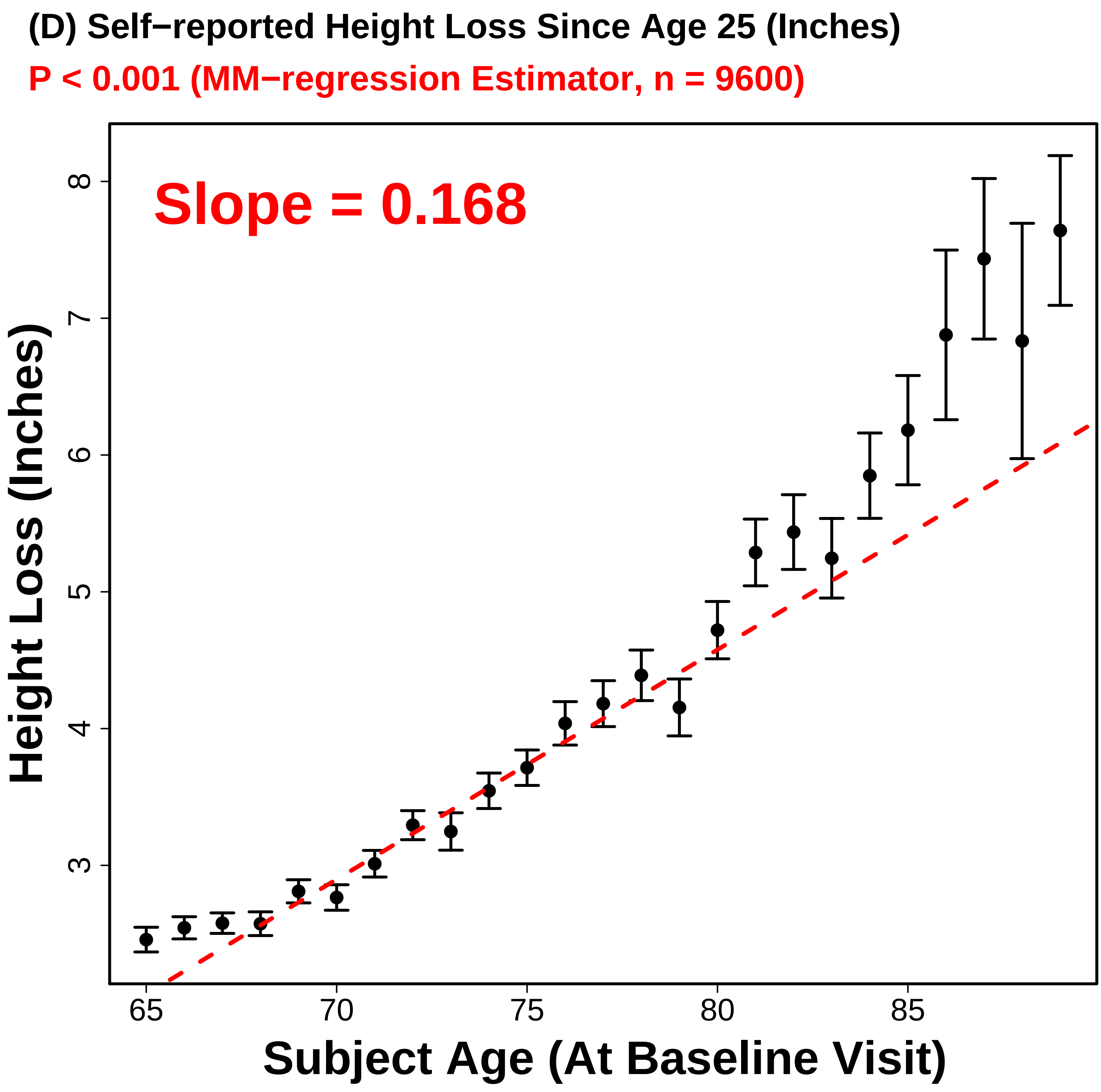
(D) Self−reported Height Loss Since Age 25 (Inches)
P < 0.001 (MM−regression Estimator, n = 9600)
Slope = 0.168
Height Loss (Inches)
3
4
5
6
7
8
65
70
75
80
85
Subject Age (At Baseline Visit)

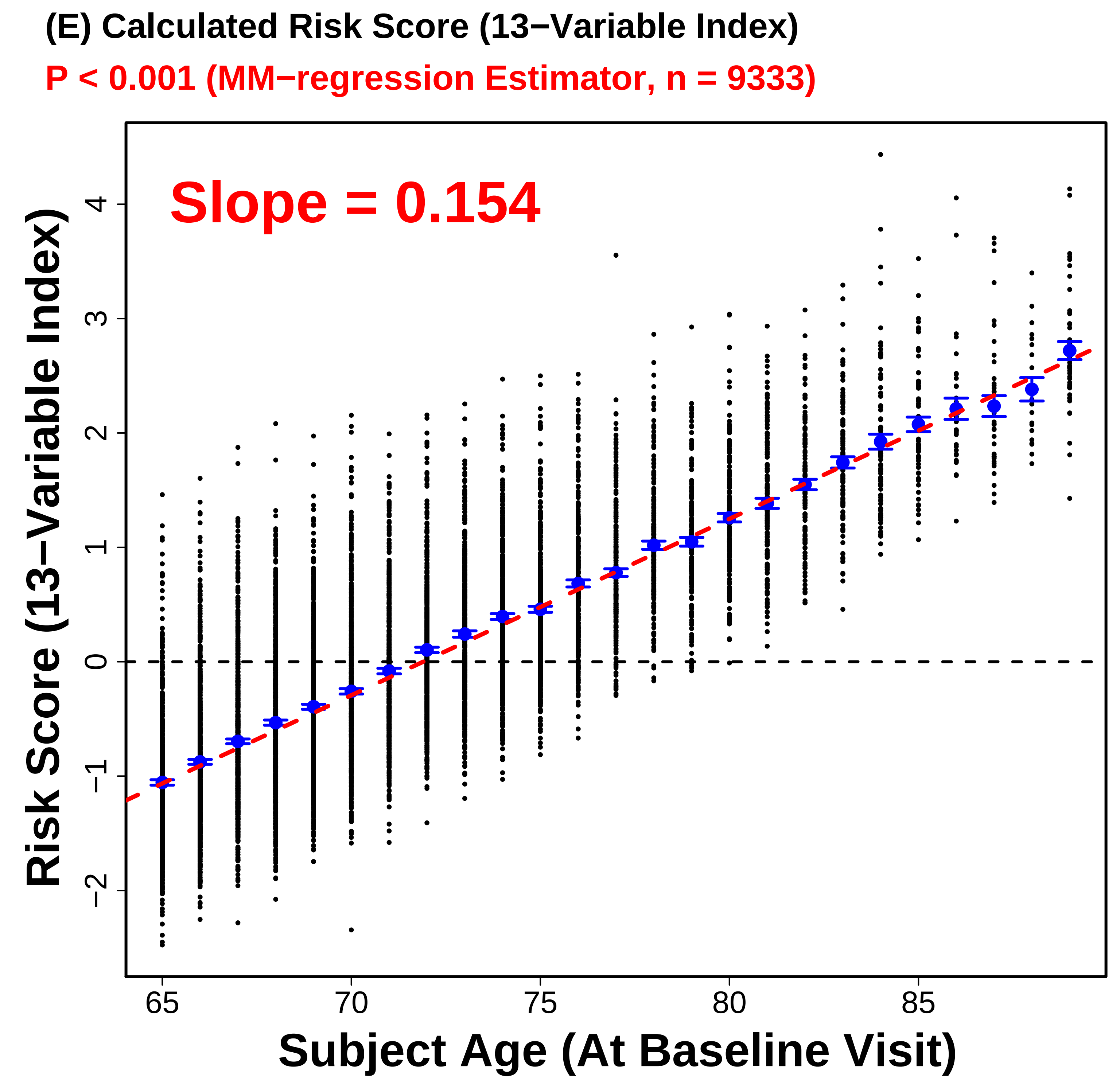

(E) Calculated Risk Score (13−Variable Index)
P < 0.001 (MM−regression Estimator, n = 9333)
Slope = 0.154
Risk Score (13−Variable Index)
−2
−1
0
1
2
3
4
65
70
75
80
85
Subject Age (At Baseline Visit)
