# Supplementary material for: Indicators of "Healthy Aging" in older women (65-69 years of age). A data-mining approach based on prediction of long-term survival
Source: BMC Geriatr. 2010 Aug 17;10:55. doi: 10.1186/1471-2318-10-55 (PMC2936300; doi:10.1186/1471-2318-10-55)
Supplement: Additional file 4 — Evaluation of index performance with respect to older subjects from the SOF cohort (70 - 89 years of age). The 13-variable index we present (Table 2) was developed based upon survival patterns among the youngest subjects of the SOF cohort (i.e., ages 65 - 69). It was of interest, however, to evaluate the prognostic value of the index with respect to older SOF subjects (i.e., ages 70 - 89), since this would provide further validation of our index based upon an independent cohort. Additionally, this analysis was expected to provide some insight into the prognostic value of the index among older SOF subjects (ages 70-89) relative to the younger SOF subjects (ages 65-69) that were the main focus of our investigation. This file therefore provides a cross-validation evaluation of the 13-variable model with respect to SOF subjects aged 70-74 (n = 3033), 75-79 (n = 1538), 80-84 (n = 765) and 85-89 (n = 228). Performance with respect to each age group was evaluated using 10,000 cross-validation trials, with correspondence between observed and predicted survival patterns assessed using the concordance metric (C) described in the Methods section and applied elsewhere in this paper. [file 1471-2318-10-55-S4.PDF]

Additional File 4

Indicators of "Healthy Aging" in Older Women (65-69 years of age). A Data-mining Approach based on Prediction of Long-term Survival.

William R. Swindell, Kristine E. Ensrud, Peggy M. Cawthon, Jane A. Cauley, Steve R. Cummings, Richard A. Miller

---

Evaluation of Model Performance among Older SOF Subjects (70-89 years of age)

The 13-variable healthy aging index we have developed (Table 2) was constructed based upon survival patterns among the youngest SOF subjects (i.e., ages 65-69). Index performance was validated with respect to survival patterns associated with this particular group of SOF subjects. To validate the index with respect to independent groups of subjects, we also assessed performance of the index with respect to older cohorts from the SOF study (i.e., 70-74, 75-79, 80-84 and 85-89 years of age).

We considered both the prognostic performance of multivariate Cox models with all 13 index variables (Figure A) as well as the performance of univariate Cox models containing only one of the 13 identified variables (Figures B - N). In each case, prognostic performance was assessed using 10,000 cross-validation trials. In each trial, 90% of subjects within a particular age cohort were used as training data (used to estimate model coefficients), with the remaining 10% of subjects within the age cohort used as testing data (used to evaluate predictive accuracy of the trained model) (see Methods). Correspondence between observed and predicted survival times in each simulation was evaluated using the Concordance index (C) (see Methods).

In each figure (A) - (N), bar plots indicate the mean concordance estimate obtained with respect to each of the five age cohorts (i.e., the average value of C obtained across 10,000 cross-validation trials). Error bars correspond to standard deviation estimates among the 10,000 concordance estimates generated from simulation trials performed for each age cohort (standard errors are approximately ± 0.001). Values in red correspond to the height of the bar (i.e., average C value for a given age group), along with associated standard deviation estimates (in parentheses).

Performance of the healthy aging index declined with respect to increasingly older SOF subjects (Figure A). This trend indicates that prognostic value of the index is lower when applied to older subjects, which is likely attributable to the fact that the index was developed using survival patterns of the youngest SOF subjects (ages 65-69).

Contact: William R. Swindell, wswindel@umich.edu

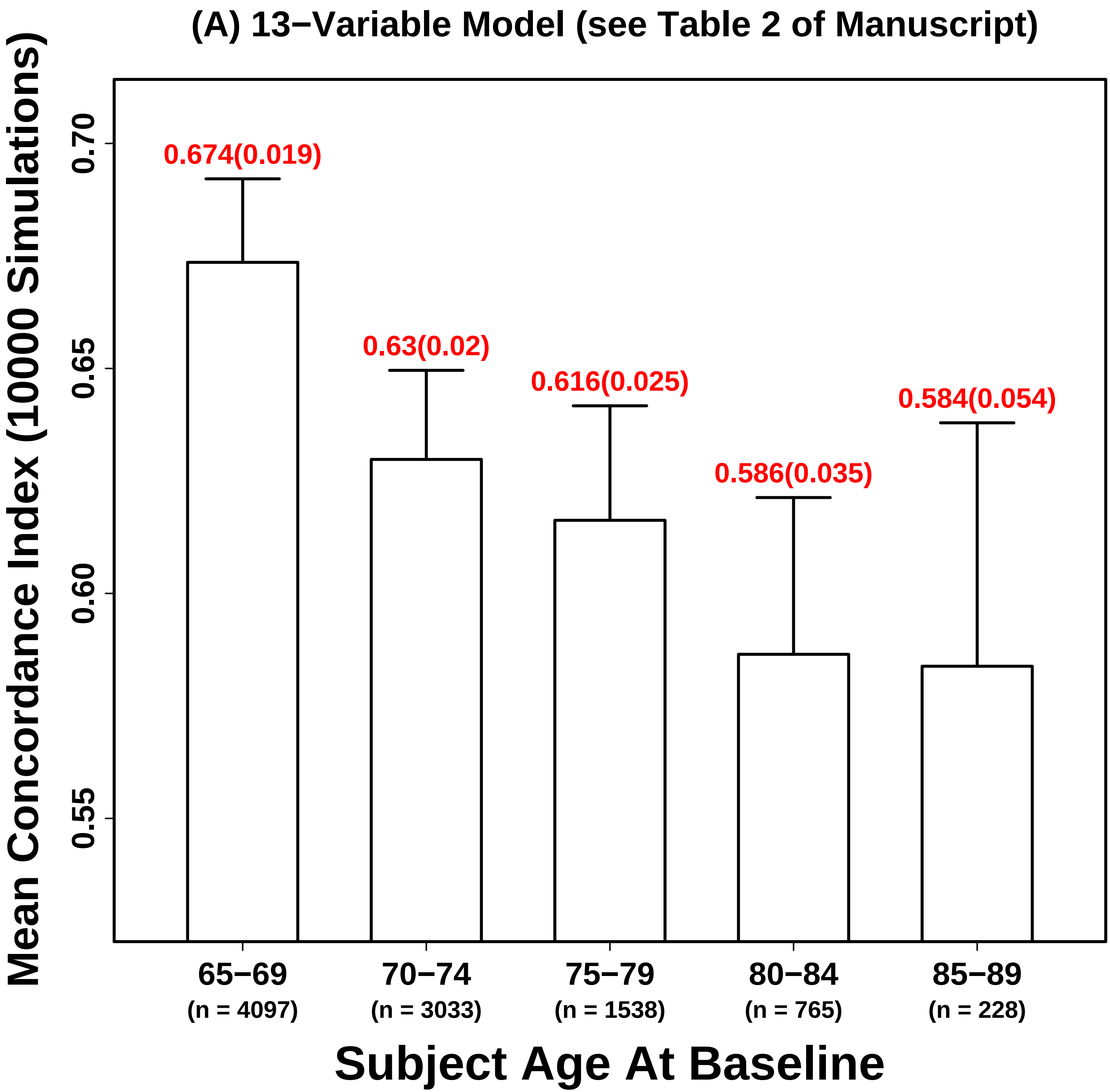

(A) 13−Variable Model (see Table 2 of Manuscript)
Mean Concordance Index (10000 Simulations)
0.70
0.65
0.60
0.55
0.674(0.019)
0.63(0.02)
0.616(0.025)
0.586(0.035)
0.584(0.054)
65−69
(n = 4097)
70−74
(n = 3033)
75−79
(n = 1538)
80−84
(n = 765)
85−89
(n = 228)
Subject Age At Baseline

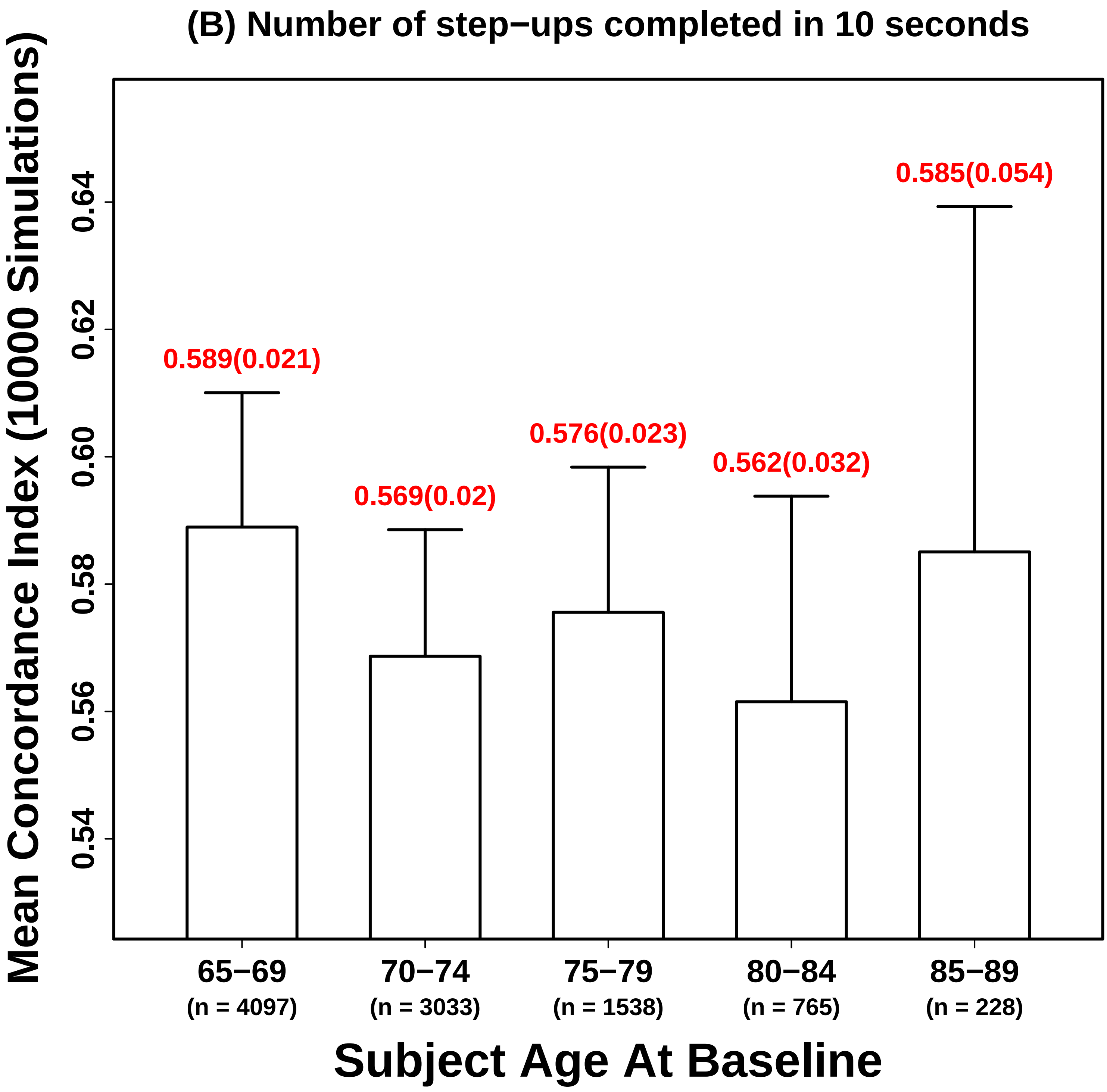

(B) Number of step−ups completed in 10 seconds
Mean Concordance Index (10000 Simulations)
0.54
0.56
0.58
0.60
0.62
0.64
0.589(0.021)
0.569(0.02)
0.576(0.023)
0.562(0.032)
0.585(0.054)
65−69
(n = 4097)
70−74
(n = 3033)
75−79
(n = 1538)
80−84
(n = 765)
85−89
(n = 228)
Subject Age At Baseline

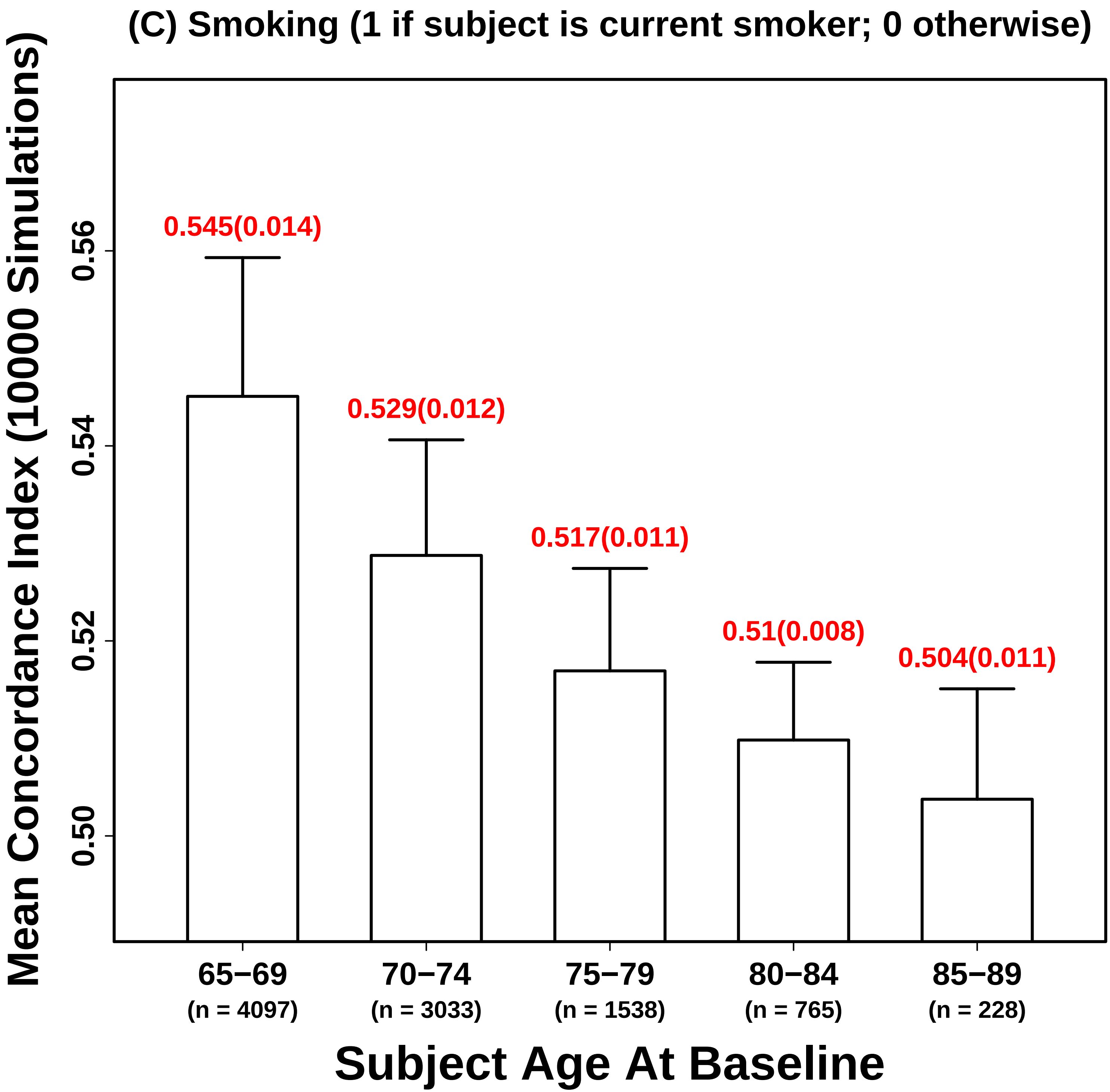

(C) Smoking (1 if subject is current smoker; 0 otherwise)
Mean Concordance Index (10000 Simulations)
0.50
0.52
0.54
0.56
0.545(0.014)
0.529(0.012)
0.517(0.011)
0.51(0.008)
0.504(0.011)
65−69
(n = 4097)
70−74
(n = 3033)
75−79
(n = 1538)
80−84
(n = 765)
85−89
(n = 228)
Subject Age At Baseline

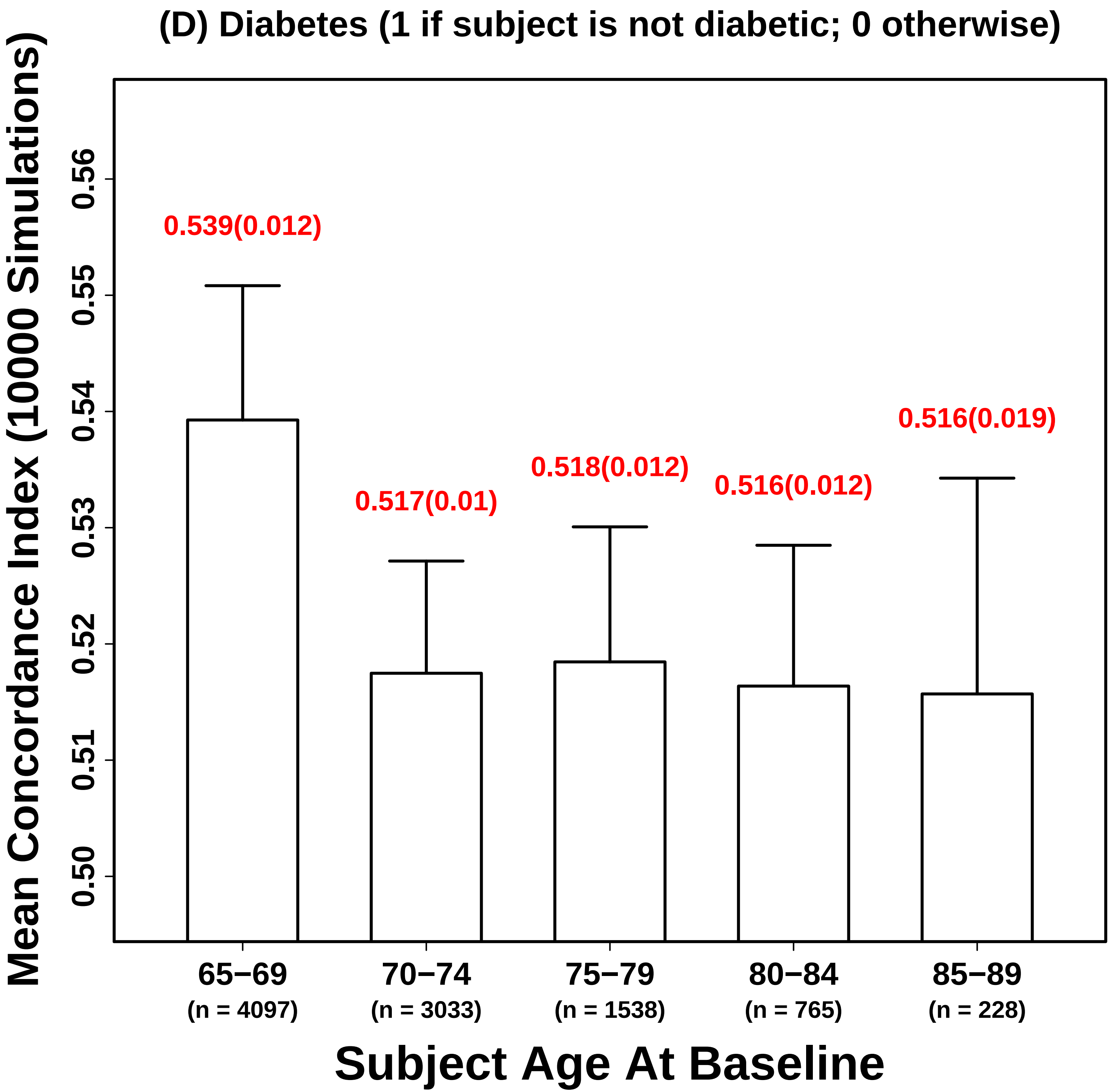
(D) Diabetes (1 if subject is not diabetic; 0 otherwise)
Mean Concordance Index (10000 Simulations)
0.50
0.51
0.52
0.53
0.54
0.55
0.56
0.539(0.012)
0.517(0.01)
0.518(0.012)
0.516(0.012)
0.516(0.019)
65−69
(n = 4097)
70−74
(n = 3033)
75−79
(n = 1538)
80−84
(n = 765)
85−89
(n = 228)
Subject Age At Baseline

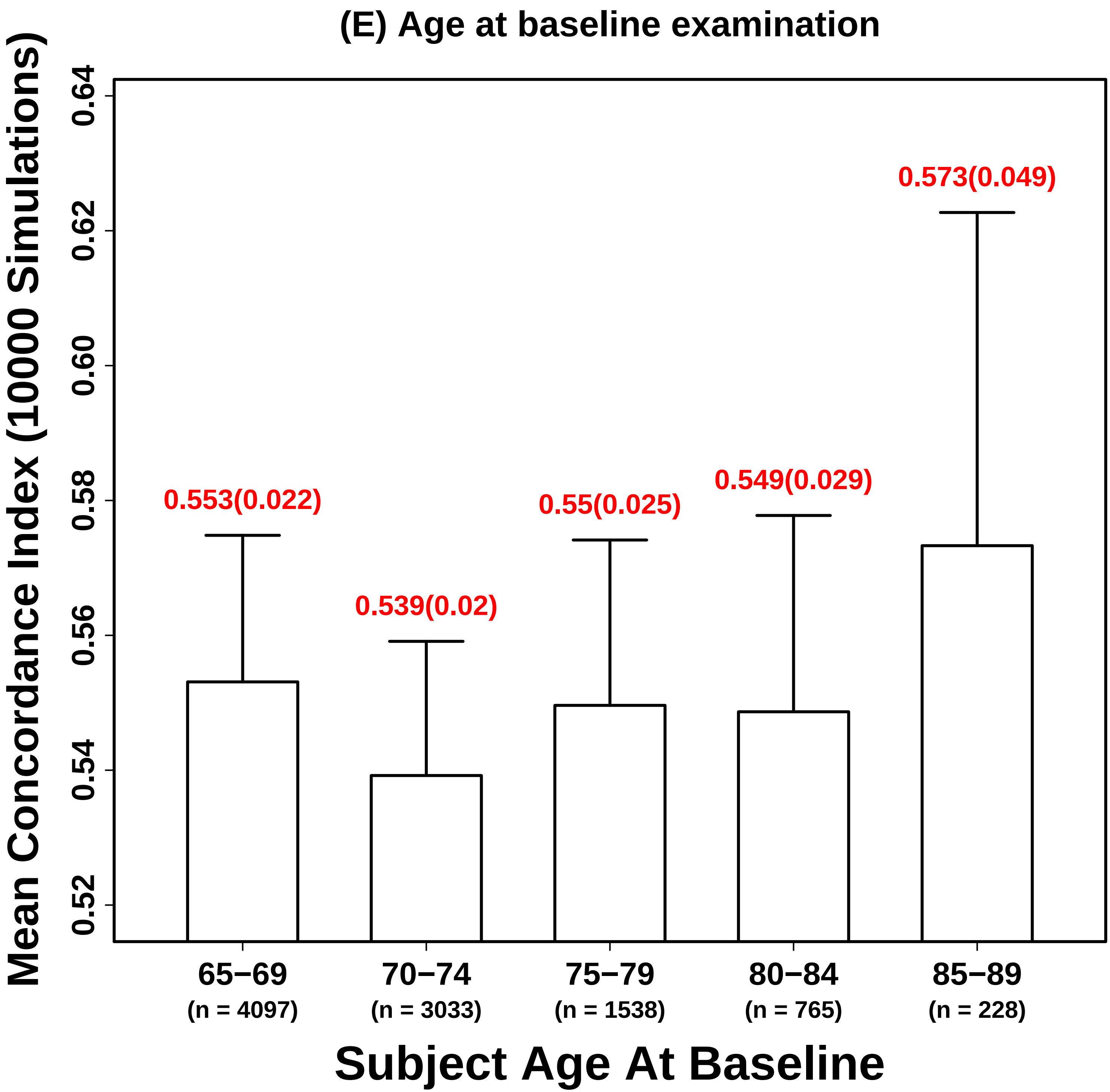
(E) Age at baseline examination
Mean Concordance Index (10000 Simulations)
0.52
0.54
0.56
0.58
0.60
0.62
0.64
0.553(0.022)
0.539(0.02)
0.55(0.025)
0.549(0.029)
0.573(0.049)
65−69
(n = 4097)
70−74
(n = 3033)
75−79
(n = 1538)
80−84
(n = 765)
85−89
(n = 228)
Subject Age At Baseline

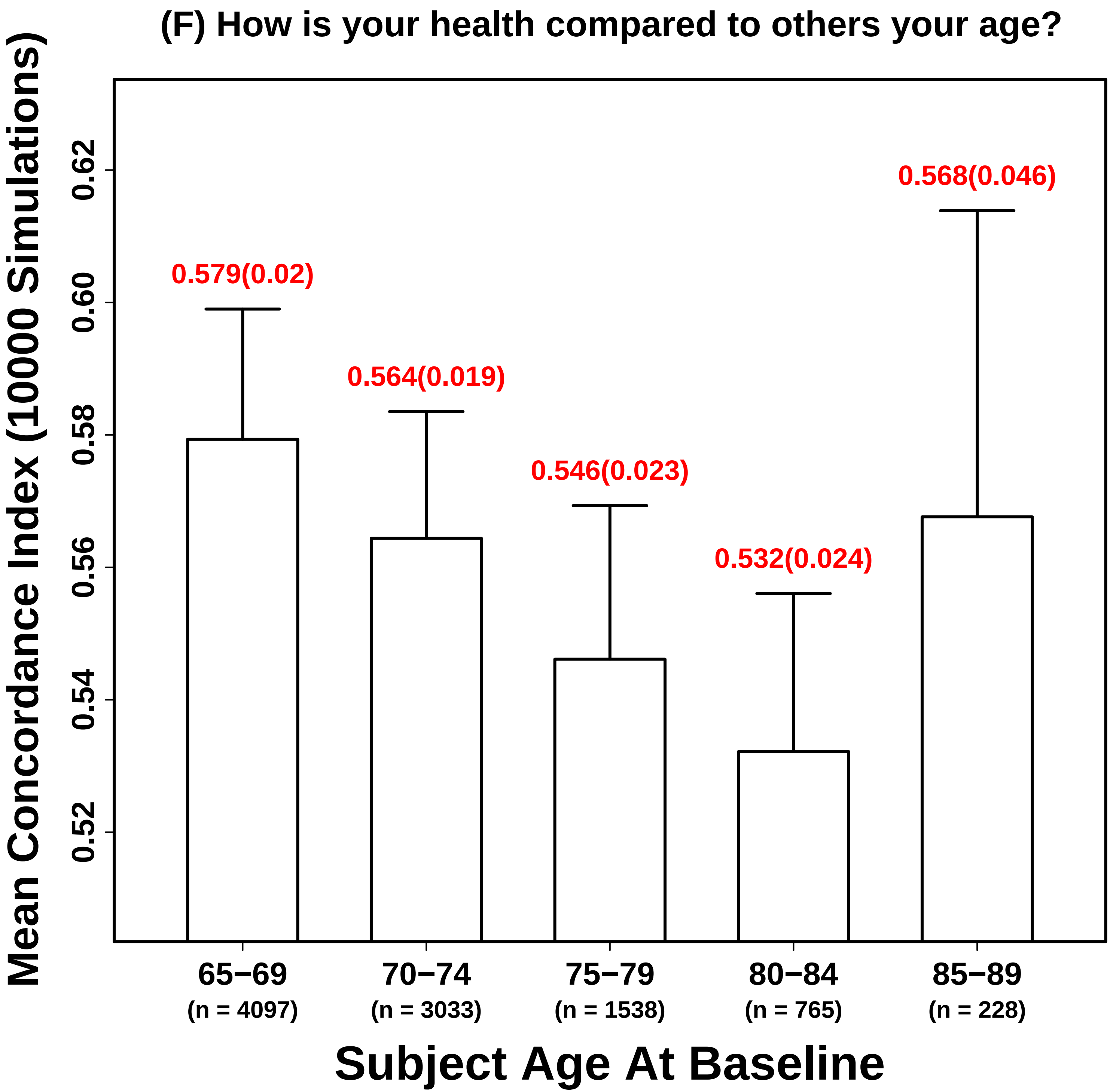
(F) How is your health compared to others your age?
Mean Concordance Index (10000 Simulations)
0.52
0.54
0.56
0.58
0.60
0.62
0.579(0.02)
0.564(0.019)
0.546(0.023)
0.532(0.024)
0.568(0.046)
65−69
(n = 4097)
70−74
(n = 3033)
75−79
(n = 1538)
80−84
(n = 765)
85−89
(n = 228)
Subject Age At Baseline

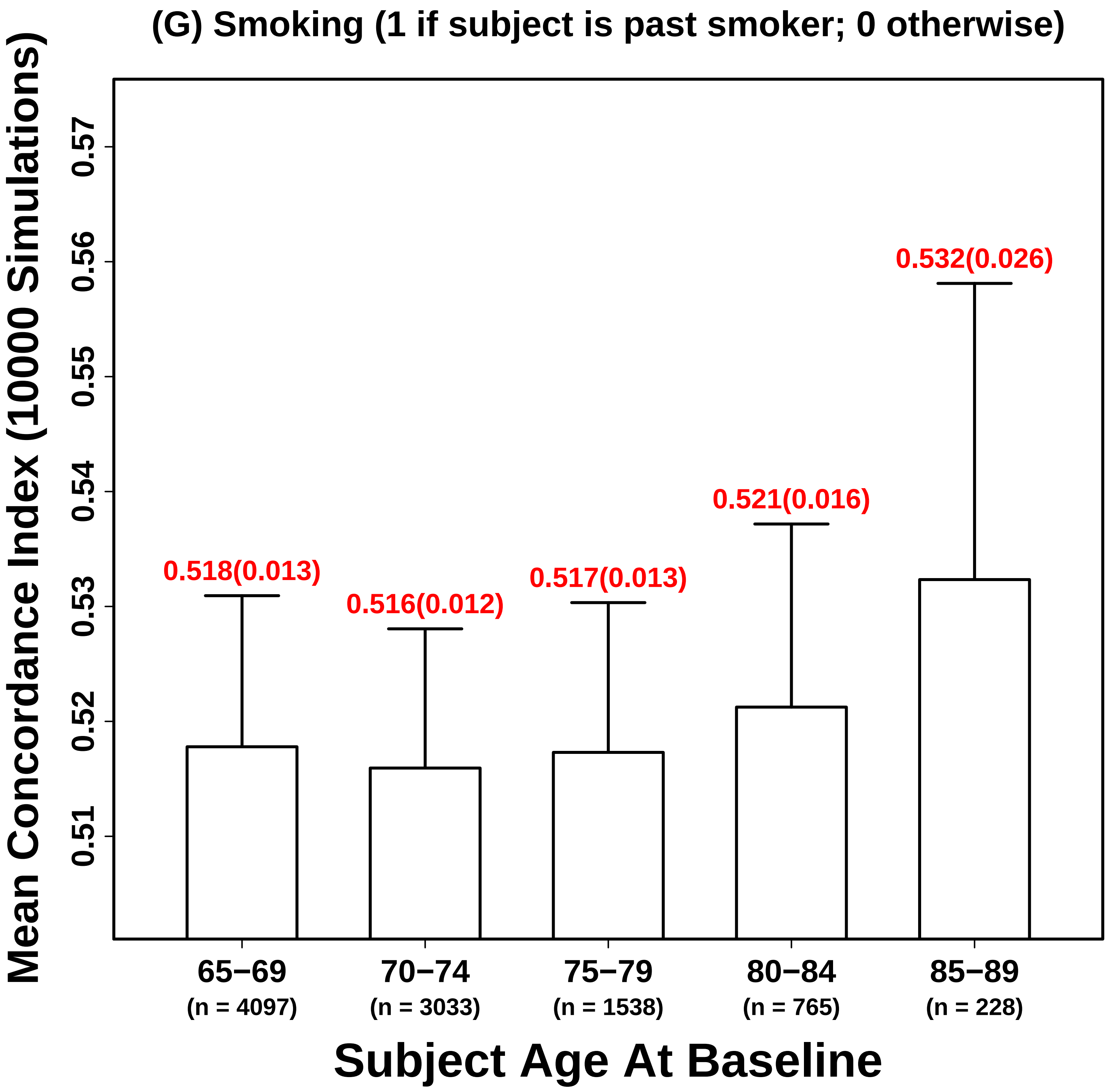
(G) Smoking (1 if subject is past smoker; 0 otherwise)
Mean Concordance Index (10000 Simulations)
0.51
0.52
0.53
0.54
0.55
0.56
0.57
0.518(0.013)
0.516(0.012)
0.517(0.013)
0.521(0.016)
0.532(0.026)
65−69
(n = 4097)
70−74
(n = 3033)
75−79
(n = 1538)
80−84
(n = 765)
85−89
(n = 228)
Subject Age At Baseline

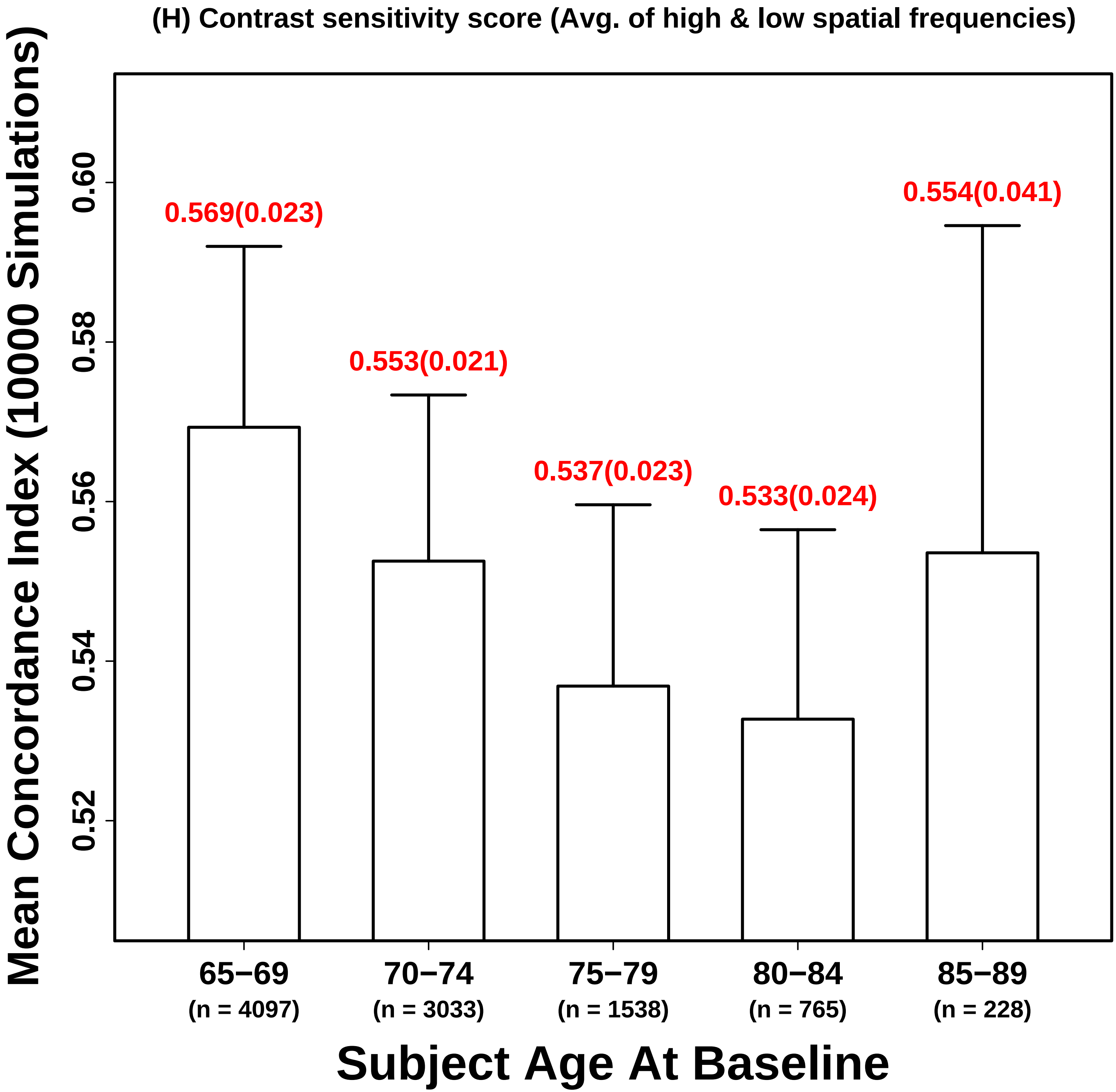
(H) Contrast sensitivity score (Avg. of high & low spatial frequencies)
Mean Concordance Index (10000 Simulations)
0.52
0.54
0.56
0.58
0.60
0.569(0.023)
0.553(0.021)
0.537(0.023)
0.533(0.024)
0.554(0.041)
65−69
(n = 4097)
70−74
(n = 3033)
75−79
(n = 1538)
80−84
(n = 765)
85−89
(n = 228)
Subject Age At Baseline

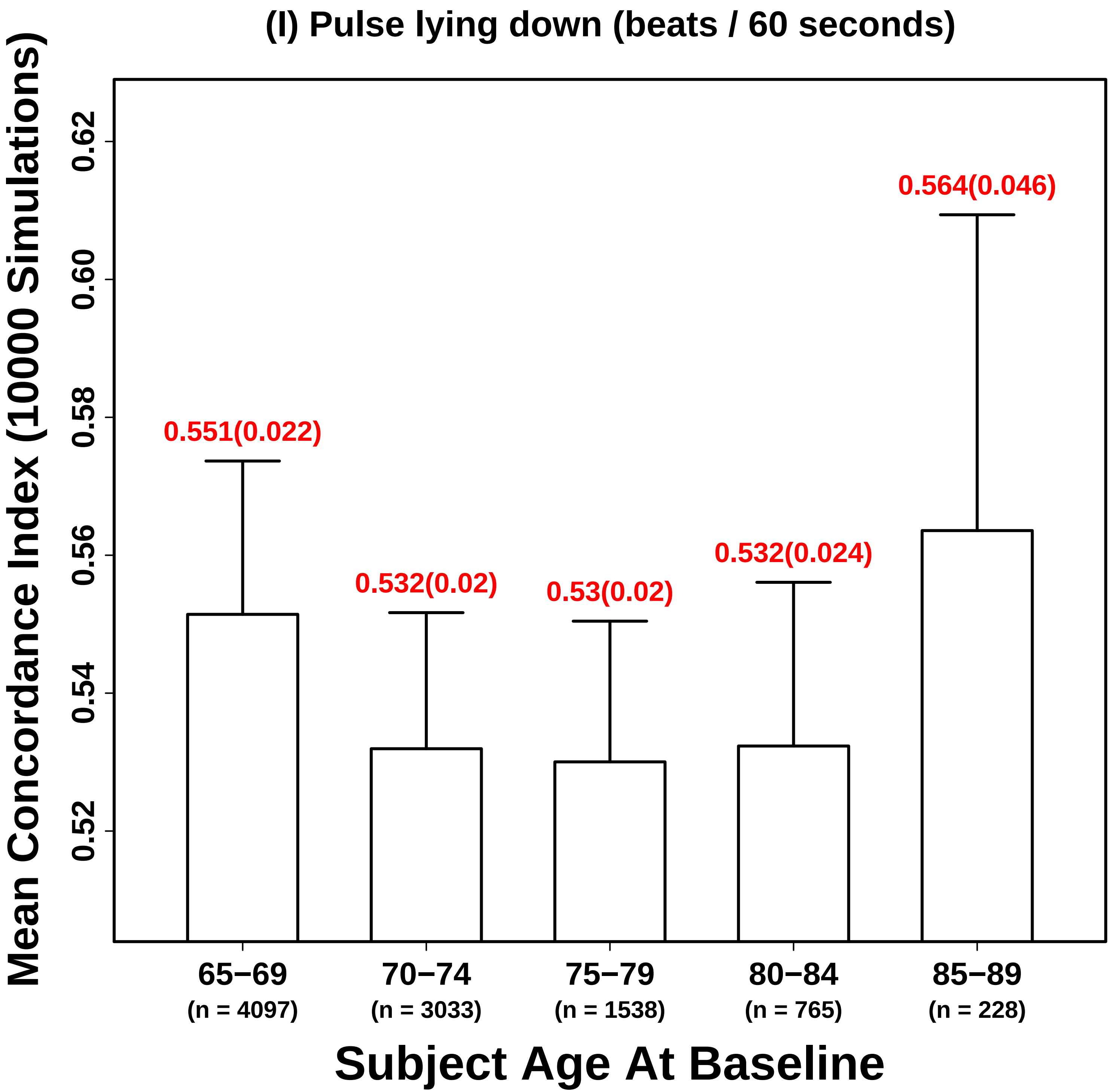
(I) Pulse lying down (beats / 60 seconds)
Mean Concordance Index (10000 Simulations)
0.52
0.54
0.56
0.58
0.60
0.62
0.551(0.022)
0.532(0.02)
0.53(0.02)
0.532(0.024)
0.564(0.046)
65−69
(n = 4097)
70−74
(n = 3033)
75−79
(n = 1538)
80−84
(n = 765)
85−89
(n = 228)
Subject Age At Baseline

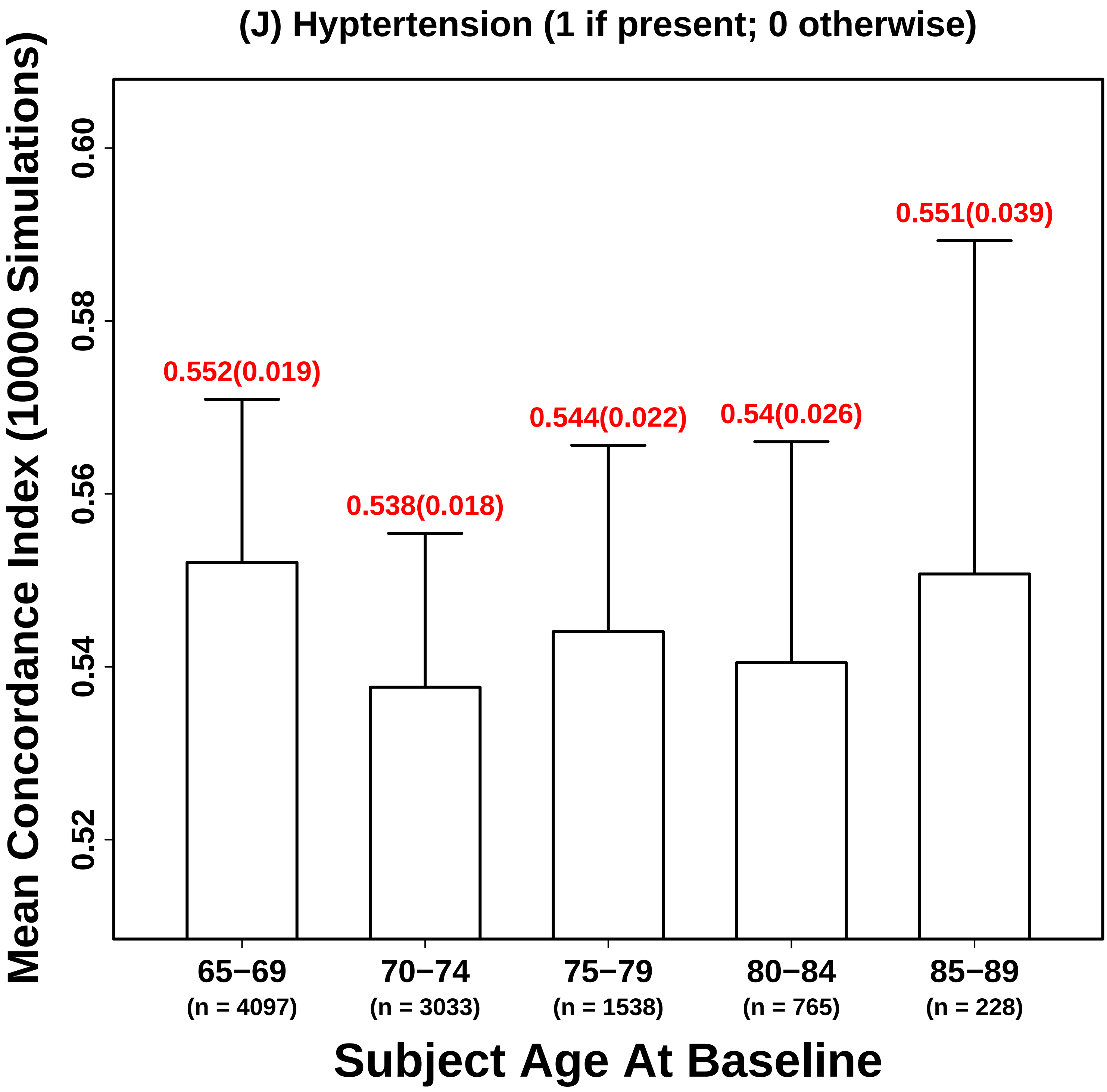
(J) Hyptertension (1 if present; 0 otherwise)
Mean Concordance Index (10000 Simulations)
0.52
0.54
0.56
0.58
0.60
0.552(0.019)
0.538(0.018)
0.544(0.022)
0.54(0.026)
0.551(0.039)
65−69
(n = 4097)
70−74
(n = 3033)
75−79
(n = 1538)
80−84
(n = 765)
85−89
(n = 228)
Subject Age At Baseline

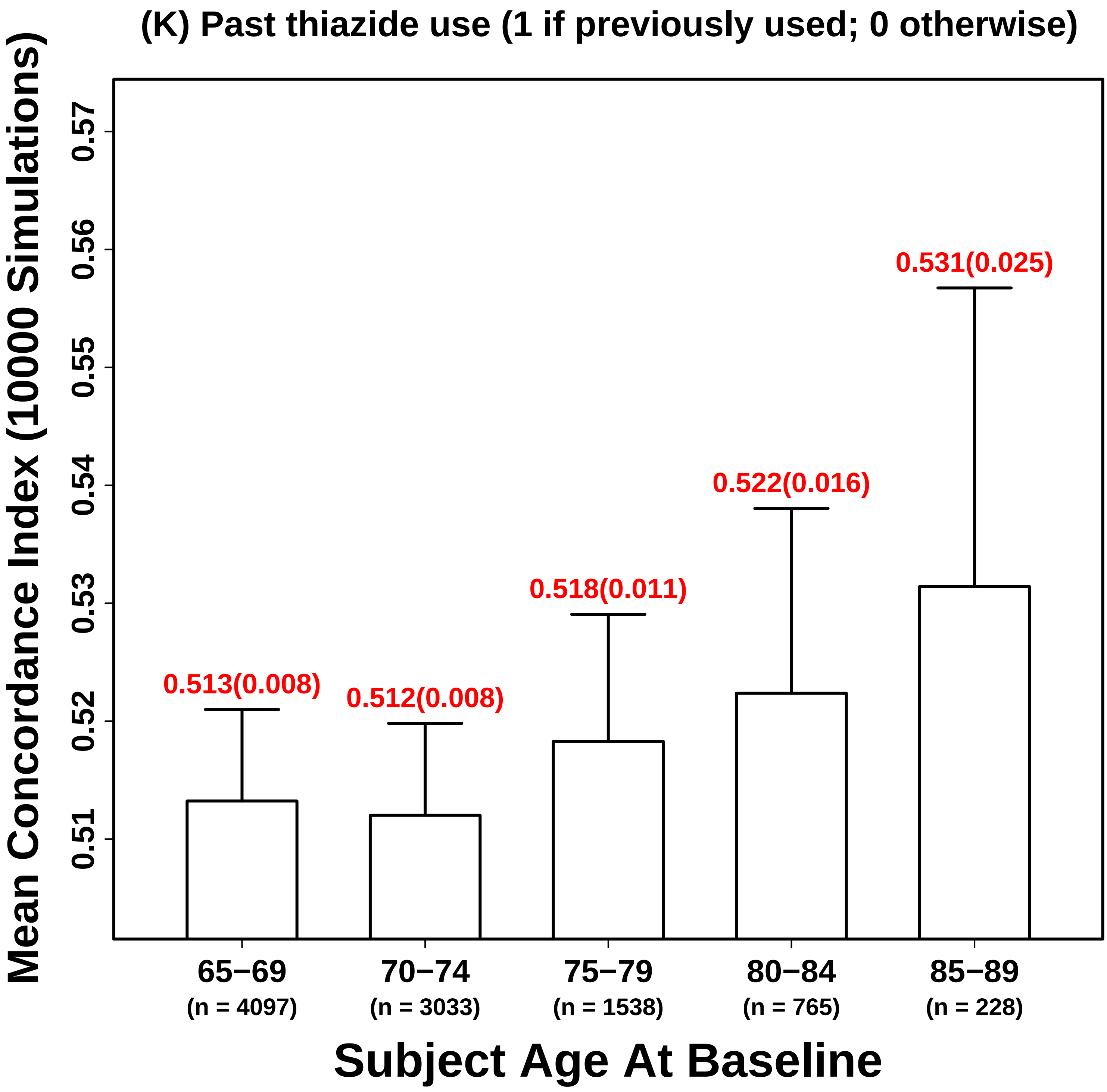
(K) Past thiazide use (1 if previously used; 0 otherwise)
Mean Concordance Index (10000 Simulations)
0.51
0.52
0.53
0.54
0.55
0.56
0.57
0.513(0.008)
0.512(0.008)
0.518(0.011)
0.522(0.016)
0.531(0.025)
65−69
(n = 4097)
70−74
(n = 3033)
75−79
(n = 1538)
80−84
(n = 765)
85−89
(n = 228)
Subject Age At Baseline

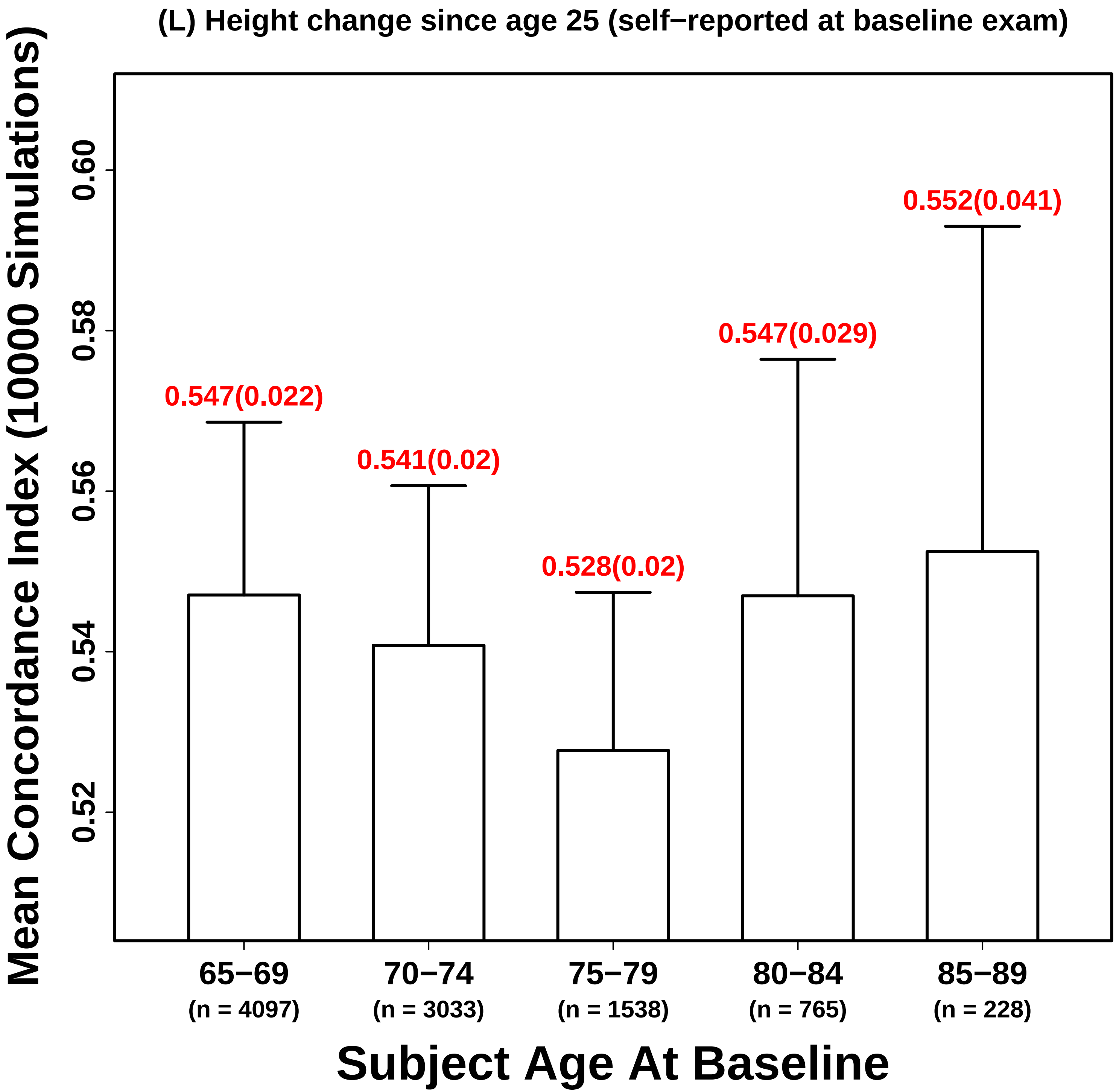
(L) Height change since age 25 (self−reported at baseline exam)
Mean Concordance Index (10000 Simulations)
0.52
0.54
0.56
0.58
0.60
0.547(0.022)
0.541(0.02)
0.528(0.02)
0.547(0.029)
0.552(0.041)
65−69
(n = 4097)
70−74
(n = 3033)
75−79
(n = 1538)
80−84
(n = 765)
85−89
(n = 228)
Subject Age At Baseline

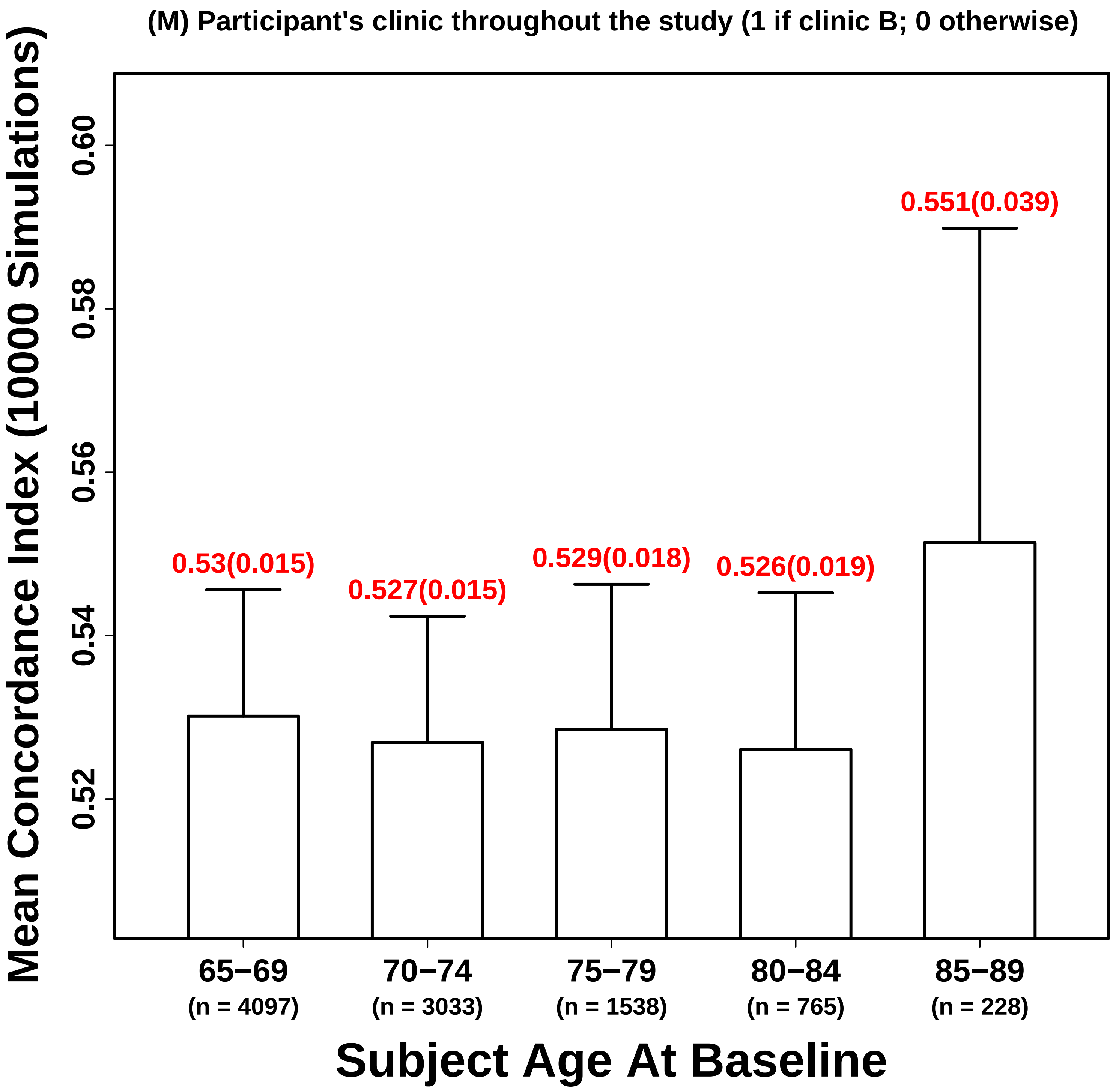
(M) Participant's clinic throughout the study (1 if clinic B; 0 otherwise)
Mean Concordance Index (10000 Simulations)
0.60
0.58
0.56
0.54
0.52
0.53(0.015)
0.527(0.015)
0.529(0.018)
0.526(0.019)
0.551(0.039)
65−69
(n = 4097)
70−74
(n = 3033)
75−79
(n = 1538)
80−84
(n = 765)
85−89
(n = 228)
Subject Age At Baseline

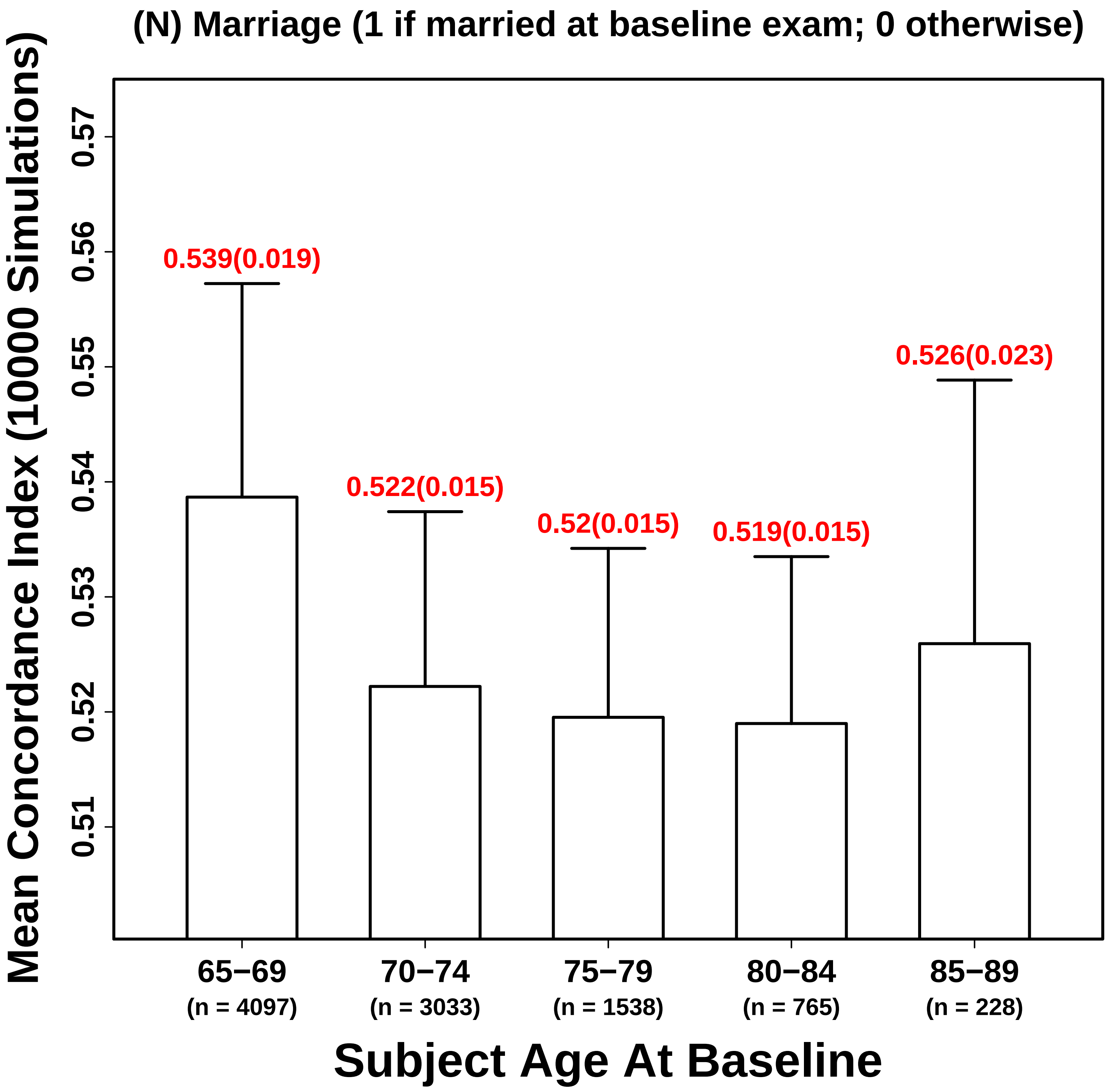
(N) Marriage (1 if married at baseline exam; 0 otherwise)
Mean Concordance Index (10000 Simulations)
0.57
0.56
0.55
0.54
0.53
0.52
0.51
0.539(0.019)
0.522(0.015)
0.52(0.015)
0.519(0.015)
0.526(0.023)
65−69
(n = 4097)
70−74
(n = 3033)
75−79
(n = 1538)
80−84
(n = 765)
85−89
(n = 228)
Subject Age At Baseline
