# Supplementary material for: Indicators of "Healthy Aging" in older women (65-69 years of age). A data-mining approach based on prediction of long-term survival
Source: BMC Geriatr. 2010 Aug 17;10:55. doi: 10.1186/1471-2318-10-55 (PMC2936300; doi:10.1186/1471-2318-10-55)
Supplement: Additional file 5 — Random Variable Selection as a Modeling Strategy. Previous investigations have suggested that exact specification of variables is not always an important factor determining the performance of a prognostic model. This file therefore contains analyses that compare prognostic performance of the 13-variable model generated in our study (see Table 2) with the performance of 13-variable models that include variables chosen at random from a pool of variables (either with or without pre-filtering of the variable pool). We note that sensitivity of model performance to variable specification is expected to increase for models that are based upon a smaller number of predictor variables. [file 1471-2318-10-55-S5.PDF]

Additional File 5

Indicators of "Healthy Aging" in Older Women (65-69 years of age). A Data-mining Approach based on Prediction of Long-term Survival.

William R. Swindell, Kristine E. Ensrud, Peggy M. Cawthon, Jane A. Cauley, Steve R. Cummings, Richard A. Miller

---

Random Variable Selection as a Modeling Strategy

Previous investigations have suggested that exact selection of variables is not of key importance in determining the performance of a prognostic model. In some cohorts, for example, high-quality models can be generated when component variables appropriately reflect the accumulation of "deficits", where the exact choice of deficits has little influence on model performance. This approach has proven especially useful within the context of frailty, and has led to the formation of indices able to predict long-term as well as short-term survival (see: Kulminski et al. 2008, J. Gerontol. Biol. Sci. 63A: 1053-1059; Rockwood et al. 2006, J. Am. Geriatr. Soc. 54:975-979).

This file contains analyses that compare prognostic performance of the 13-variable model generated in our study (see Table 2) with the performance of 13-variable models that include variables chosen at random (see Figures A - D on following page). We first evaluated the performance of the 13-variable model identified in our analysis (Table 2), using 10,000 cross-validation simulations, which yielded an average concordance index (*C*) of 0.673 (SD = 0.019) among the 10,000 simulations. The empirical distribution of *C* generated from these 10,000 simulations conducted in this analysis is shown as the black distribution in Figures A - D (see page 2 of this file).

We next repeated the same procedure, with risk scores generated from models that included a set of 13 variables chosen at random, at each iteration, from the 377 included in our study (see red distribution in Figure A). The procedure was then repeated, with variables randomly chosen from a filtered pool that included only the top 200 variables (red distribution in Figure B), a pool of the top 100 variables (red distribution in Figure C), or a pool of the top 50 variables (red distribution in Figure D). In each case, the "top variables" were established based upon a ranking of variables according to their predictive performance in univariate models. These analyses provide an indication of the benefit associated with our variable selection strategy and resultant 13-variable model, relative to an alternative strategy in which variables are chosen randomly (from either a filtered or non-filtered pool). A table that summarizes results from these analyses is provided on page 3 of this file.

Contact: William R. Swindell, wswindel@umich.edu

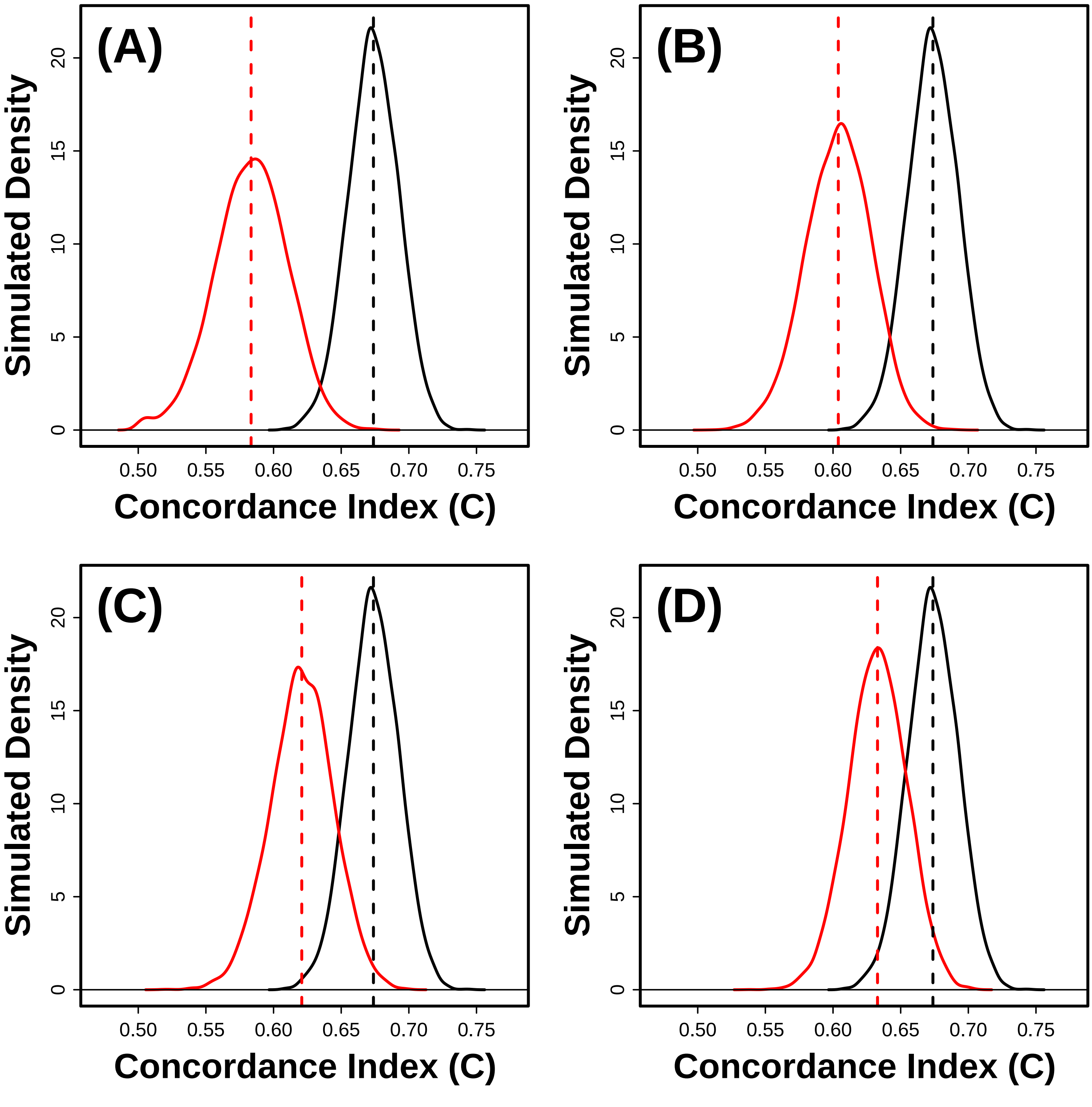
(A)
(B)
(C)
(D)
Simulated Density
Concordance Index (C)
0
5
10
15
20
0.50
0.55
0.60
0.65
0.70
0.75
