# Supplementary material for: Indicators of "Healthy Aging" in older women (65-69 years of age). A data-mining approach based on prediction of long-term survival
Source: BMC Geriatr. 2010 Aug 17;10:55. doi: 10.1186/1471-2318-10-55 (PMC2936300; doi:10.1186/1471-2318-10-55)
Supplement: Additional file 6 — Comparison of model performance to an index that quantifies the accumulation of deficits or health problems. Previous studies have shown that high-quality predictive models can be generated based upon an index that appropriately reflects the accumulation of health problems or "deficits". Such deficits should exhibit greater frequency with age, be indicative of health status, but should not be universally present among older subjects. Examples of such deficits include the presence of depression, weight loss, difficulty with daily living tasks and a history of heart disease. This file generates an "SOF Deficit Index" and compares the performance of this index to that of the 13-variable healthy aging index presented in Table 2. The comparison is made with respect to the young SOF subjects that are the main focus of this paper and also with respect to older SOF cohorts aged 70-74 (n = 3033), 75-79 (n = 1538), 80-84 (n = 765) and 85-89 (n = 228). [file 1471-2318-10-55-S6.PDF]

Additional File 6

Indicators of "Healthy Aging" in Older Women (65-69 years of age). A Data-mining Approach based on Prediction of Long-term Survival.

William R. Swindell, Kristine E. Ensrud, Peggy M. Cawthon, Jane A. Cauley, Steve R. Cummings, Richard A. Miller

---

Evaluation of SOF Deficit Index and Comparison to Healthy Aging Index

(1) Introduction

Previous studies have shown that indices designed to quantify the accumulation of health problems or "deficits" in older individuals can provide powerful prognostic tools that predict both short and long-term survival (e.g., Searle et al. 2008, BMC Geriatrics 8: 24). It was therefore of interest to compare the prognostic performance of the 13-variable healthy aging index we developed (see Table 2 of manuscript file) with an alternative approach that is based upon accumulation of deficits.

An "SOF deficit index" was constructed following guidelines proposed by Searle et al. (2008). While it has been shown that exact choice of deficits is often not a critical determinant of prognostic value, we nonetheless attempted to construct an index for which the deficit composition approximately matched that of the index presented by Searle et al. (2008). In this previous study, a 40-variable index is presented, which includes variables related to the ability of subjects to perform daily living tasks (e.g., bathing, eating and grooming), indicators of recent health status (e.g., has a subject lost more the 10 lbs in last year?), variables related to mental health outlook or mental performance (e.g., how often a subject "feels happy" or "feels depressed", MMSE score), variables related to disease history (e.g., cancer), and measures of physical performance (e.g., walking speed or grip strength), along with body mass index and peak blood flow (see Table 1 from Searle et al. 2008).

The SOF deficit index we constructed resembles that proposed by Searle et al. (2008) in many respects, although some disparities were unavoidable because our analysis considers a different dataset (see Table A). We included variables related to the ability of subjects to perform daily living tasks (e.g., ability to do shopping), recent health history (e.g., health change within the last 12 months), disease history (e.g., diabetes, stroke), physical performance (e.g., grip strength), MMSE score and body mass index (Table A). With respect to each category of measurement, we attempted to match the index used by Searle et al. (2008) in terms of the number of variables drawn from each category, with the aim of generating an index that assigned a comparable degree of weight to particular deficit categories. Despite these efforts, the SOF deficit index we generated differs from that proposed by Searle et al. (2008) in certain respects. For instance, our

SOF deficit index does not include a measure of peak flow. Additionally, the baseline SOF data we consider includes few indicators of mental health outlook, although as proxy measures, we have considered whether subjects report taking anti-depressant or anti-anxiety medications (Table A).

The deficit index approach advocated by Searle et al. (2008) involves assigning each deficit a score between 0 and 1 for each subject, where 0 corresponds to absence of a given deficit and 1 corresponds to the full expression of the deficit. For categorical measures, a deficit may be assigned 0-1 coding, while continuous deficit variables may have more than two levels (e.g., 0, 0.50, 1). We have followed this practice in the construction of our SOF deficit index (Table A). For continuous variables, we formed discrete levels that either corresponded to those used by Searle et al. (2008), or were determined using the quantitative approach suggested by Searle et al. (2008), which is based upon the relationship between an "intermediate deficit index" and the continuous variable under consideration. The final deficit index was generated by calculating the average score for each subject with respect to the 40 deficits included in our index (Searle et al. 2008).

(2) <u>Evaluation of Deficit Inclusion Criteria</u>

Searle et al. (2008) described criteria that a candidate deficit should meet to warrant inclusion within a deficit index. These criteria are that (1) a deficit should be associated with health status to some degree, (2) a deficit should increase in prevalence with age among subjects, and that (3) a deficit should not "saturate" at an early age and become universal among older subjects. We evaluated whether variables included in our SOF deficit index (Table A) satisfied these criteria. A small number of variables did not satisfy all criteria, and for our evaluation of index performance, we evaluate performance both with and without variables that might be excluded on the basis of one of the three criteria.

(2.1) Association of deficits with health status

The association of deficit variables with health status was evaluated using two criteria (Tables B and C). First, we determined whether deficit variables were associated with a subject's response to the question "How is my health compared to others my age?" (Table B). All variables were significantly associated with response to this question ($P < 0.004$; chi-square test; see Table B), with the exception of grip strength (GRPAVG), which was not significantly associated with this self-assessment of individual health ($P = 0.954$). As a second criterion, we determined whether each deficit variable was a significant predictor of survival as a single variable in a univariate Cox regression model (Table C). These analyses indicated that nearly all variables were significant predictors of survival, providing further evidence of an association between the selected variables and health status. Of the 40 deficit variables, 36 variables were associated with hazard ratio estimates significantly greater than one. Variables not significantly associated with survival included CMP12 ($P = 0.813$), ANTDEP ($P = 0.133$), and GRPAVG ($P = 0.357$). BMI also was not significantly associated with survival, although this variable was marginally significant ($P = 0.088$) and the estimated hazard ratio was greater than one ($HR = 1.065$; see Table C).

(2.2) Association of deficits with subject age

Logistic regression models were used to determine whether the frequency of each deficit increased with age (Table D). For deficit variables with more than two levels, upper levels of each variable were combined to create binary 0-1 variables, with 0 representing absence of deficit and 1 representing expression of deficit (Table D). The binary 0-1 variable created in this fashion was then treated as the dependent variable in logistic regression analyses. This demonstrated that, for nearly all deficit variables, the deficit frequency tended to increase with age (i.e., logistic regression analysis yielded a positive slope estimate with respect to age; exceptions were COMP, ANTDEP and ANTANX; see Table D), with a significant age-related increase in deficit frequency observed for 33 of the 40 variables ($P \leq 0.042$).

(2.3) Variability of deficits with increasing age

To ensure that deficit variables did not "saturate with age", with uniform scores assigned to older subjects, we evaluated the Gini Index of each deficit variable with respect to subjects from five SOF age cohorts (i.e., ages 65-69, 70-74, 75-79, 80-84 and 85-89; see Table E). For a categorical variable with n classes, in which the respective class frequencies are $p_1, p_2, \ldots, p_n$, the Gini Index is defined as $1- (p_1^2 + p_2^2 + \ldots + p_n^2)$. This Index thus provides a measure of variability for categorical variables, and ranges in value between 0 and 1, with 0 representing complete uniformity (saturation of a given deficit with respect to a certain group of subjects) and 1 representing maximal heterogeneity (i.e., subjects within a cohort vary substantially with respect to a given deficit measure) (for a review of this variability measure, see Tan, Steinbach and Kumar, *An Introduction to Dating Mining*, Addison Wesley, 2005). This analysis demonstrated that, with respect to each cohort, there was some heterogeneity among subjects with respect to each deficit variable (i.e., Gini Index scores were non-zero), and that this heterogeneity did not diminish entirely with increasing age. Deficits included in our SOF index, therefore, tended to increase with age, but such deficits did not become universally present among older subjects (see Table E).

Deficits included in our SOF deficit index were collectively associated with a broad range of subject characteristics that touch upon several aspects of an aging adult (a criteria specified by Searle et al. 2008). The final deficit score was calculated, for each subject, by averaging values assigned to each of the 40 deficit variables (Table A). The index generated in this fashion exhibited an association with survival that was significant and much stronger than that observed with respect to any of the 40 component deficits taken individually ($HR = 83.4$; $P < 0.001$; see Table C). Among all 9704 SOF subjects, the distribution of the deficit index was well-approximated by a gamma distribution (Figure A), which is consistent with deficit indices that have been constructed based upon different datasets in previous studies (e.g., Searle et al. 2008). Additionally, there was a strong and significant linear association between the deficit index and subject age at baseline evaluation, indicating that deficits contributing to the deficit index tended to accumulate with age (see Figure B).

(3) Performance of Deficit and Healthy Aging Indices

The ability of the SOF deficit index to predict survivorship patterns was independently evaluated with respect to five SOF age cohorts (ages 65-69, 70-74, 75-79, 80-84 and 85-89). For each cohort, predictive capacity was evaluated using 10-fold cross-validation (see Methods of manuscript) and comparisons were made to performance of the healthy aging index that is described in Table 2 of the manuscript. We consider several implementations of the SOF deficit index (Figures C – F). In particular, performance of the SOF deficit index is evaluated as a single variable in a Cox univariate regression model, and also as one variable in a bivariate Cox model that also includes subject age as a second predictor. Additionally, the SOF deficit index is evaluated both with and without variables that were questionable with respect to inclusion criteria outlined by Searle et al. (2008).

(3.1) Univariate Cox Model with (40-variable) deficit index as single predictor

The ability of the deficit index to predict survivorship patterns was first evaluated based upon univariate Cox models in which the (40-variable) deficit index was the only predictor variable (Figure C). In figure C, the height of each bar represents, for each of five age cohorts, the average concordance score among 10,000 cross validation trials, and error bars correspond to the standard deviation among the 10,000 concordance scores obtained from the cross-validation procedure (standard errors are approximately ± 0.001). This analysis revealed that, with respect to each age cohort, the SOF deficit index yielded mean concordance scores that significantly exceeded the random expectation (mean concordance of 0.500), with mean concordance estimates ranging between 0.569 and 0.630 (Figure C). With respect to the youngest SOF cohort (ages 65-69), the healthy aging index exhibited stronger predictive performance (mean concordance of 0.673 versus 0.605). However, the advantage of the healthy aging index declined with respect to increasingly older SOF cohorts, such that among subjects aged 85-89, performance of the healthy aging index was comparable to that of the deficit index (mean concordance of 0.584 and 0.585 for the healthy aging and deficit indices, respectively; see Figure C).

(3.2) Bivariate Cox Model with (40-variable) deficit index and age as predictors

Previous investigations have evaluated deficit indices in the context of bivariate Cox models that include both deficit index score and subject age as predictor variables (e.g., Searle et al. 2008). The above analyses were therefore repeated using this alternative strategy (Figure D). In this analysis, predictive performance of the deficit index (with age as a covariate) exceeded performance of the healthy aging index with respect to the oldest SOF cohort (mean C of 0.593 for deficit index compared to 0.585 for healthy aging index; see Figure D).

(3.3) Univariate Cox Model with (35-variable) deficit index as single predictor

The analysis presented in Figure C is based upon a 40-variable deficit index designed to closely approximate the deficit index presented by Searle et al. (2008). However, this deficit index includes some variables that were questionable with respect to the inclusion criteria described by Searle et al. (2008), at least with regard to the SOF subjects evaluated in our investigation (see

Tables B – E). We therefore repeated the analyses presented in Figure C but instead evaluated a 35-variable deficit index in which five variables were removed on the basis of the inclusion criteria described by Searle et al. (2008). The excluded variables were GRPAVG (not significantly associated with health status or survivorship; see Tables B and C), CMP12 (estimated hazard ratio was less than one and also non-significant, suggesting poor association with health status; see Table C), COMP (the deficit frequency decreased with age on average; see Table D), ANTDEP (the deficit frequency decreased with age on average; see Table D) and ANXMED (the deficit frequency decreased with age on average; see Table D). The removal of these variables, however, generated a 35-variable deficit index that was still correlated strongly with the 40-variable deficit index, and overall, predictive performance was not greatly altered (compare Figures C and E). The largest difference between Figures C and E relates to the age 80-84 SOF cohort. With respect to this age group, the mean concordance estimate of the 35-variable index (mean C = 0.573; Figure E) was slightly greater than that of the 40-variable index (mean C = 0.569; Figure C).

(3.4) Bivariate Cox model with (35-variable) deficit index and age as predictors

The analyses presented in Figure D were repeated (bivariate Cox models with the deficit index and subject age as predictor variables). However, the deficit index evaluated was based upon only 35 deficit variables that remained after excluding five deficit variables (GRPAVG, CMP12, COMP, ANTDEP, ANXMED; see section 3.3 above) that were questionable with respect to the inclusion criteria evaluated in Tables B – E. The exclusion of these five variables, however, had a negligible impact on index performance, and overall the 35-variable index and 40-variable index yielded very similar performance (compared Figures D and F). The largest difference between the 35-variable and 40-variable indices was observed with respect to the age 80-84 SOF cohort. With respect to this age group, the mean concordance estimate associated with the 35-variable index (mean C = 0.582; Figure F) was slightly greater than that associated with the 40-variable index (mean C = 0.578; Figure D).

(4) <u>Summary</u>

Results presented in this file demonstrate that a deficit index approach (Searle et al. 2008; BMC Geriatrics 24:8) provides a flexible strategy for generating a model that yields predictions more accurate than expected on the basis of chance alone (0.569 $\leq$ mean $C \leq$ 0.617). Among younger SOF subjects, the 13-variable healthy aging index exhibits stronger predictive performance (e.g., mean C of 0.674 versus 0.617 among SOF subjects aged 65-69; see Figure F). However, among increasingly older SOF subject cohorts, the performance advantage of the healthy aging index progressively declines, with the deficit index approach yielding greater performance among SOF subjects aged 85-89 (e.g., mean C of 0.594 versus 0.584; see Figure F).

Contact: William R. Swindell, wswindel@umich.edu

Table A. List of 40 deficit variables considered in SOF deficit index. The 40 deficit variables were drawn from seven general categories (ability to perform daily living tasks, indicators of recent health, mental outlook, mental performance, disease history, physical performance and body composition). These categories and the variables associated with each category were selected with the aim of establishing correspondence to the 40-variable deficit index proposed by Searle et al. (2008) [BMC Geriatrics 8:24]. The ID listed for each variable corresponds to the identifier associated with each variable in the SOF database, such that the listed IDs can be used to obtain further information online from the Study of Osteoporotic Fractures website (http://sof.ucsf.edu/Interface/). The final column describes the coding that was used for each variable on the interval [0, 1]. Footnotes listed below the table provide further details regarding the approach used to code some deficit variables.

ID	Category	Description	Variable Coding
CKR1	Daily living	Do you have difficulty preparing meals?	1: Yes<br>0: No
HH1	Daily living	Can you do heavy housework?	1: No<br>0: Yes
SH1	Daily living	Can you do shopping?	1: No<br>0: Yes
CLB1	Daily living	Can you climb 10 steps?	1: No<br>0: Yes
STP1	Daily living	Can you walk down 10 steps?	1: No<br>0: Yes
WLK1	Daily living	Can you walk 2-3 blocks?	1: No<br>0: Yes
AUTOC	Daily living	Degree of difficulty getting in/out of automobile	1: Unable<br>0.66: Much<br>0.33: Some<br>0: None
BENDC	Daily living	Degree of difficulty bending down to pick things up	1: Unable<br>0.66: Much<br>0.33: Some<br>0: None
RCHC	Daily living	Degree of difficulty reaching object	1: Unable<br>0.66: Much<br>0.33: Some<br>0: None
SCKONC	Daily living	Degree of difficulty putting socks on	1: Unable<br>0.66: Much<br>0.33: Some<br>0: None
CHR	Daily living	Did subject use arms to stand from chair 5 times?	1: Yes<br>0: No
STPARM	Daily living	Did subject use arms to step up or step down?	1: Yes<br>0: No
AIDS	Daily living	Does subject use walking aids?	1: Yes<br>0: No

CHORHR[1]	Daily living	hours/week doing heavy chores	1: ≤ 9.04 hours<br>0: > 9.04 hours
WTLS50[2]	Recent health	Weight change: 50 to now in kgs?	1: gained ≥ 4.17 kgs<br>0: gained < 4.17 kgs
COMP	Recent health	Health compared to others your age	1: Very poor<br>0.75: Poor<br>0.50: Fair<br>0.25: Good<br>0: Excellent
CMP12	Recent health	Health compared to 12 months ago	1: Much worse<br>0.75: Somewhat worse<br>0.50: About the same<br>0.25: Somewhat better<br>0: Much better
INBED7	Recent health	More than 7 days in bed in row?	1: Yes<br>0: No
HWKINT	Recent health	Number of times engaged in high intensity exercise last year?	1: at least once<br>0: never
EXER	Recent health	Take walks for exercise?	1: No<br>0: Yes
ANTDEP	Mental outlook	Antidepressant use last 12 months	1: Yes<br>0: No
ANXMED	Mental outlook	Antidepressant use last 12 months	1: Yes<br>0: No
CAROUS	Mental outlook	How often leave neighborhood?	1: < once / month<br>0.80: 2-3 days / month<br>0.60: once / week<br>0.40: several times / week<br>0.20: once / day<br>0: several times / day
SHT3MS	Mental performance	Short mini-mental status exam	1: <10<br>0.75: 11–17<br>0.50: 18–20<br>0.25: 20–24<br>0: >24
HYTEN	Disease history	Does subject have hypertension?	1: Yes<br>0: No
EARTH	Disease history	Dr ever told you that you have arthritis	1: Yes<br>0: No
EDIAB	Disease history	Dr ever told you that you have diabetes	1: Yes<br>0: No
EHIP	Disease history	Hip pain for most days in a month	1: Yes<br>0: No
EHTHY	Disease history	doctor ever told you have high thyroid?	1: Yes<br>0: No

EOSTEO	Disease history	doctor ever told you have osteoporosis?	1: Yes<br>0: No
EPARK	Disease history	doctor ever told you have Parkinson's?	1: Yes<br>0: No
ESTRK	Disease history	doctor ever told you have had a stroke?	1: Yes<br>0: No
HSP	Disease history	Have you been a patient in a hospital, past 12 months?	1: Yes<br>0: No
BCANC	Disease history	Have you ever had breast cancer	1: Yes<br>0: No
SLPMED	Disease history	Have you used sleep medications in past 12 months	1: Yes<br>0: No
GRPAVG[3]	Physical performance	avg of right and left grip strength (kg)	If BMI ≤ 23,<br>1: ≤ 17 kg<br>0: > 17 kg<br><br>If BMI 23.1–26,<br>1: ≤ 17.3 kg<br>0: > 17.3 kg<br><br>If BMI 26.1-29,<br>1: ≤ 18 kg<br>0: > 18 kg<br><br>If BMI > 29,<br>1: ≤ 21 kg<br>0: > 21 kg
WLKSPD[4]	Physical performance	walking speed usual pace (m/s)	1: ≤ 1.08 m/s<br>0: > 1.08 m/s
TWTM[5]	Physical performance	secs to complete tandem walk course	1: ≥ 10.16 sec<br>0: < 10.16 sec
HRFC[6]	Physical performance	avg hip abduct force rt side (kg)	1: ≤ 11.21 kg<br>0: > 11.21 kg
BMI[7]	Body composition	Body mass index	1: BMI <18.5 or ≥ 30<br>0.50: BMI 25 - 30<br>0: BMI 18.5 - 25

[1]CHORHR: The cut point for this variable was chosen based upon the "interim frailty index method" described by Searle et al. (2008) [BMC Geriatrics 8:24]. A deficit was assigned for variable values for which, on average, individuals had an interim frailty index larger than 0.20 (see page 3 from Searle et al. (2008)).
[2]WTLS50: The cut point for this variable was chosen based upon the "interim frailty index method" described by Searle et al. (2008) [BMC Geriatrics 8:24]. A deficit was assigned for variable values for which, on average, individuals had an interim frailty index larger than 0.20 (see page 3 from Searle et al. (2008)).
[3]GRPAVG: The cut point for this variable was chosen to be consistent with Searle et al. (2008) [BMC Geriatrics 8:24]. The cut point was chosen by Searle et al. (2008) based upon results

presented by Gill et al. (2006) [Arch Intern Med 166:418-423] and Fried et al. (2001) [J Gerontol A Biol Sci Med Sci 56A:M146-M156].

[4]WLKSPD: The cut point for this variable was chosen based upon the "interim frailty index method" described by Searle et al. (2008) [BMC Geriatrics 8:24]. A deficit was assigned for variable values for which, on average, individuals had an interim frailty index larger than 0.20 (see page 3 from Searle et al. (2008)).

[5]TWTM: The cut point for this variable was chosen based upon the "interim frailty index method" described by Searle et al. (2008) [BMC Geriatrics 8:24]. A deficit was assigned for variable values for which, on average, individuals had an interim frailty index larger than 0.20 (see page 3 from Searle et al. (2008)).

[6]HRFC: The cut point for this variable was chosen based upon the "interim frailty index method" described by Searle et al. (2008) [BMC Geriatrics 8:24]. A deficit was assigned for variable values for which, on average, individuals had an interim frailty index larger than 0.20 (see page 3 from Searle et al. (2008)).

[7]BMI: The cut point for this variable was chosen to be consistent with Searle et al. (2008) [BMC Geriatrics 8:24]. The cut point was chosen by Searle et al. (2008) based upon results presented by Flegal et al. (2007) [JAMA 298:2028-2037]

Table B. Association of deficit variables with baseline health status. Variables included as components within a deficit index should be associated with health status of individuals (Searle et al. 2008, BMC Geriatrics 8:24). To determine if this was the case for variables listed in Table A, we evaluated each deficit variable to determine whether it was significantly associated with the response of subjects to the question "How is my health compared to others my age?", where the response of subjects to this question was scored categorically on a five-point scale (very poor, poor, fair, good, excellent). For each deficit variable, a chi-square test was used to determine if the variable was significantly associated with the response of subjects to this question (among all SOF subjects aged 65-89, n = 9704). In the table below, the chi-square statistic and p-value associated with each variable is listed. Variables for which the association with health status was questionable are displayed in red font and were excluded from analyses presented in Figures E and F. The final row lists the chi-square statistic and p-value associated with a deficit index that is based upon all 40 variables.

Variable	Chi-Square (P-Value)
CKR1	519.966 (< 0.001)
HH1	975.849 (< 0.001)
SH1	766.991 (< 0.001)
CLB1	807.725 (< 0.001)
STP1	440.382 (< 0.001)
WLK1	833.841 (< 0.001)
AUTOC	634.345 (< 0.001)
BENDC	678.189 (< 0.001)
RCHC	746.033 (< 0.001)
SCKONC	478.481 (< 0.001)
CHR	285.504 (< 0.001)
STPARM	325.246 (< 0.001)
AIDS	228.686 (< 0.001)
CHORHR	27.263 (< 0.001)
WTLS50	19.081 (< 0.001)
CMP12	1469.551 (< 0.001)
INBED7	140.412 (< 0.001)
HWKINT	91.206 (< 0.001)
EXER	153.997 (< 0.001)
ANTDEP	107.904 (< 0.001)
ANXMED	205.377 (< 0.001)
CAROUS	469.858 (< 0.001)
SHT3MS	114.746 (< 0.001)
HYTEN	166.374 (< 0.001)
EARTH	334.527 (< 0.001)
EDIAB	189.058 (< 0.001)
EHIP	287.806 (< 0.001)
EHTHY	39.835 (< 0.001)
EOSTEO	244.167 (< 0.001)
EPARK	31.451 (< 0.001)
ESTRK	122.738 (< 0.001)

HSP	168.333 (< 0.001)
BCANC	15.529 (0.004)
SLPMED	117.805 (< 0.001)
GRPAVG	0.682 (0.954)
WLKSPD	419.365 (< 0.001)
TWTM	45.64 (< 0.001)
HRFC	51.48 (< 0.001)
BMI	191.286 (< 0.001)
Deficit Index (Based on 40 Variables)	16425.777 (< 0.001)

Table C. Association of deficit variables with subject survivorship (surrogate measure of health status). Variables included as components within a deficit index should be associated with health status of individuals (Searle et al. 2008, BMC Geriatrics 8:24). This association is likely to exist if the variable is a predictor of survival and is a significant variable within a Cox regression model, with a hazard ratio estimate that is larger than one. In accordance with this reasoning, a Cox regression analysis was carried out for each of the 40 deficit variables listed in Table A (based upon survival patterns among all SOF subjects aged 65-89, *n* = 9704). The table below lists each deficit variable along with the estimated hazard ratio and p-value. Variables for which the association with survival was questionable (p-values that are not at least marginally significant) are displayed in red font and were excluded from analyses presented in Figures E and F. The final row lists the hazard ratio and p-value associated with a deficit index that is based upon all 40 variables.

Variable ID	Hazard Ratio (P-value)
CKR1	2.35(< 0.001)
HH1	1.603(< 0.001)
SH1	2.094(< 0.001)
CLB1	1.874(< 0.001)
STP1	1.692(< 0.001)
WLK1	2.097(< 0.001)
AUTOC	2.383(< 0.001)
BENDC	2.08(< 0.001)
RCHC	2.408(< 0.001)
SCKONC	2.102(< 0.001)
CHR	5.484(< 0.001)
STPARM	2.686(< 0.001)
AIDS	2.302(< 0.001)
CHORHR	1.243(< 0.001)
WTLS50	1.299(< 0.001)
COMP	3.585(< 0.001)
CMP12	0.98(0.813)
INBED7	1.386(< 0.001)
HWKINT	1.411(< 0.001)
EXER	1.405(< 0.001)
ANTDEP	1.118(0.133)
ANXMED	1.091(0.014)
CAROUS	2.999(< 0.001)
SHT3MS	3.66(< 0.001)
HYTEN	1.621(< 0.001)
EARTH	1.124(< 0.001)
EDIAB	1.952(< 0.001)
EHIP	1.071(0.019)
EHTHY	1.283(< 0.001)
EOSTEO	1.266(< 0.001)
EPARK	1.766(< 0.001)
ESTRK	2.09(< 0.001)

HSP	1.489(< 0.001)
BCANC	1.4(< 0.001)
SLPMED	1.173(< 0.001)
GRPAVG	1.026(0.357)
WLKSPD	1.826(< 0.001)
TWTM	1.17(< 0.001)
HRFC	1.568(< 0.001)
BMI	1.065(0.088)
Deficit index (based upon 40 variables)	83.407(< 0.001)

Table D. Association of deficit variables with the baseline age of subjects. Deficits included as components within a deficit index should exhibit increased frequency with age (Searle et al. 2008, BMC Geriatrics 8:24). To determine if this was the case for variables listed in Table A, we used logistic regression analysis to determine whether the frequency of each deficit was positively associated with subject age at baseline examination (including all SOF subjects aged 65-89, *n* = 9704). For binary 0-1 variables, this analysis was performed using the deficit variable directly (without modification). For deficit variables with more than two levels (e.g., 0, 0.50, and 1), the upper levels of the deficit variable were re-coded (for the purpose of this analysis) to create a binary 0-1 deficit variable. For each variable, results from this analysis are listed in the table below, where positive coefficients indicate that a deficit exhibited increased frequency with age (on average) among all subjects within the SOF cohort (ages 65 – 89). We note that inferences concerning age-associated patterns based upon cross-sectional analyses should be interpreted with caution and that this is a limitation of the analyses presented below. Variables for which the association with subject age was questionable (negative coefficient estimates) are displayed in red font and were excluded from analyses presented in Figures E and F.

Variable ID	Coefficient Estimate (SE)	P-Value
CKR1	0.093(0.011)	< 0.001
HH1	0.044(0.004)	< 0.001
SH1	0.091(0.007)	< 0.001
CLB1	0.067(0.005)	< 0.001
STP1	0.065(0.005)	< 0.001
WLK1	0.084(0.005)	< 0.001
AUTOC	0.063(0.014)	< 0.001
BENDC	0.036(0.012)	0.003
RCHC	0.071(0.013)	< 0.001
SCKONC	0.029(0.015)	0.042
CHR	0.144(0.01)	< 0.001
STPARM	0.152(0.006)	< 0.001
AIDS	0.126(0.008)	< 0.001
CHORHR	0.046(0.004)	< 0.001
WTLS50	0.052(0.004)	< 0.001
COMP	-0.021(0.016)	0.19
CMP12	0.035(0.006)	< 0.001
INBED7	0.031(0.008)	< 0.001
HWKINT	0.102(0.014)	< 0.001
EXER	0.046(0.004)	< 0.001
ANTDEP	-0.015(0.011)	0.152
ANXMED	-0.01(0.005)	0.053
CAROUS	0.087(0.01)	< 0.001
SHT3MS	0.15(0.017)	< 0.001
HYTEN	0.056(0.004)	< 0.001
EARTH	0.023(0.004)	< 0.001
EDIAB	0.005(0.007)	0.502
EHIP	0.006(0.004)	0.141
EHTHY	0.024(0.006)	< 0.001

EOSTEO	0.031(0.005)	< 0.001
EPARK	0.041(0.023)	0.07
ESTRK	0.056(0.01)	< 0.001
HSP	0.038(0.006)	< 0.001
BCANC	0.033(0.008)	< 0.001
SLPMED	0.024(0.005)	< 0.001
GRPAVG	0.003(0.004)	0.436
WLKSPD	0.11(0.005)	< 0.001
TWTM	0.014(0.004)	< 0.001
HRFC	0.102(0.005)	< 0.001
BMI	0.102(0.005)	< 0.001

Table E. Variability of deficit measures among older subjects. Deficits included as components within a deficit index should exhibit increased frequency with age, but should not "saturate with age" and become universal among the oldest subjects within a cohort (Searle et al. 2008, BMC Geriatrics 8:24). To ensure that deficit variables we considered retained some level of variability among subjects with increasing age, we evaluated the Gini Index coefficient for each variable with respect to five SOF age cohorts (i.e., ages 65-69, 70-74, 75-79, 80-84 and 85-89). For a categorical variable with *n* classes, in which the respective class frequencies are p_1, p_2, …, p_n, the Gini Index is defined as 1- ($p_1^2 + p_2^2 + \ldots + p_n^2$). The Gini Index is thus an intuitive measure of heterogeneity for categorical variables, which ranges in value between 0 (no variability and uniformity among subjects) and 1 (maximal variability and heterogeneity among subjects) (for a review of this variability measure, see Tan, Steinbach and Kumar, *An Introduction to Dating Mining*, Addison Wesley, 2005). In the table below, the Gini Index coefficient is listed for each variable with respect to the five age cohorts. For each variable, Gini Index coefficients were non-zero among each of the five age cohorts, indicating that deficit variables were not uniform among certain age cohorts and that the variables did not saturate with age.

Variable	Age 65-69	Age 70-74	Age 75-79	Age 80-84	Age 85-89
CKR1	0.024	0.043	0.046	0.075	0.172
HH1	0.337	0.375	0.427	0.439	0.472
SH1	0.091	0.114	0.17	0.250	0.385
CLB1	0.217	0.287	0.328	0.371	0.476
STP1	0.172	0.219	0.268	0.297	0.444
WLK1	0.168	0.228	0.301	0.359	0.476
AUTOC	0.173	0.208	0.244	0.245	0.264
BENDC	0.194	0.214	0.253	0.258	0.189
RCHC	0.102	0.117	0.131	0.204	0.215
SCKONC	0.149	0.161	0.175	0.167	0.154
CHR	0.031	0.060	0.094	0.217	0.360
STPARM	0.092	0.161	0.225	0.428	0.499
AIDS	0.048	0.078	0.109	0.231	0.367
CHORHR	0.457	0.442	0.393	0.336	0.232
WTLS50	0.498	0.500	0.495	0.438	0.354
COMP	0.604	0.613	0.611	0.609	0.614
CMP12	0.416	0.430	0.469	0.484	0.481
INBED7	0.077	0.098	0.090	0.116	0.144
HWKINT	0.090	0.059	0.033	0.031	0.017
EXER	0.494	0.500	0.492	0.477	0.450
ANTDEP	0.071	0.080	0.057	0.045	0.067
ANXMED	0.309	0.314	0.308	0.266	0.245
CAROUS	0.675	0.650	0.642	0.640	0.646
SHT3MS	0.398	0.453	0.503	0.560	0.622
HYTEN	0.438	0.477	0.493	0.498	0.489
EARTH	0.477	0.467	0.435	0.438	0.439
EDIAB	0.125	0.134	0.145	0.132	0.091
EHIP	0.451	0.464	0.455	0.474	0.442

EHTHY	0.151	0.165	0.193	0.177	0.251
EOSTEO	0.220	0.251	0.273	0.311	0.334
EPARK	0.009	0.015	0.012	0.015	0.026
ESTRK	0.037	0.068	0.069	0.098	0.091
HSP	0.169	0.215	0.23	0.257	0.298
BCANC	0.082	0.102	0.095	0.139	0.158
SLPMED	0.282	0.313	0.321	0.347	0.372
GRPAVG	0.500	0.500	0.500	0.500	0.497
WLKSPD	0.500	0.470	0.409	0.242	0.129
TWTM	0.469	0.483	0.492	0.490	0.467
HRFC	0.499	0.466	0.413	0.330	0.186
BMI	0.653	0.640	0.638	0.613	0.607

(A) Distribution of deficit index (n = 9704 subjects, age 65−89)

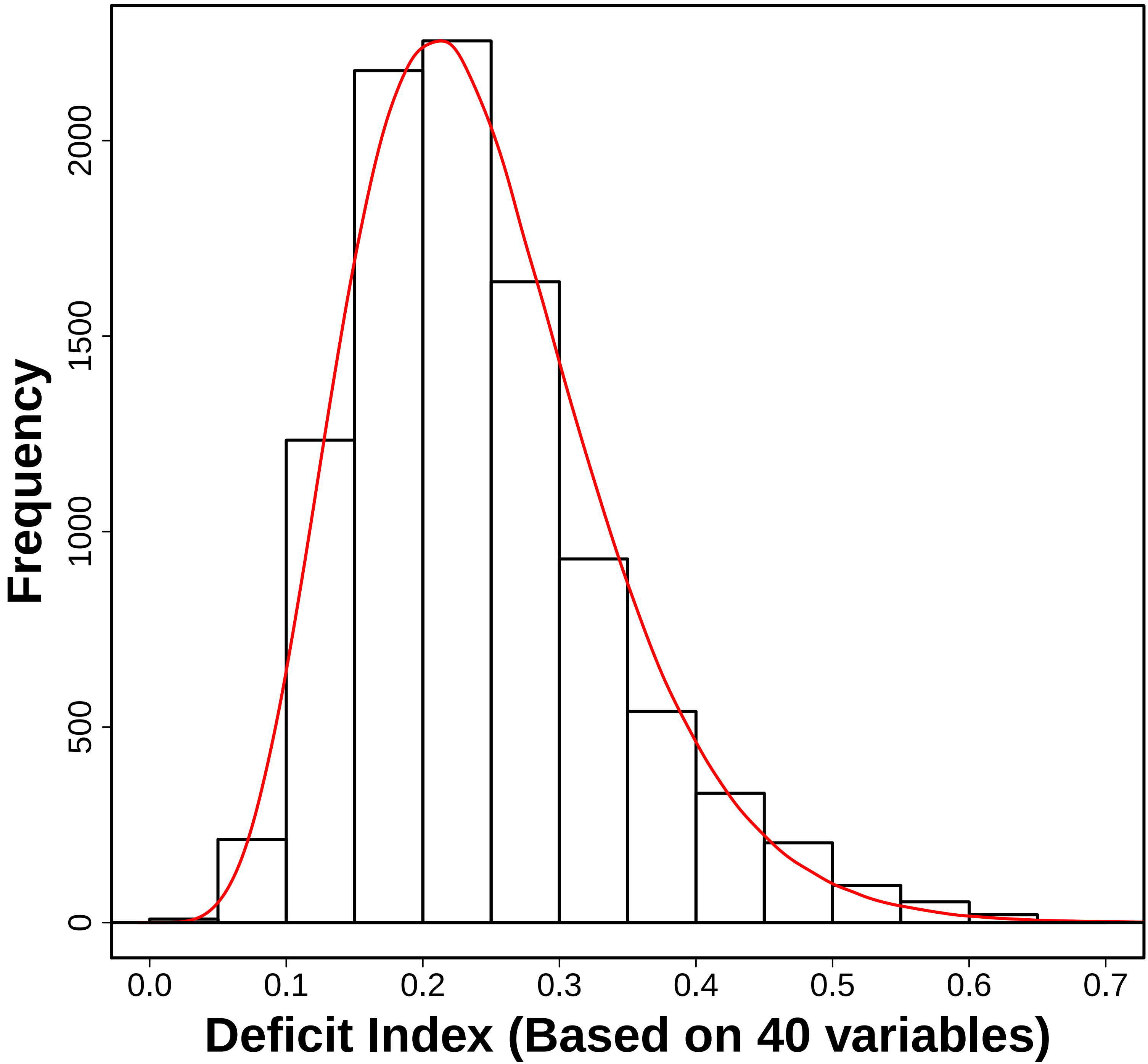

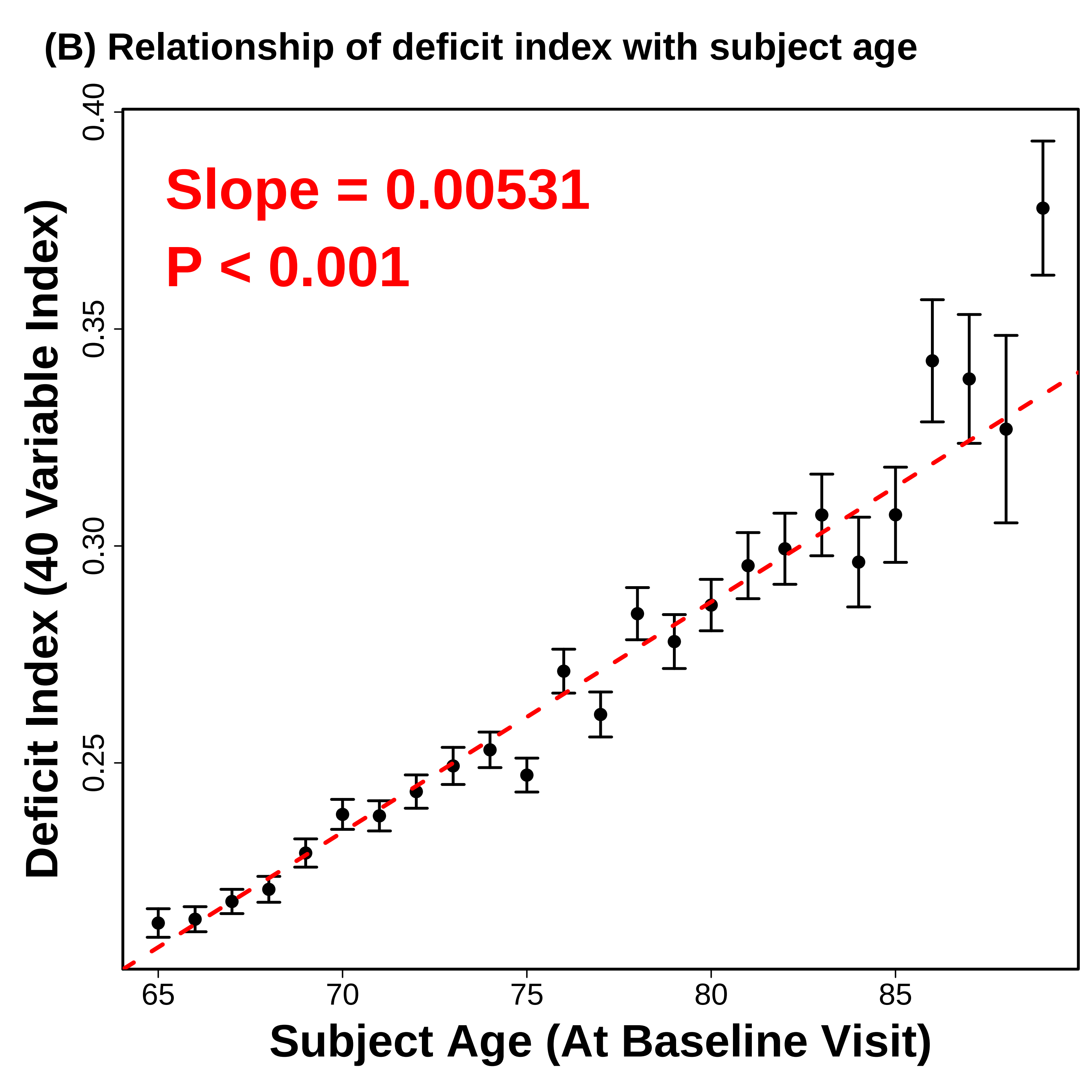

(B) Relationship of deficit index with subject age
Slope = 0.00531
P < 0.001
Deficit Index (40 Variable Index)
0.40
0.35
0.30
0.25
65
70
75
80
85
Subject Age (At Baseline Visit)

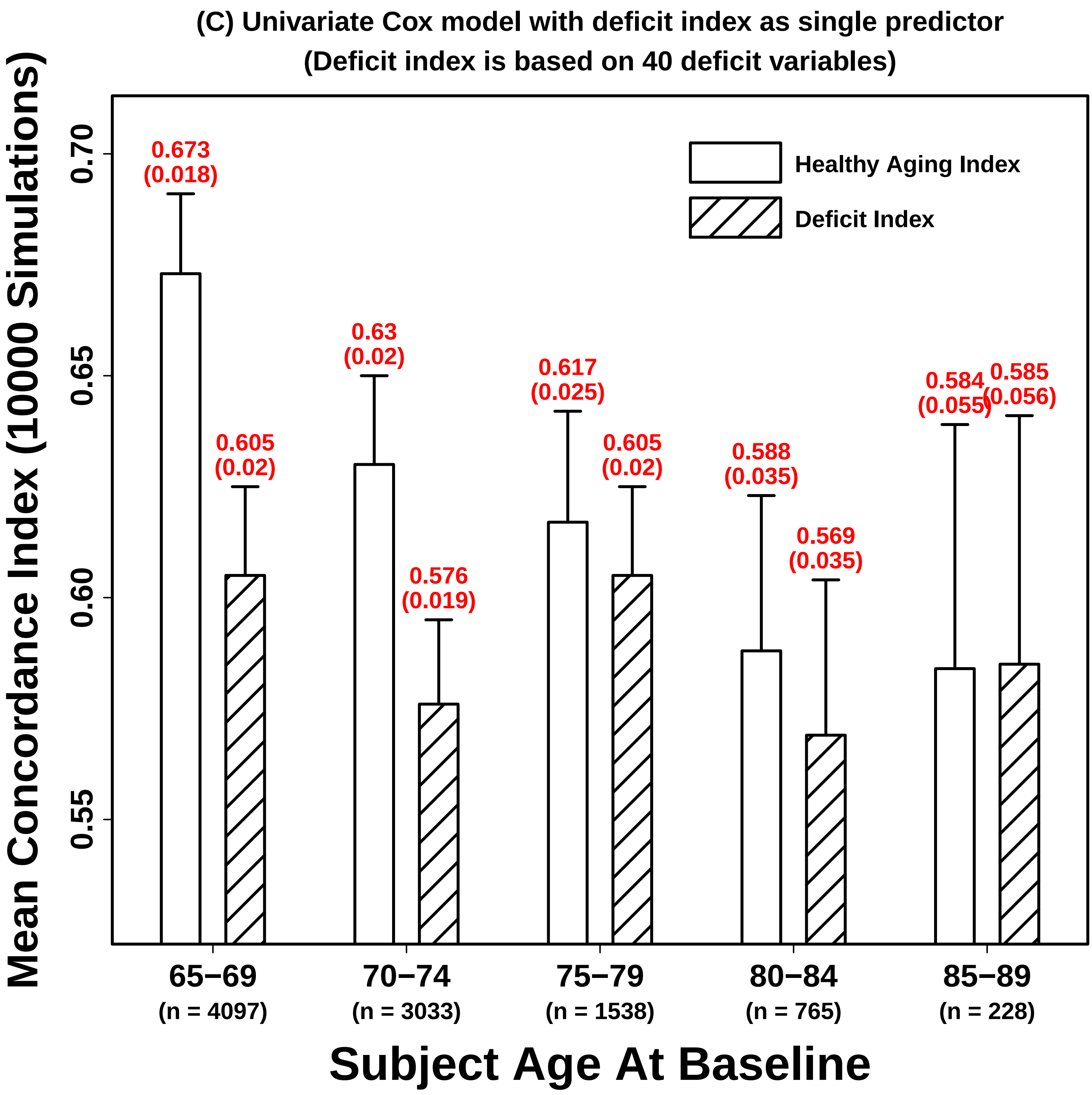
(C) Univariate Cox model with deficit index as single predictor
(Deficit index is based on 40 deficit variables)
Mean Concordance Index (10000 Simulations)
0.70
0.65
0.60
0.55
Healthy Aging Index
Deficit Index
0.673 (0.018)
0.605 (0.02)
0.63 (0.02)
0.576 (0.019)
0.617 (0.025)
0.605 (0.02)
0.588 (0.035)
0.569 (0.035)
0.584 (0.055)
0.585 (0.056)
65−69 (n = 4097)
70−74 (n = 3033)
75−79 (n = 1538)
80−84 (n = 765)
85−89 (n = 228)
Subject Age At Baseline

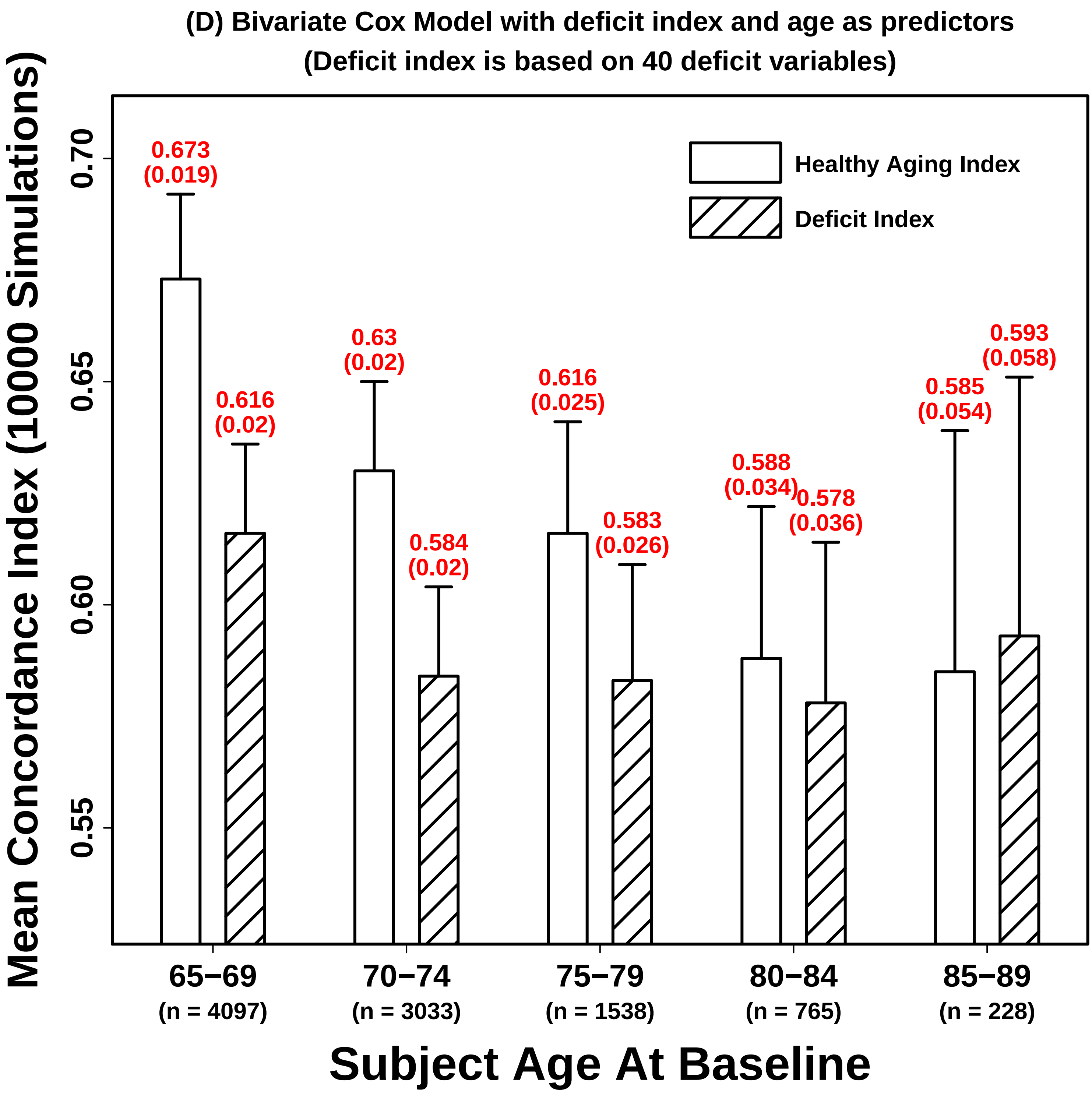

(D) Bivariate Cox Model with deficit index and age as predictors
(Deficit index is based on 40 deficit variables)
Mean Concordance Index (10000 Simulations)
0.55
0.60
0.65
0.70
Healthy Aging Index
Deficit Index
0.673 (0.019)
0.616 (0.02)
0.63 (0.02)
0.584 (0.02)
0.616 (0.025)
0.583 (0.026)
0.588 (0.034)
0.578 (0.036)
0.585 (0.054)
0.593 (0.058)
65−69 (n = 4097)
70−74 (n = 3033)
75−79 (n = 1538)
80−84 (n = 765)
85−89 (n = 228)
Subject Age At Baseline

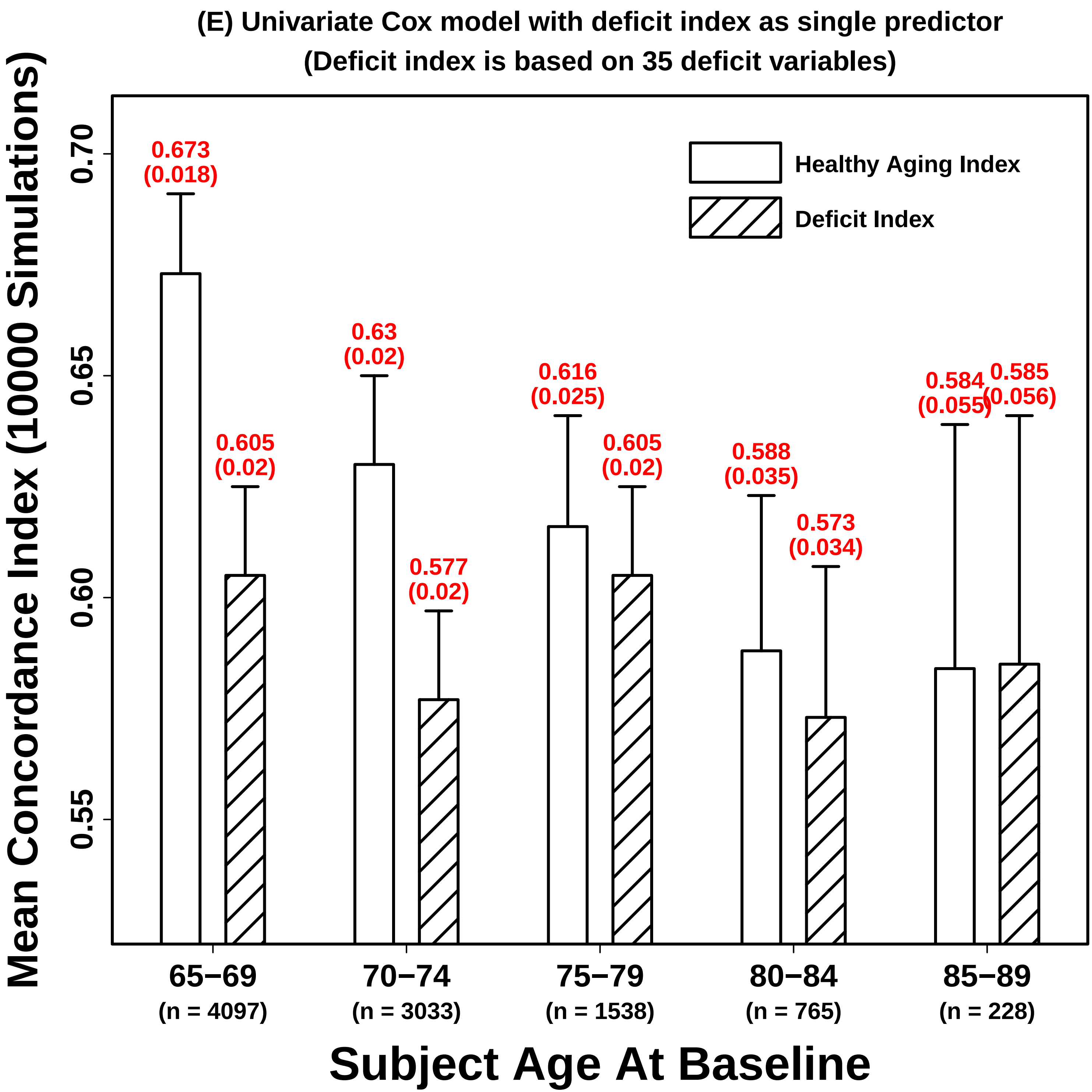
(E) Univariate Cox model with deficit index as single predictor
(Deficit index is based on 35 deficit variables)
Mean Concordance Index (10000 Simulations)
0.70
0.65
0.60
0.55
Healthy Aging Index
Deficit Index
0.673
(0.018)
0.605
(0.02)
0.63
(0.02)
0.577
(0.02)
0.616
(0.025)
0.605
(0.02)
0.588
(0.035)
0.573
(0.034)
0.584
(0.055)
0.585
(0.056)
65−69
(n = 4097)
70−74
(n = 3033)
75−79
(n = 1538)
80−84
(n = 765)
85−89
(n = 228)
Subject Age At Baseline

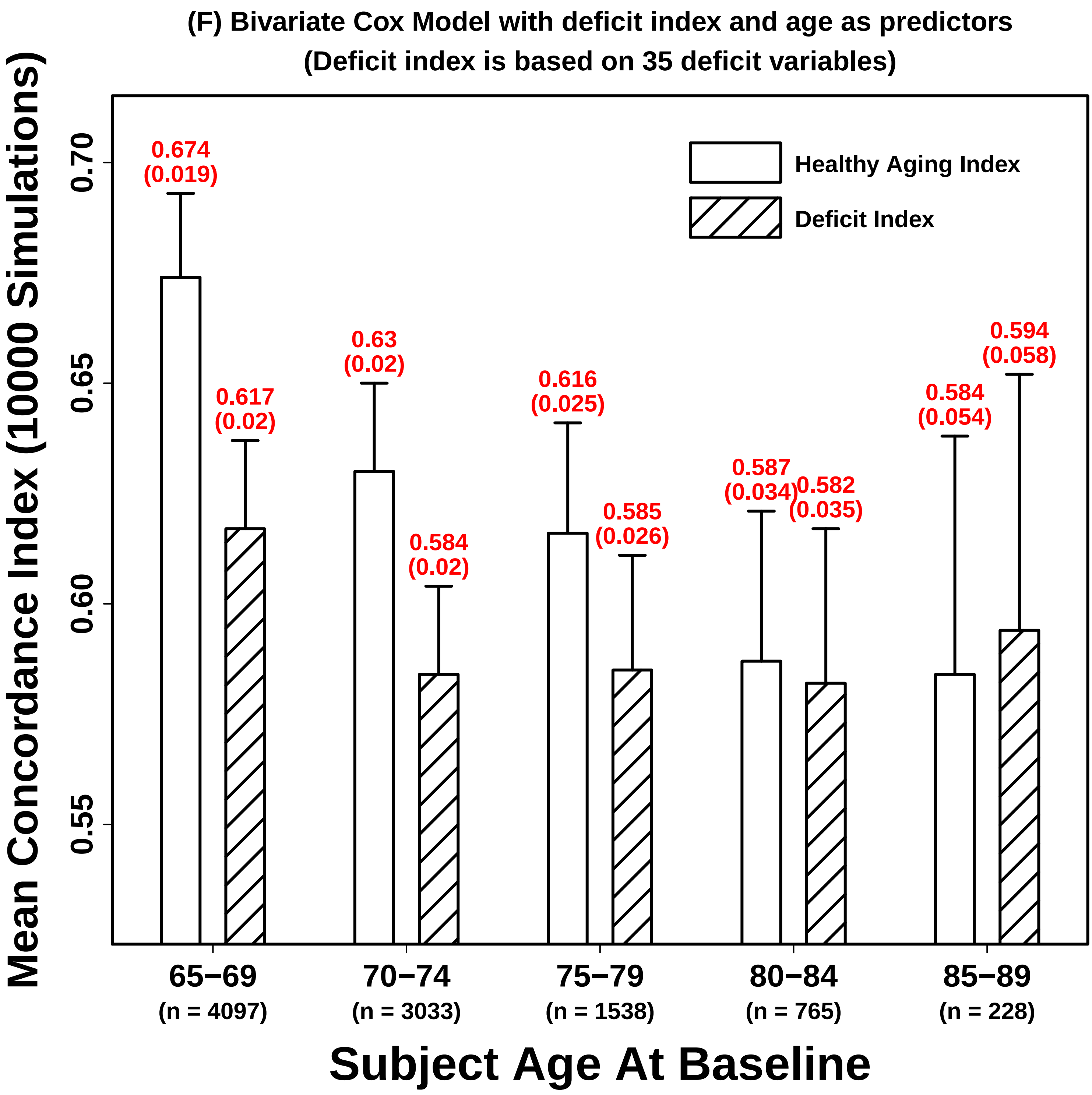
(F) Bivariate Cox Model with deficit index and age as predictors
(Deficit index is based on 35 deficit variables)
Mean Concordance Index (10000 Simulations)
0.70
0.65
0.60
0.55
Healthy Aging Index
Deficit Index
0.674
(0.019)
0.617
(0.02)
0.63
(0.02)
0.584
(0.02)
0.616
(0.025)
0.585
(0.026)
0.587
(0.034)
0.582
(0.035)
0.584
(0.054)
0.594
(0.058)
65−69
(n = 4097)
70−74
(n = 3033)
75−79
(n = 1538)
80−84
(n = 765)
85−89
(n = 228)
Subject Age At Baseline
